# S-100 B Concentrations Are a Predictor of Decreased Survival in Patients with Major Trauma, Independently of Head Injury

**DOI:** 10.1371/journal.pone.0152822

**Published:** 2016-03-31

**Authors:** Carmen Andrea Pfortmueller, Christian Drexel, Simone Krähenmann-Müller, Alexander Benedikt Leichtle, Georg Martin Fiedler, Gregor Lindner, Aristomenis Konstantinos Exadaktylos

**Affiliations:** 1 Clinic for General Anaesthesiology, Intensive Care and Pain Management, Vienna General Hospital and University of Vienna, Vienna, Austria; 2 Department of Emergency Medicine, University Hospital and University of Bern, Bern, Switzerland; 3 Centre of Laboratory Medicine, University Institute of Clinical Chemistry, Inselspital-Bern University Hospital, Inselspital, Bern, Switzerland; 4 Department of Emergency Medicine, Hirslandenklinik am Park Zurich, Zurich, Switzerland; University of Florida, UNITED STATES

## Abstract

**Background:**

Major trauma remains one of the principle causes of disability and death throughout the world. There is currently no satisfactory risk assessment to predict mortality in patients with major trauma. The aim of our study is to examine whether S-100 B protein concentrations correlate with injury severity and survival in patients with major trauma, with special emphasis on patients without head injury.

**Methods:**

Our retrospective data analysis comprised adult patients admitted to our emergency department between 1.12. 2008 and 31.12 2010 with a suspected major trauma. S-100 B concentrations were routinely assessed in major trauma patients.

**Results:**

A total of 27.7% (378) of all patients had major trauma. The median ISS was 24.6 (SD 8.4); 16.6% (63/378) of the patients died. S-100 B concentrations correlated overall with the ISS (p<0.0001). Patients who died had significantly higher S-100 B concentrations than survivors (8.2 μg/l versus 2.2 μg/l, p<0.0001). Polytraumatised patients with and without head trauma did not differ significantly with respect to S-100 B concentration (3.2 μg/l (SD 5.3) versus 2.9 μg/l (SD 3.8), respectively, p = 0.63) or with respect to Injury Severity Score (24.8 (SD 8.6) versus 24.2 (SD 8.1), respectively, p = 0.56). S-100 B concentrations correlated negatively with survival (p<0.0001) in all patients and in both subgroups (p = 0.001 and p = 0.006, respectively)

**Conclusions:**

S-100 concentrations on admission correlate positively with greater injury severity and decreased survival in major trauma patients, independently of the presence of a head injury. S-100 B protein levels at admission in patients with major trauma may therefore be used to assess outcome in all polytraumatised patients. These measurements should be subject to further evaluation.

## Introduction

Major trauma remains one of the world’s leading causes of disability and death [1–3]. In the USA, about 2.3 million hospitalisations and more than five million life years are lost per year due to major trauma [1,3]. In the European Union, 5.7 million hospitalisations and 233,000 fatalities are recorded annually [4]. Patients with multiple traumatic injuries often suffer from infection, organ failure and death, and this has encouraged the development of trauma-specific scoring systems [1]. Several risk assessment tools are currently used to estimated mortality in poly-traumatised patients, including the APACHE score (Acute Physiology and Chronic Health Evaluation) and the ISS (Injury Severity Score) [1]. But none of these clinical scores provides a satisfactorily accurate prediction of mortality in major trauma patients [1], and this has encouraged the search for new or additional risk assessment tools and biomarkers (e.g. neurone specific enolase or CK-BB).

S-100 B protein has recently received increasing attention as a possible biomarker for traumatic brain injury, as it was initially considered to be highly specific for brain tissue [5,6]. S100 proteins are a family of homodimeric cytosolic calcium binding proteins[7]. S-100 B is an isomer of S100 that is expressed in various cells and has multiple local regulatory effects, including cell division, proliferation, apoptosis, energy metabolism, and inflammation [8].

S-100 B protein occurs in high concentrations in astroglia and Schwann cells in the central nervous system [5]. Astrocytes are activated immediately after primary brain injury [9].

Even though it has been suggested that S-100 B protein is highly specific for traumatic brain injury, it has recently been demonstrated that S-100 B is also elevated in patients with major extracranial trauma [1,5,7,10]. Anderson et al reported that S-100 B protein concentrations may even be elevated in patients with a high energy trauma without brain injury [10].

No clinical study has yet evaluated the significance of S-100 B protein as a marker for trauma severity and survival in patients with major trauma. The aim of our study is to examine whether S-100 B concentrations correlate with injury severity and survival in patients with major trauma. In addition, it is assessed whether S-100B concentrations differ between poly-traumatised patients with and without brain injury.

## Material and Methods

### Setting

Our emergency department (ED) is the only Level I centre in a catchment area serving about 1.8 million people and treating more than 35,000 cases per year. On the basis of Advanced Traumatic Life Support (ATLS) guidelines and current recommendations, diagnostic and therapeutic management is at the discretion of the attending emergency physician. Despite slight variations in clinical practice between the physicians in our ED, the practical evaluation of patients with suspected major trauma generally follows the same pattern.

### Data collection and retrospective survey

Our retrospective data analysis comprised adult (≥16 years) patients admitted to our ED with a suspected major trauma between 1 December 2008 and 31 December 2010. S-100 B concentrations were routinely assessed in poly-traumatised patients as part of regular clinical work up. Calcium values were neither measured nor changed. All patients presenting to the ED with a suspected major trauma during the study period were eligible for study inclusion. They were identified using the appropriate search string in the entry diagnosis field of our computerised patient database (Qualicare Office, Medical Database Software, Qualidoc AG, Bern, Switzerland). Since this medical database allows instantaneous retrieval of past diagnostic reports, discharge summaries, consultations, radiographs and other relevant medical documents, the authors were able to retrospectively analyse the aetiology of the trauma, the diagnostic results, and therapeutic procedures initiated in the ED. The following clinical data were extracted from medical records: admission date, aetiology of the trauma, grade of traumatic brain injury (if any), type of traumatic brain injury, type of radiological imaging, radiological head imaging (yes/no), findings of radiological imaging, type of injury, hospitalisation, intensive care unit admission (ICU) primary and secondary (i.e. after initial discharge from the ED to the hospital ward), in-ED mortality, in-hospital mortality and time to death. Demographic data such as gender and age were also assessed. All medical records were reviewed by an internal specialist, a surgical specialist and a specialist in emergency medicine. Case aetiology and type of injuries were extracted according to diagnosis and medical history; no ICD 10 coding was used. The cause of trauma was categorised into four types (domestic, motor vehicle accident, sport, work). Each patient was only classified into one group, after agreement among the specialists. Falls in the house were defined as falls in the house itself or its close surroundings (e.g. garden). The type of injury was extracted from the diagnosis field and reconfirmed by review of the radiological image. Creatinine concentration (μmol/l) was extracted from laboratory records. In-hospital mortality was assessed by using data from our central hospital patient registry (SAP). Patients with neurological diseases (multiple sclerosis, stroke, seizure) (n = 126) and malignant tumours (n = 16) were excluded from the study, as these conditions are known to be associated with increased S-100 B protein levels (without trauma). Moreover patients without retrievable head imaging (n = 47) were excluded from the analysis.

### Assessment of trauma severity

All injuries were coded according to the AIS handbook 2008 and the Injury Severity Score (ISS) was calculated for each patient. According to the AIS, each injury is coded to eight different regions (head/neck, face, spine, thorax, abdomen/pelvic contents, upper extremity, lower extremity, external). Each injury is assigned an AIS severity code, ranging from 1 (minor) to 6 (maximal, unsurvivable) according to the handbook [11]. To calculate ISS, the scores for the three most severely injured body regions are squared and summed to produce the ISS score [12]. Brain injury severity was assessed by the Glasgow Coma Scale (GCS).

### Definition of major trauma

Major trauma or polytrauma was defined as ISS >15 [13].

### Traumatic brain injuries

Traumatic brain injury was defined as a traumatically induced structural injury and/or physiological disruption of brain function as a result of an external force, that is indicated by new onset or worsening of at least one of the following clinical signs, immediately following the event: loss of memory, amnesia, confusion or neurological deficits [14]. Additionally the results of the radiological imaging of the brain were reviewed. If no radiological image of the brain was taken, the patient was excluded from the study. Patients with neither a clinical sign of head injury (as described above) nor evidence of brain damage on the radiological image were classified into the non-head-injury group.

### Laboratory Testing

EDTA-anticoagulated whole blood samples were taken immediately on ED admission of each patient with suspected major trauma. Blood samples were centrifuged at 1000 g for 10 min in a storage tube with a bullet cover and the resulting plasma was collected. All of the obtained samples were split into aliquots and stored at minus 70°C until analysis. Laboratory testing was performed with quantitative ECLIA immunoassay (S-100 A1B and S-100 BB) by Roche Modular E170 in human serum.

### Definition of cut-off for survival analysis

The cut-off of 2.0 μg/l S-100 B concentration was chosen because S-100 B concentrations above 2.0 μg/l have been significantly associated with decreased survival after major head injury [15].

### Statistical Analysis

All statistical analyses were performed with the SPSS 20.0 Statistical Analysis program (SPSS Inc; Chicago, IL). The data were summarised using descriptive statistics (means and standard deviation or medians and interquartile range as appropriate, counts and percentages). Differences in characteristics and outcome between patients with and without head injury were tested using chi-squared tests for categorical variables and Student'st test for interval and ordinal variables. Correlation was assessed by Pearson and Spearman's correlation where appropriate. Survival was estimated by the Kaplan-Meier analysis and between-group differences were determined by the log rank test. Multivariable logistic regression was used to identify predictors for in-hospital mortality. The predefined variables added to the model were: age, gender, S-100 B concentration, head injury/no-head injury. All p values were two tailed and at a level of significance of 0.05.

### Ethical considerations

The study was approved by the Ethics Committee of the Canton of Bern, Switzerland. Individual patient consent was not obtained, but was waived by the Ethics Committee. Patient records/information was anonymised and de-identified prior to analysis.

## Results

A total of 1367 patients were included in the analysis. 996 (72.9%) of these were males and 371 (27.1%) females. For an overview of patient characteristics, see Table 1. The median age was 43 years (interquartile range 27–60). The mean ISS was 11 (standard deviation (SD) 9), with a mean S-100 B concentration of 1.5 μg/ml (SD 3.33).

**Table 1:** Patient Characteristics.

	N	%
	1367	100
Male (%)	996	72.9
Female (%)	371	27.1
Median age (interquartile range)	43 (27–60)	
*Mechanism of Trauma*		
Motor vehicle accident	541	39.6
Work	322	23.6
Domestic	241	17.7
Sport	363	19.2
*Trauma Severity*		
Mean ISS (SD)	11 (9)	
Polytrauma (%)	378	27.7
*Laboratory Parameters*		
Mean S-100 (SD), μg/l	1.58 (3.33)	
Mean creatinine (SD) μmol/l	72 (15)	
*Hospitalisation*		
In-patients (%)	1282	93.8
ICU (%)	435	31.8
In-hospital mortality	79	5.8

In total, 378 (27.7%) of all patients were poly-traumatised. The median ISS was 24.6 (SD 8.4). 16.6% (63/378) of the patients died. The mean time to death was 1.69 days (SD 2.4). Of all patients with major trauma, 266 (70.4%) suffered from an injury to the head and 112 (29.6%) had no head injury. There were no differences in sex or age (p = 0.98, or p = 0.28, respectively) with and without head injuries. For an overview of poly-traumatised patients with and without head injury see Table 2.

**Table 2:** Overview of patients with major trauma.

	Polytrauma with head injury	Polytrauma without head injury	p value
N (%)	266 (70.4)	112 (29.6)	
Male (%)	214 (80.4)	90 (80.3)	0.98
Female (%)	52 (19.6)	22 (19.7)	0.98
Median age (interquartile range)	48 (27–60)	45 (29–58)	0.28
*Trauma Severity*			
Mean ISS (SD)	24.8 (8.6)	24.2 (8.1)	0.56
Mean AIS Score (SD)			
Head	2.9 (0.07)	0 (0.0)	
Face	1.89 (0.74)	1.5 (0.68)	0.031
Spine	2.47 (1.09)	2.85 (1.24)	0.05
Thorax	2.88 (0.98)	3.04 (0.96)	0.26
Abdomen	2.68 (1.08)	2.77 (1.09)	0.66
Upper extremity	1.82 (0.47)	1.9 (0.62)	0.37
Lower extremity	2.1 (0.97)	2.79 (1.03)	0.0001
External	2.12 (1.12)	2.86 (2.03)	0.39
*Laboratory Parameters*			
Mean S-100 (SD), μg/l	3.2 (5.3)	2.9 (3.8)	0.63
Mean creatinine (SD) μmol/l	68 (8)	73 (17)	0.23
*Outcome*			
Overall ICU admission (%)	203 (76.3)	63 (56.3)	0.0001
Primary ICU admissions (%)	102 (50.2)	41 (65.1)	0.041
Secondary ICU admission	101 (49.8)	22 (34.9)	0.026
Overall mortality	52 (19.5)	11 (9.8)	0.035
In-emergency department mortality	4	0	0.0001
In-hospital mortality	48 (18.0)	11 (9.8)	0.045
Mean days to death (SD) d	1.76 (2.35)	1.36 (3.00)	0.62

**Fig 1:**
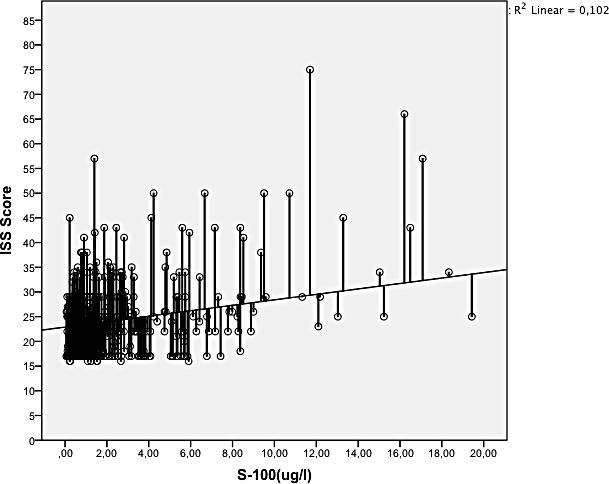
Correlation between S-100 B concentration and ISS (0.0001).

**Fig 2:**
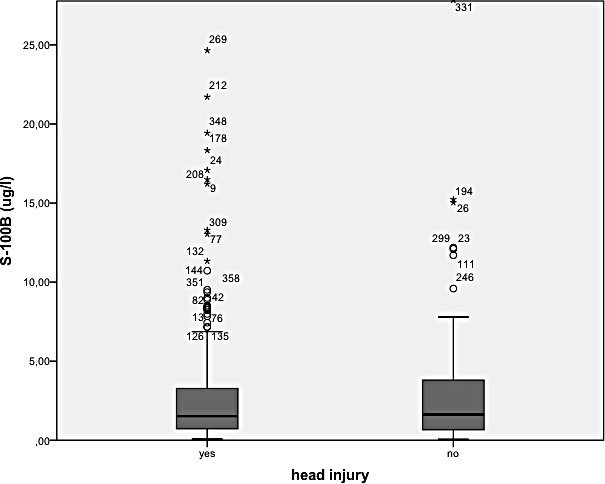
Patients with and without head trauma and S-100 B concentration(p = 0.63).

**Fig 3:**
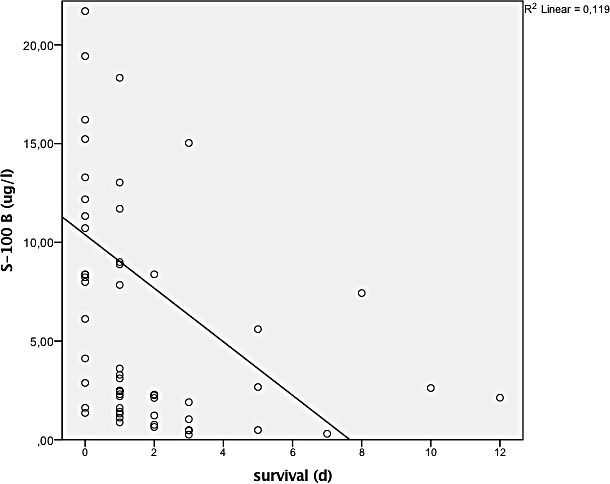
Correlation between survival und S-100 B concentration (p< 0.0001).

**Fig 4:**
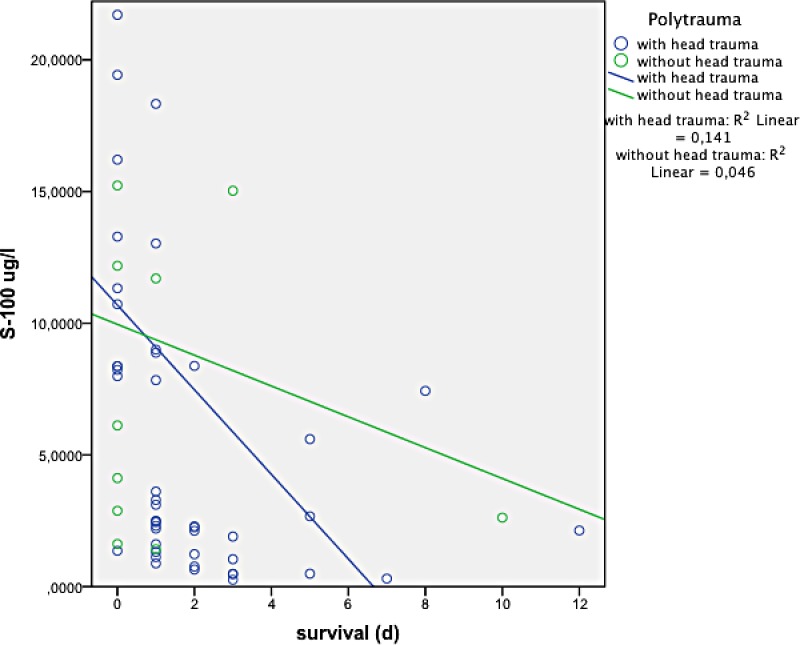
Relationship between S-100 B concentration and survival in patients with and without head injury (p = 0.001 and p = 0.006, respectively).

There was an overall positive correlation between S-100 B concentration and ISS (p<0.0001) (see Fig 1), as well as with the Abbreviated Injury Scale (AIS) of the lower extremity (p = 0.018) and of the thorax (p = 0.034). There was no correlation with AIS of the head (p = 0.31). Patients who died had a significantly higher S-100 B concentration than survivors (8.2 μg/l versus 2.2 μg/l, p < 0.0001). Polytraumatised patients with and without head trauma did not significantly differ with respect to S-100 B concentration (3.2 μg/l (SD 5.3) versus 2.9 μg/l (SD 3.8), p = 0.63) or ISS (24.8 (SD 8.6) versus 24.2 (SD 8.1), p = 0.56). See Fig 2. S-100 B concentration correlated negatively with survival (p < 0.0001) in all patients and in the subgroups with and without head injury (p < 0.001, p = 0.006, respectively), see Figs 3 and 4. Patients with head injury did not die significantly earlier than patients without head injury (1.4 days versus 1.8 days, p = 0.62).

**Fig 5:**
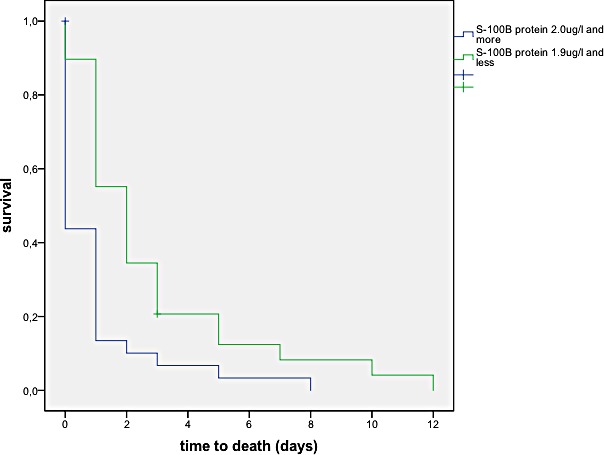
S-100 B concentrations above 2.0 μg/l are associated with decreased survival (p = 0.01).

Kaplan-Meier analysis showed that a S-100 B concentration above 2.0 μg/ml was significantly associated with decreased survival (p = 0.01), see Fig 5.

## Discussion

The aim of our study was to examine whether S-100 B concentrations correlate with injury severity and survival in patients with major trauma. In addition, it was assessed whether S-100B concentrations differ between poly-traumatised patients with and without brain injury.

Our study shows that S-100 B concentration is associated with decreased survival in patients with major trauma. S-100 B concentrations were markedly higher in fatal cases and survival was lower. In a pathology study on S-100 B concentrations as a possible biomarker for severe injury, the authors found markedly higher S-100 B concentrations in patients suffering from major trauma when compared to other fatalities, but did not assess survival [1]. In their laboratory analysis, they found a positive correlation between S-100 B concentration and markers of endothelial injury, such as van Willebrand factor [1]. Additionally, an increase in S-100 B concentration was found to be associated with a 33% increase in the death rate of endothelial cells compared to healthy individuals and was accompanied by increased levels of pro-inflammatory cytokines [1,16]. This suggests that endothelial cell apoptosis accelerates pro-inflammatory pathways—the major contributor to the development of the serious complications of major trauma, such as systemic inflammatory response or multiorgan failure [1,17]. Additionally a recent study by Stamataki et al on S-100 B protein and haemorrhage found that S-100 B protein was significantly associated with hypoperfusion and major haemorrhage and decreased survival in patients undergoing an emergency operation for haemorrhage control [18].This may explain why S-100 B concentration is linked with decreased survival.

We did not find any relationship between the severity of trauma to the head (AIS head) and S-100 B concentrations, as reported in other studies [5,7,15,19,20]. In addition, our patients with head injury died earlier than patients without head injury, even though there was no significant difference in time to death between the head injury and non-head injury group. It is still unclear whether S-100 B concentration is associated with an increased risk of death in patients with head injury [5,6,16,18,21,22].

S-100 B protein is highly specific for the activation or death of astrocytes and oligodendrocytes [7,21]. On the other hand, a recent study by Shakeri et al on S-100 B protein as a post-traumatic biomarker for prediction of brain death found no association between S-100 B levels and death when S-100 B was measured within the first six hours after trauma [6]. It has to be noted that the study by Shakeri et al was the first study to assess S-100 B concentrations in severely injured patients with exclusive head trauma; all patients with major tissue damage or bone fractures were excluded [6]. Some other studies on S-100 B concentrations and outcome also included patients with major tissue damage or fractures and showed decreased survival [7,15,22,23]. As our study clearly shows, S-100 B concentration is associated with decreased survival in patients with major trauma, independently of head injury.

In our study, S-100 B concentrations were significantly associated with injury severity (ISS). Our results are similar to those of a study by Dang et al on 122 traumatised patients [1]. A study by Savola et al on the effects of head and extracranial injuries on serum S-100 B levels in trauma patients likewise found a correlation between injury severity and S-100 B concentration [5]. S-100 B concentrations in healthy volunteers are markedly lower than in trauma patients [1].

Our results found no statistically significant difference in S-100 B concentrations between patients with and without head injury. A study by Savola et al compared the effects of head and extracranial injuries and found elevated S-100 B concentrations in both populations, but with significantly higher S-100 B concentrations in the head injury group [5]. As in the present study, Dang and Anderson et al found no difference in S-100 B concentrations between patients with and without head injury [1,10]

The reason that patients without head injury also have elevated S-100 B concentrations may be that they suffer major soft tissue damage and bone fractures and that these also lead to increased S-100 B concentrations, as described in some studies [1,5,10,24,25].

In our study, patients without head trauma had a significantly higher AIS of the lower extremity than the head injury group, a fact that may reinforce this hypothesis. A study by Pelinka et al on S-100 B protein levels with femoral fractures in rats without head injury found significantly increased S-100 B concentrations [24]. S-100 B protein was increased in these rats independently of concomitant haemorrhage, and this suggests that S-100 B protein may also be produced in the bone marrow [24]. The significantly elevated AIS for the lower extremity in patients without brain trauma in our study may therefore be caused by a combination of increased S-100 B protein release from the marrow of large fractured bones and increased haemorrhage in fractured large bones, such as the femur or the pelvis. We also found higher AIS of the thorax in patients without head injury, as confirmed by Dang et al, who found that contusions to the thorax without rib fractures lead to substantially elevated S-100 B concentrations [1].

### Limitations

Our study is limited by its retrospective, single centre design. As information in our medical history database is presented in a narrative comment, no guarantee of complete or correct reporting can be given and information bias is possible. Moreover, we only assessed S-100 B serum concentrations on admission and are therefore not able to reach any conclusion about changes in S-100 B concentration over time and how this is may be related to survival. Furthermore we did not assess the clinical history leading to death (for example systemic inflammatory response syndrome, multi-organ dysfunction or bleeding). In addition, our study was limited to adults (>16 years of age), as children are treated at a separate emergency department in our hospital.

## Conclusion

S-100 concentration on admission is of diagnostic value in the evaluation of injury severity and survival of major trauma patients. S-100 B concentrations are essentially the same with and without head injury in patients with major trauma. S-100 B concentrations above 0.2 μg/l are associated with decreased survival in all patients, regardless of concomitant head trauma.

Further studies should be conducted to assess the relationship between S-100 B concentration and SIRS and MOD. Additionally, further studies should more specifically target S-100 B concentrations in patients with major trauma without head injury.

## Supporting Information

S1 Table Supporting Material. (PDF)
