# Supplementary material for: S-100 B Concentrations Are a Predictor of Decreased Survival in Patients with Major Trauma, Independently of Head Injury
Source: PLoS One. 2016 Mar 31;11(3):e0152822. doi: 10.1371/journal.pone.0152822 (PMC4816449; doi:10.1371/journal.pone.0152822)
Supplement: S1 Table — (PDF) [file pone.0152822.s001.pdf]

Alter	Sex	Jahr	Austrittsort	GCS
55	M	2008		13-15
29	M	2008		keines
45	M	2008		13-15
16	F	2008		13-15
32	M	2008		keines
38	F	2008		13-15
38	M	2008	IMC	9-12
34	F	2008		13-15
63	M	2008	OP	13-15
20	M	2008		13-15
44	F	2008		13-15
45	F	2008	OP	keines
17	F	2008		13-15
54	M	2008		13-15
25	F	2008	OP	9-12
41	M	2008	Normalstation	13-15
76	M	2008		13-15
23	F	2008	OP	13-15
52	M	2008	Normalstation	9-12
23	M	2008	nach Hause	13-15
74	M	2008	OP	13-15
45	M	2008	Normalstation	13-15
74	M	2008		keines
32	M	2008		13-15
81	M	2008		3-8
76	F	2008		
68	M	2008		3-8
51	F	2008		keines
19	F	2008		13-15
79	F	2008	Normalstation	13-15
16	M	2008		13-15
73	M	2008	Normalstatio	13-1
32	M	2008		13-1
18	M	2008		13-1
30	M	2008	Normalstatio	kein
76	F	2008		13-1
49	M	2008		13-1
17	M	2008		3-
52	F	2008		13-1
54	F	2008		3-
16	M	2008		kein
71	F	2008		13-1
21	M	2008	nach Hau	13-1
60	F	2008		kein
45	M	2008		kein

53	M	2008		13-1
40	M	2008		13-1
71	M	2008		3-
49	M	2008		13-1
44	M	2008		13-1
30	M	2008		13-1
28	M	2008		13-1
23	F	2008		3-
78	F	2008		13-1
25	M	2008		13-1
23	M	2008		13-1
50	F	2008		3-
33	M	2008		13-1
49	F	2008		kein
88	F	2008		kein
34	F	2008		13-1
79	M	2008		13-1
90	M	2008		13-1
39	M	2008		kein
72	M	2008		9-
66	M	2008		13-1
65	M	2008		
89	M	2008	Normalstatio	13-1
86	F	2008		13-1
88	M	2008	Normalstatio	13-1
22	F	2008		13-1
26	M	2008		13-1
31	M	2008		kein
43	F	2008		13-1
25	M	2008		3-
63	M	2008		13-1
21	F	2008		kein
27	M	2008		13-1
83	M	2008		13-1
15	M	2008		3-
60	M	2008		kein
24	M	2008		13-1
82	M	2008		13-1
62	M	2008		13-1
25	M	2008		13-1
28	F	2008		13-1
29	F	2008		13-1
61	F	2008		kein
54	M	2008		13-1
19	M	2008		3-
30	M	2008		13-1

78	F	2008		13-1
75	F	2008		13-1
28	M	2008		13-1
68	M	2008		9-
51	M	2008		13-1
20	F	2008		13-1
76	F	2008	Normalstatio	13-1
83	M	2008		13-1
50	F	2008		13-1
71	M	2008		
54	M	2008		kein
19	F	2008		13-1
40	M	2008		3-
57	F	2008		13-1
61	M	2008		13-1
20	M	2008		13-1
83	M	2008		3-
24	F	2008		3-
46	F	2008		13-1
38	M	2008	Normalstatio	kein
21	F	2008		kein
32	M	2008		13-1
27	M	2008		kein
35	M	2008		13-1
74	F	2008		kein
29	M	2008		13-1
52	M	2008		kein
44	M	2008		kein
49	M	2008		13-1
25	M	2008		kein
50	F	2008		kein
17	F	2008		kein
51	M	2008		kein
18	F	2008		13-1
18	M	2008		13-1
61	M	2008		13-1
50	M	2008		3-
69	M	2008		13-1
84	M	2008		13-1
27	M	2008		kein
32	F	2008		
22	F	2008		13-1
49	M	2008		13-1
40	F	2008		13-1
83	M	2008		
55	M	2008		3-

32	M	2008	13-1
53	M	2008	3-
53	F	2008	13-1
21	M	2008	13-1
71	F	2008	13-1
80	M	2008	kein
75	M	2008	kein
19	M	2008	3-
31	M	2008	13-1
44	M	2008	kein
82	F	2008	kein
61	M	2008	13-1
72	M	2008	3-
17	M	2008	13-1
46	M	2008	13-1
55	M	2008	13-1
37	M	2008	13-1
68	M	2008	kein
63	M	2008	kein
26	M	2008	kein
52	M	2008	kein
20	F	2008	13-1
38	F	2008	13-1
19	M	2008	kein
22	M	2008	3-
56	M	2008	13-1
46	M	2008	3-
18	M	2008	13-1
58	M	2008	13-1
26	M	2008	kein
71	M	2008	kein
61	M	2008	kein
53	M	2008	3-
19	F	2008	13-1
45	F	2008	13-1
37	M	2008	13-1
26	F	2008	13-1
44	M	2008	kein
17	M	2008	kein
48	M	2008	13-1
47	M	2008	kein
35	F	2008	13-1
17	M	2008 IM	13-1
31	M	2008	kein
43	F	2008 IM	13-1
49	F	2008 IM	13-1

45	F	2008		kein
51	M	2008		13-1
18	M	2008		13-1
32	F	2008		13-1
16	M	2008		13-1
84	F	2008		13-1
60	M	2008		kein
27	M	2008		kein
53	M	2008		13-1
20	M	2008		13-1
31	M	2008		13-1
33	F	2008		kein
80	F	2008		kein
20	F	2008		kein
24	M	2008		13-1
74	F	2008		13-1
47	F	2008		kein
42	F	2008		kein
66	M	2008	IM	9-
23	M	2008		kein
21	M	2008		13-1
31	M	2008		9-
25	M	2008		13-1
35	M	2008		
74	M	2008		13-1
54	M	2008		kein
47	M	2008		kein
47	M	2008		13-1
71	F	2008		13-1
69	M	2008		13-1
83	M	2008		13-1
69	M	2008	IM	kein
61	M	2008		9-
71	F	2008		13-1
48	F	2008		13-1
49	M	2008	Normalstatio	kein
25	M	2008		13-1
67	M	2008		13-1
37	M	2008		kein
28	M	2008		kein
59	M	2008		13-1
53	M	2008		kein
22	M	2008		13-1
34	M	2008		13-1
57	M	2008		kein
42	M	2008		kein

71	M	2008		kein
20	M	2008		3-
42	M	2008		kein
28	M	2008		13-1
16	M	2008		kein
43	M	2008		13-1
43	M	2008		13-1
82	M	2008		13-1
38	M	2008		13-1
25	F	2008		13-1
61	M	2008	nach Hau	13-1
30	M	2008		13-1
51	M	2008		13-1
64	M	2008		kein
43	M	2008		kein
26	F	2008		3-
22	M	2008	nach Hau	13-1
38	M	2008		kein
26	F	2008		13-1
59	F	2008		9-
41	M	2008		kein
46	F	2008		9-
16	M	2008		13-1
89	F	2008	nach Hau	kein
46	M	2008		kein
70	F	2008		9-
75	M	2008		13-1
68	M	2008		kein
20	M	2008		kein
47	F	2008		13-1
64	M	2008		kein
61	M	2008		13-1
37	M	2008		kein
35	F	2008		
78	M	2008		
39	M	2008		kein
35	M	2008		kein
17	M	2008		13-1
54	M	2008		kein
61	F	2008		kein
68	M	2008		13-1
58	M	2008		9-
34	M	2008		13-1
22	M	2008		13-1
66	F	2008		
55	M	2008		kein

48	M	2008		3-
63	M	2008		3-
33	M	2008		13-1
78	M	2008		13-1
33	M	2008		13-1
16	F	2008		3-
58	M	2008		13-1
27	M	2008		13-1
59	M	2008		9-
31	M	2008		13-1
34	M	2008		13-1
26	F	2008		kein
78	M	2008		kein
75	M	2008		kein
29	F	2008		kein
31	M	2008		13-1
34	M	2008	nach Hau	kein
59	M	2008		13-1
22	M	2008		13-1
28	M	2008		kein
39	F	2008		kein
33	M	2008		9-
61	M	2008		13-1
61	M	2008		9-
28	M	2008		9-
33	M	2008		kein
42	M	2008	Normalstatio	13-1
18	F	2008		9-
51	F	2008		13-1
16	F	2008		kein
43	M	2008		9-
36	M	2008	Normalstatio	kein
17	M	2008		13-1
51	F	2008		13-1
65	F	2008		3-
26	F	2008		13-1
40	M	2008		kein
34	M	2008		13-1
46	M	2008		13-1
44	M	2008		kein
34	M	2008		kein
67	M	2008	Normalstatio	kein
19	M	2008		kein
26	F	2008		13-1
19	M	2008		13-1
30	M	2008		kein

23	F	2008		kein
28	M	2008		13-1
30	M	2008		kein
42	M	2008		9-
56	F	2008	Normalstatio	kein
43	M	2008		kein
53	F	2008	Normalstatio	13-1
24	M	2008		13-1
49	M	2008		9-
56	M	2008		kein
32	M	2008	nach Hau	kein
57	M	2008		kein
60	M	2008		3-
40	M	2008		kein
82	F	2008		kein
17	M	2008		kein
48	F	2008		13-1
19	F	2008		13-1
72	M	2008	Normalstatio	13-1
46	M	2008		3-
49	M	2008	Normalstatio	13-1
44	M	2008	Normalstatio	kein
35	F	2008		13-1
31	M	2008		13-1
43	M	2008		13-1
22	M	2008		kein
22	M	2008	Normalstatio	kein
49	M	2008	IM	13-1
76	M	2008		kein
87	F	2008	nach Hau	kein
77	M	2008		kein
24	M	2008		13-1
67	M	2008		kein
25	F	2008	Normalstatio	kein
27	F	2008		9-
54	M	2008	IM	kein
75	F	2008		13-1
60	M	2008		13-1
35	M	2008		13-1
23	M	2008		3-
45	F	2008		13-1
35	M	2008		13-1
18	M	2008		kein
26	F	2008		kein
63	M	2008		13-1
21	M	2008		kein

23	M	2008		13-1
66	F	2008		13-1
46	F	2008	IM	3-
17	M	2008		13-1
77	F	2008		13-1
47	M	2008		kein
27	M	2008		kein
38	M	2008		3-
26	F	2008		kein
47	M	2008		kein
26	F	2008		kein
52	M	2008	nach Hau	13-1
17	M	2008		13-1
19	M	2008		kein
56	F	2008		13-1
52	M	2008		13-1
53	M	2008		13-1
69	F	2008		13-1
31	F	2008		13-1
17	M	2008		13-1
87	M	2008		3-
63	M	2008		13-1
53	M	2008		kein
46	M	2008		3-
28	F	2008		3-
46	M	2008	nach Hau	13-1
42	F	2008		13-1
55	M	2008	nach Hau	9-
74	F	2008		3-
42	F	2008		
21	M	2008		kein
30	M	2008		kein
25	F	2008		13-1
61	M	2008		13-1
18	F	2008		13-1
22	M	2008	IM	9-
31	M	2008		13-1
20	M	2008		13-1
30	F	2008		13-1
46	F	2008		kein
91	M	2008		13-1
29	M	2008		13-1
22	M	2008		kein
29	M	2008	Normalstatio	13-1
19	F	2008		kein
20	M	2008	nach Hau	13-1

21	M	2008	IM	13-1
32	M	2008		13-1
36	F	2008		kein
44	F	2008		9-
28	M	2008	nach Hau	13-1
22	M	2008		13-1
17	F	2008		13-1
41	M	2008		13-1
19	M	2008		13-1
23	M	2008		kein
21	M	2008		13-1
19	F	2008	IM	kein
61	M	2008		13-1
24	F	2008		13-1
30	M	2008		kein
32	F	2008		13-1
21	M	2008		13-1
20	F	2008		13-1
21	M	2008	nach Hau	13-1
22	F	2008	IM	13-1
25	M	2008		3-
31	M	2008		13-1
36	F	2008		kein
67	F	2008		13-1
40	F	2008		kein
61	M	2008		13-1
20	M	2008		kein
42	M	2008		13-1
33	M	2008	nach Hau	kein
75	M	2008		13-1
64	M	2008		13-1
83	F	2008		13-1
25	M	2008		3-
54	M	2008		13-1
73	M	2008		13-1
66	M	2008		13-1
41	F	2008		13-1
34	F	2008		13-1
75	F	2008		13-1
34	F	2008	nach Hau	13-1
40	M	2008		3-
75	M	2008		3-
72	F	2008		13-1
40	M	2008		13-1
36	M	2008		13-1
56	M	2008		kein

43	M	2008		13-1
85	M	2008		13-1
56	F	2008		3-
79	F	2008		13-1
41	M	2008		13-1
22	M	2008		13-1
68	F	2008		13-1
32	M	2008		13-1
20	M	2008		13-1
20	M	2008		3-
60	M	2008		13-1
60	M	2008		kein
17	M	2008		13-1
58	M	2008		kein
61	F	2008		3-
97	F	2008		13-1
63	F	2008		13-1
57	M	2008		3-
84	F	2008		13-1
82	F	2008		3-
20	M	2008		kein
65	M	2008		3-
34	F	2008		13-1
72	M	2008		13-1
30	M	2008		3-
43	M	2008		3-
24	F	2008		13-1
52	M	2008		kein
25	M	2008		13-1
70	F	2008		3-
46	M	2008		kein
42	F	2008		9-
74	F	2008	nach Hau	kein
26	M	2008		kein
39	F	2008		13-1
52	M	2008		13-1
60	M	2009		13-1
21	F	2009		3-
75	M	2009		9-
22	M	2009		
80	F	2009		13-1
24	M	2009		13-1
43	M	2009		3-
41	M	2009		13-1
52	F	2009		13-1
57	F	2009		13-1

82	M	2009		13-1
17	F	2009		kein
58	M	2009		13-1
59	M	2009	IM	13-1
22	F	2009	nach Hau	13-1
33	F	2009	nach Hau	13-1
47	M	2009		3-
37	M	2009		13-1
55	M	2009		13-1
28	M	2009		3-
62	M	2009		3-
33	M	2009		3-
22	F	2009	nach Hau	13-1
53	M	2009		13-1
60	F	2009		kein
33	F	2009		3-
21	M	2009		9-
17	M	2009		13-1
44	M	2009		3-
22	F	2009	nach Hau	13-1
29	M	2009		3-
54	M	2009		13-1
52	F	2009		kein
73	M	2009		13-1
28	M	2009		13-1
55	M	2009		9-
66	M	2009		13-1
34	M	2009		kein
36	F	2009		13-1
18	M	2009	Normalstatio	13-1
43	M	2009		3-
81	M	2009		13-1
21	M	2009	Normalstatio	13-1
76	M	2009		3-
53	F	2009		13-1
52	F	2009	Normalstatio	13-1
39	M	2009	Normalstatio	kein
16	M	2009		kein
79	M	2009	Normalstatio	13-1
23	M	2009	Normalstatio	13-1
57	F	2009	Normalstatio	13-1
22	M	2009	Normalstatio	13-1
27	M	2009	Normalstatio	13-1
55	M	2009	Normalstatio	kein
66	M	2009		kein
49	M	2009	Normalstatio	9-

25	M	2009		kein
48	M	2009		3-
39	M	2009		3-
19	F	2009	Normalstatio	13-1
83	F	2009	Normalstatio	13-1
97	M	2009	Normalstatio	kein
47	M	2009	Normalstatio	kein
16	M	2009	nach Hau	9-
56	M	2009		3-
74	F	2009	Normalstatio	kein
28	M	2009	IM	kein
59	M	2009	IM	13-1
24	M	2009		kein
57	M	2009	nach Hau	kein
36	M	2009		kein
50	F	2009	nach Hau	13-1
58	M	2009	IM	kein
39	M	2009		kein
48	M	2009		13-1
37	M	2009	Normalstatio	13-1
17	M	2009	nach Hau	13-1
47	M	2009	Normalstatio	kein
47	M	2009	nach Hau	13-1
44	M	2009		13-1
16	M	2009	Normalstatio	kein
68	M	2009		13-1
35	M	2009	nach Hau	3-
65	M	2009		kein
55	M	2009		13-1
25	F	2009	IM	kein
24	M	2009	Normalstatio	13-1
51	M	2009	Normalstatio	13-1
21	M	2009	Normalstatio	13-1
57	M	2009	Normalstatio	13-1
52	M	2009		13-1
67	M	2009		13-1
21	M	2009	Normalstatio	kein
83	M	2009		3-
61	F	2009		kein
58	M	2009		13-1
34	M	2009	Normalstatio	kein
37	M	2009	Normalstatio	13-1
23	M	2009	nach Hau	kein
36	M	2009	Normalstatio	kein
35	M	2009		13-1
16	M	2009		13-1

47	M	2009		13-1
55	F	2009		3-
37	M	2009		3-
35	M	2009		kein
49	M	2009		13-1
38	M	2009	IM	kein
58	M	2009	Normalstatio	13-1
64	M	2009	Normalstatio	kein
75	F	2009		9-
20	M	2009		3-
78	M	2009		13-1
21	M	2009		kein
66	F	2009		13-1
25	F	2009	Normalstatio	13-1
22	M	2009		kein
45	M	2009		13-1
19	F	2009		kein
19	M	2009	Normalstatio	13-1
26	F	2009		kein
25	M	2009		13-1
61	M	2009		13-1
22	M	2009	Normalstatio	kein
46	M	2009	IM	kein
82	M	2009		13-1
40	F	2009	Normalstatio	13-1
16	M	2009	IM	13-1
45	F	2009	Normalstatio	13-1
38	F	2009	IM	13-1
17	M	2009	Normalstatio	13-1
63	M	2009		kein
24	M	2009	Normalstatio	kein
22	M	2009		9-
16	F	2009	Normalstatio	13-1
76	M	2009	IM	13-1
21	M	2009		kein
23	M	2009		13-1
17	M	2009	Normalstatio	13-1
25	M	2009		13-1
51	M	2009		13-1
19	M	2009		9-
27	M	2009		kein
30	M	2009		3-
47	M	2009		13-1
28	M	2009	Normalstatio	kein
20	M	2009	Normalstatio	13-1
50	F	2009		kein

47	F	2009	Normalstatio	13-1
32	M	2009		9-
25	M	2009	nach Hau	13-1
54	M	2009	IM	13-1
31	M	2009	IM	kein
23	M	2009		13-1
38	M	2009	IM	13-1
52	M	2009	Normalstatio	kein
49	F	2009	Normalstatio	kein
58	M	2009		kein
55	M	2009		3-
48	F	2009	Normalstatio	13-1
63	M	2009		kein
33	M	2009	Normalstatio	13-1
60	M	2009		kein
26	M	2009		kein
18	M	2009		3-
52	M	2009		13-1
16	F	2009	Normalstatio	13-1
23	M	2009		kein
40	M	2009		kein
53	M	2009		kein
50	M	2009		kein
27	M	2009	Normalstatio	13-1
33	M	2009	nach Hau	kein
16	M	2009		kein
32	M	2009	nach Hau	kein
71	M	2009		13-1
49	M	2009	IM	13-1
19	M	2009	IM	13-1
26	M	2009	Normalstatio	kein
32	F	2009		kein
37	F	2009	nach Hau	13-1
33	M	2009	Normalstatio	kein
16	M	2009		kein
60	M	2009		3-
62	M	2009		9-
70	M	2009		kein
50	M	2009		kein
22	F	2009		13-1
20	M	2009	Normalstatio	3-
53	F	2009		kein
19	F	2009	Normalstatio	kein
45	F	2009	Normalstatio	13-1
39	M	2009	IM	13-1
59	F	2009	Normalstatio	13-1

46	F	2009	IM	kein
21	M	2009	Normalstatio	13-1
31	M	2009		9-
64	M	2009	Normalstatio	13-1
84	M	2009		13-1
49	M	2009		13-1
40	M	2009	nach Hau	kein
61	M	2009	IM	13-1
87	F	2009		13-1
84	F	2009	IM	13-1
23	M	2009		3-
53	M	2009		kein
53	F	2009		13-1
18	M	2009		13-1
33	M	2009		kein
21	M	2009		13-1
26	M	2009	Normalstatio	kein
17	M	2009		3-
51	M	2009		13-1
65	M	2009		13-1
24	M	2009	IM	13-1
23	M	2009		kein
56	F	2009	Normalstatio	13-1
47	M	2009	Normalstatio	13-1
76	M	2009		13-1
37	M	2009	IM	kein
70	M	2009		kein
38	M	2009		kein
33	M	2009		kein
29	F	2009	IM	13-1
18	F	2009	Normalstatio	kein
49	M	2009		kein
22	M	2009		9-
78	M	2009	Normalstatio	13-1
38	M	2009	nach Hau	kein
64	M	2009		13-1
90	M	2009		13-1
51	M	2009	Normalstatio	kein
28	M	2009	Normalstatio	13-1
17	M	2009	Normalstatio	13-1
52	M	2009		kein
38	M	2009		kein
23	M	2009	Normalstatio	13-1
18	M	2009		3-
18	F	2009		3-
52	F	2009	IM	13-1

18	M	2009		9-
26	M	2009	Normalstatio	13-1
78	M	2009		13-1
64	M	2009		3-
47	M	2009		kein
19	M	2009	Normalstatio	3-
35	F	2009	Normalstatio	13-1
69	M	2009		13-1
44	M	2009		13-1
48	M	2009	Normalstatio	13-1
52	M	2009	Normalstatio	13-1
45	M	2009		13-1
24	M	2009	nach Hau	13-1
16	M	2009		3-
24	F	2009	Normalstatio	kein
38	F	2009	nach Hau	kein
44	M	2009		3-
49	M	2009		
47	F	2009		kein
58	M	2009	IM	13-1
43	M	2009		kein
39	M	2009		13-1
70	M	2009		kein
46	M	2009		3-
91	F	2009	IM	13-1
59	F	2009	IM	13-1
50	M	2009		13-1
20	F	2009		kein
80	M	2009	Normalstatio	kein
24	F	2009		9-
21	M	2009	IM	kein
18	M	2009		13-1
55	M	2009		kein
45	M	2009		kein
66	M	2009	IM	kein
25	M	2009	Normalstatio	kein
18	M	2009		kein
60	M	2009	nach Hau	kein
54	M	2009	Normalstatio	kein
41	M	2009	Normalstatio	kein
22	F	2009	Normalstatio	kein
34	M	2009	Normalstatio	13-1
39	M	2009		kein
24	M	2009		3-
48	M	2009		kein
22	M	2009		3-

25	M	2009		kein
30	M	2009		kein
91	M	2009		kein
78	M	2009		kein
71	F	2009	nach Hau	13-1
38	M	2009		kein
61	F	2009	Normalstatio	kein
66	F	2009		3-
27	M	2009		3-
64	F	2009		3-
71	M	2009	IM	9-
35	M	2009	Normalstatio	13-1
72	M	2009		13-1
75	M	2009		13-1
53	M	2009	Normalstatio	13-1
73	M	2009		9-
24	M	2009		13-1
21	M	2009	IM	13-1
17	M	2009		13-1
66	M	2009	Normalstatio	13-1
59	M	2009	Normalstatio	9-
36	F	2009		13-1
46	F	2009	nach Hau	9-
17	M	2009		9-
40	M	2009		3-
63	M	2009	Normalstatio	kein
47	M	2009		13-1
57	M	2009	Normalstatio	kein
45	M	2009	Normalstatio	13-1
83	F	2009		3-
42	M	2009		kein
53	M	2009		3-
66	M	2009		kein
87	M	2009	IM	13-1
77	M	2009	Normalstatio	kein
71	M	2009		kein
68	M	2009		9-
34	M	2009	Normalstatio	13-1
73	F	2009	IM	13-1
51	M	2009		kein
17	M	2009		13-1
18	M	2009		3-
54	M	2009		3-
68	F	2009		13-1
47	M	2009		3-
31	M	2009		13-1

46	M	2009		3-
73	M	2009		9-
64	F	2009	Normalstatio	kein
55	M	2009		kein
85	F	2009	IM	13-1
57	F	2009	IM	kein
66	F	2009		9-
28	M	2009		kein
58	M	2009		9-
53	M	2009	IM	13-1
70	M	2009		13-1
42	M	2009	Normalstatio	13-1
88	F	2009		3-
35	M	2009	Normalstatio	13-1
31	M	2009	nach Hau	kein
29	M	2009		kein
45	M	2009		13-1
77	M	2009	Normalstatio	13-1
53	M	2009		kein
16	M	2009		13-1
42	M	2009		9-
58	M	2009	IM	13-1
38	F	2009	Normalstatio	13-1
45	M	2009	Normalstatio	13-1
65	F	2009		13-1
47	M	2009	Normalstatio	13-1
68	M	2009		
24	F	2009		3-
65	M	2009		13-1
37	M	2009		3-
48	M	2009		13-1
48	F	2009	Normalstatio	kein
33	M	2009		3-
53	M	2009		9-
17	M	2009		13-1
65	M	2009		3-
75	M	2009		3-
23	M	2009		13-1
47	M	2009		3-
67	M	2009		13-1
83	F	2009	Normalstatio	13-1
26	M	2009	Normalstatio	13-1
33	M	2009	nach Hau	13-1
18	M	2009		13-1
43	M	2009	Normalstatio	kein
23	M	2009		13-1

55	M	2009		13-1
64	M	2009		3-
52	M	2009	IM	13-1
51	F	2009	Normalstatio	kein
41	M	2009	nach Hau	13-1
80	F	2009		13-1
56	M	2009	Normalstatio	13-1
42	M	2009		13-1
35	M	2009		13-1
29	M	2009	Normalstatio	kein
31	M	2009		3-
47	M	2009		kein
88	M	2009		kein
57	M	2009	IM	9-
28	M	2009		kein
70	M	2009		9-
18	M	2009		3-
40	M	2009		9-
37	M	2009	Normalstatio	kein
15	M	2009	IM	kein
59	M	2009		9-
44	F	2009	Normalstatio	kein
16	F	2009	Normalstatio	13-1
26	M	2009		13-1
77	M	2009		13-1
24	M	2009	Normalstatio	kein
64	M	2009		kein
33	M	2009		13-1
66	M	2009		kein
48	F	2009	Normalstatio	3-
25	F	2009		3-
48	M	2009		kein
51	M	2009		9-
31	F	2009		3-
47	M	2009		kein
80	F	2009		9-
54	M	2009		9-
83	M	2009	IM	13-1
34	F	2009	Normalstatio	13-1
24	M	2009		13-1
70	F	2009		13-1
59	M	2009	Normalstatio	13-1
15	M	2009		3-
44	M	2009	Normalstatio	13-1
82	F	2009	nach Hau	kein
61	F	2009	IM	13-1

69	M	2009		13-1
24	M	2009		3-
33	M	2009		9-
17	M	2009		13-1
19	M	2009	IM	kein
58	F	2009		kein
19	M	2009		kein
45	M	2009	IM	9-
33	F	2009	Normalstatio	13-1
33	M	2009		3-
66	M	2009	Normalstatio	kein
56	M	2009		3-
20	M	2009		13-1
28	F	2009	Normalstatio	13-1
37	M	2009	nach Hau	13-1
19	M	2009	Normalstatio	13-1
48	F	2009	nach Hau	13-1
56	F	2009	nach Hau	kein
37	M	2009		kein
28	M	2009	Normalstatio	13-1
73	M	2009		kein
59	M	2009		3-
16	M	2009	IM	13-1
49	M	2009		kein
56	F	2009		kein
26	F	2009	Normalstatio	kein
29	M	2009		kein
67	M	2009		kein
44	M	2009		kein
59	M	2009		kein
44	M	2009		kein
30	F	2009		13-1
18	M	2009	Normalstatio	13-1
26	M	2009		13-1
23	M	2009		13-1
45	M	2009	nach Hau	kein
66	M	2009		13-1
79	M	2009	IM	13-1
29	M	2009	Normalstatio	13-1
81	F	2009	IM	13-1
18	F	2009	nach Hau	kein
31	M	2009		kein
33	M	2009		kein
62	M	2009	Normalstatio	13-1
73	F	2009	IM	13-1
67	M	2009		kein

60	M	2009		3-
61	M	2009		3-
73	M	2009		kein
21	F	2009	nach Hau	13-1
55	M	2009		13-1
51	F	2009	IM	13-1
28	M	2009	Normalstatio	13-1
53	F	2009	IM	13-1
50	M	2009		13-1
60	M	2009	Normalstatio	kein
38	M	2009		13-1
19	F	2009	Normalstatio	kein
85	M	2009		kein
54	F	2009		13-1
28	M	2009	Normalstatio	13-1
61	F	2009		kein
70	M	2009	nach Hau	13-1
82	F	2009		kein
43	M	2009		13-1
22	M	2009		13-1
16	M	2009		13-1
66	M	2009		kein
34	M	2009	Normalstatio	13-1
61	M	2009		kein
43	M	2009	Normalstatio	kein
48	M	2009		kein
50	M	2009	Normalstatio	13-1
42	M	2009		kein
62	F	2009	Normalstatio	kein
28	F	2009	Normalstatio	13-1
17	M	2009		kein
64	M	2009	Normalstatio	kein
24	M	2009		3-
17	F	2009	Normalstatio	13-1
69	M	2009		13-1
45	M	2009	Normalstatio	13-1
77	M	2009	IM	kein
79	F	2009	IM	13-1
25	M	2009		13-1
72	M	2009		13-1
46	M	2009		kein
41	F	2009	Normalstatio	13-1
79	F	2009	Normalstatio	13-1
24	M	2009		3-
85	F	2009		kein
66	M	2009	Normalstatio	13-1

60	M	2009		13-1
18	F	2009	nach Hau	kein
45	M	2009	Normalstatio	13-1
19	M	2009	Normalstatio	9-
62	F	2009	Normalstatio	kein
81	F	2009		3-
67	M	2009		13-1
89	M	2009	Normalstatio	9-
23	F	2009	nach Hau	13-1
61	M	2009	Normalstatio	kein
17	F	2009	Normalstatio	13-1
37	M	2009		kein
59	M	2009		kein
78	M	2009	IM	9-
38	M	2009		3-
39	M	2009	Normalstatio	13-1
83	M	2009	Normalstatio	kein
83	F	2009		kein
84	F	2009	Normalstatio	13-1
52	F	2009		9-
56	F	2009	Normalstatio	kein
53	M	2009		3-
29	M	2009	IM	13-1
25	M	2009		kein
31	F	2009	Normalstatio	kein
40	M	2009	Normalstatio	13-1
92	M	2009	Normalstatio	13-1
19	M	2009	Normalstatio	9-
82	M	2009		3-
27	M	2009		13-1
69	M	2009		3-
19	M	2009		kein
16	M	2009	Normalstatio	kein
34	M	2009	nach Hau	13-1
32	M	2009	nach Hau	13-1
40	M	2009		13-1
64	F	2009	Normalstatio	13-1
19	M	2009		3-
48	M	2009	nach Hau	kein
96	M	2009	Normalstatio	13-1
30	M	2009		kein
32	M	2009	Normalstatio	kein
44	F	2009	IM	3-
48	F	2009	Normalstatio	13-1
21	F	2009	nach Hau	kein
17	M	2009	nach Hau	13-1

88	F	2009	IM	13-1
16	M	2009	Normalstatio	kein
30	M	2009		3-
19	M	2009	Normalstatio	13-1
18	M	2009		3-
28	M	2009	Normalstatio	13-1
49	M	2009		13-1
36	M	2009	Normalstatio	kein
32	F	2009		9-
19	F	2009		3-
21	M	2009		13-1
41	F	2009	Normalstatio	13-1
62	M	2009		3-
53	F	2009		kein
11	M	2009	Normalstatio	3-
17	M	2009		3-
34	F	2009		9-
26	M	2009		kein
76	M	2010	Normalstatio	13-1
39	M	2010	IM	kein
41	F	2010		kein
48	M	2010	IM	9-
43	F	2010		13-1
71	M	2010	Normalstatio	kein
27	M	2010	Normalstatio	kein
29	F	2010	Normalstatio	kein
10	F	2010	Normalstatio	kein
31	F	2010	nach Hau	3-
71	M	2010		3-
60	M	2010	Normalstatio	kein
41	M	2010	Normalstatio	13-1
59	F	2010	Normalstatio	kein
26	M	2010		13-1
33	M	2010		9-
38	M	2010	nach Hau	13-1
16	M	2010		9-
86	F	2010	nach Hau	13-1
51	M	2010		13-1
68	M	2010		13-1
22	M	2010	Normalstatio	13-1
66	M	2010		13-1
62	F	2010	Normalstatio	kein
21	F	2010		3-
41	M	2010		kein
16	M	2010		13-1
28	M	2010	Normalstatio	13-1

27	M	2010		9-
33	M	2010		kein
30	M	2010		kein
16	M	2010		3-
64	M	2010		9-
24	M	2010	nach Hau	13-1
43	F	2010	Normalstatio	13-1
67	M	2010	IM	13-1
23	M	2010	nach Hau	kein
53	F	2010		13-1
27	F	2010		13-1
41	M	2010	IM	13-1
19	M	2010	Normalstatio	13-1
85	F	2010	Normalstatio	9-
39	M	2010	Normalstatio	kein
38	M	2010		3-
42	M	2010		kein
28	M	2010	Normalstatio	13-1
32	M	2010	IM	kein
16	M	2010	Normalstatio	13-1
17	M	2010		13-1
87	M	2010		13-1
51	M	2010		13-1
66	M	2010	Normalstatio	13-1
20	F	2010	nach Hau	13-1
32	M	2010	Normalstatio	13-1
16	F	2010	IM	13-1
33	F	2010		3-
30	M	2010	Normalstatio	kein
72	M	2010		3-
32	F	2010	Normalstatio	3-
36	M	2010		3-
16	M	2010		3-
61	M	2010		13-1
36	M	2010	IM	kein
31	M	2010		kein
34	M	2010		13-1
25	M	2010	Normalstatio	13-1
34	M	2010	Normalstatio	13-1
18	M	2010		kein
37	F	2010	IM	13-1
63	F	2010		kein
37	F	2010	Normalstatio	13-1
28	F	2010		13-1
54	M	2010	Normalstatio	kein
50	M	2010		13-1

39	M	2010		13-1
67	F	2010	Normalstatio	13-1
72	M	2010		13-1
25	M	2010	Normalstatio	13-1
60	M	2010		3-
36	M	2010		9-
52	F	2010		9-
61	F	2010	Normalstatio	13-1
85	M	2010	nach Hau	kein
81	F	2010	IM	kein
18	M	2010	IM	13-1
69	M	2010	nach Hau	13-1
50	M	2010	Normalstatio	13-1
47	M	2010		13-1
44	M	2010	nach Hau	kein
16	F	2010	nach Hau	kein
42	M	2010	nach Hau	13-1
66	M	2010		3-
35	M	2010	IM	13-1
57	M	2010	Normalstatio	13-1
73	F	2010		13-1
39	M	2010		kein
44	F	2010	Normalstatio	kein
21	M	2010	nach Hau	13-1
83	M	2010	IM	3-
42	M	2010		kein
27	M	2010		kein
56	M	2010		kein
36	F	2010		3-
40	M	2010		kein
17	M	2010		13-1
23	M	2010	Normalstatio	kein
22	M	2010		13-1
18	M	2010		kein
53	M	2010	Normalstatio	13-1
52	F	2010		13-1
38	M	2010		9-
51	M	2010		13-1
21	M	2010		kein
48	M	2010		13-1
45	M	2010	nach Hau	kein
55	F	2010		3-
37	F	2010	Normalstatio	13-1
35	F	2010	nach Hau	13-1
57	M	2010		13-1
51	M	2010		kein

25	M	2010	Normalstatio	13-1
67	M	2010		13-1
58	F	2010	nach Hau	kein
27	F	2010		kein
57	M	2010		13-1
53	M	2010	Normalstatio	kein
66	M	2010	nach Hau	3-
61	F	2010		3-
45	M	2010		3-
31	M	2010	nach Hau	13-1
66	F	2010		3-
59	M	2010	Normalstatio	13-1
65	M	2010		13-1
65	M	2010		13-1
51	M	2010	Normalstatio	13-1
35	M	2010	Normalstatio	13-1
83	F	2010		kein
32	M	2010	Normalstatio	13-1
60	M	2010		13-1
46	F	2010		kein
48	M	2010		kein
37	F	2010		13-1
74	M	2010	Normalstatio	kein
19	M	2010		3-
44	M	2010	Normalstatio	kein
65	M	2010		kein
70	F	2010		kein
45	M	2010		kein
56	F	2010	Normalstatio	13-1
63	F	2010	IM	13-1
63	M	2010	Normalstatio	13-1
68	M	2010		3-
43	F	2010	nach Hau	kein
43	M	2010		kein
22	M	2010		kein
50	M	2010	Normalstatio	13-1
46	M	2010		3-
42	M	2010		kein
82	F	2010	Normalstatio	13-1
57	M	2010	Normalstatio	13-1
56	M	2010		3-
26	M	2010	Normalstatio	kein
49	M	2010		9-
53	M	2010		3-
50	M	2010		kein
38	M	2010	IM	3-

52	M	2010	Normalstatio	kein
41	F	2010	Normalstatio	9-
69	M	2010		3-
40	M	2010		13-1
42	M	2010		9-
43	M	2010	Normalstatio	3-
45	M	2010		9-
23	M	2010		13-1
33	F	2010	Normalstatio	13-1
26	M	2010		kein
75	F	2010		
59	M	2010		kein
22	F	2010	Normalstatio	kein
43	F	2010	nach Hau	13-1
26	F	2010	IM	13-1
38	M	2010		9-
22	F	2010		3-
55	M	2010	Normalstatio	13-1
28	F	2010	IM	13-1
20	F	2010	Normalstatio	13-1
41	M	2010	Normalstatio	13-1
21	F	2010		13-1
29	M	2010		kein
42	M	2010	Normalstatio	kein
30	F	2010	Normalstatio	13-1
21	M	2010		kein
55	M	2010	nach Hau	kein
59	M	2010	IM	kein
54	F	2010	Normalstatio	13-1
32	M	2010		kein
40	M	2010		kein
45	M	2010		kein
41	M	2010		kein
37	M	2010	nach Hau	kein
32	M	2010	Normalstatio	13-1
33	M	2010		13-1
23	M	2010		kein
50	M	2010		kein
33	M	2010		13-1
70	F	2010	IM	13-1
24	M	2010		3-
55	M	2010		9-
73	M	2010	Normalstatio	13-1
61	M	2010	IM	13-1
29	M	2010		13-1
57	M	2010		13-1

81	F	2010		kein
38	F	2010	nach Hau	kein
24	F	2010	nach Hau	kein
55	F	2010		13-1
62	M	2010		kein
36	M	2010	nach Hau	kein
68	M	2010		3-
75	M	2010		9-
44	M	2010	IM	13-1
78	M	2010		3-
46	M	2010	Normalstatio	kein
22	M	2010		13-1
45	F	2010		13-1
42	F	2010	Normalstatio	13-1
83	M	2010		9-
47	M	2010	Normalstatio	13-1
92	F	2010		3-
61	M	2010		3-
44	M	2010		13-1
64	M	2010	Normalstatio	13-1
64	M	2010		13-1
86	M	2010		13-1
46	F	2010	Normalstatio	13-1
34	M	2010		kein
56	M	2010	IM	13-1
54	M	2010	Normalstatio	13-1
50	M	2010	Normalstatio	13-1
51	M	2010	Normalstatio	kein
18	M	2010		13-1
44	M	2010	Normalstatio	kein
61	M	2010		13-1
22	M	2010		kein
41	M	2010		kein
29	M	2010		9-
30	F	2010	IM	13-1
62	F	2010	nach Hau	kein
46	F	2010		13-1
21	M	2010		13-1
26	M	2010		kein
43	M	2010	Normalstatio	kein
25	F	2010		3-
22	M	2010	Normalstatio	13-1
16	M	2010		13-1
46	F	2010	Normalstatio	kein
72	M	2010		13-1
32	F	2010		13-1

37	M	2010		13-1
37	M	2010		kein
46	M	2010		3-
79	M	2010		13-1
28	F	2010	nach Hau	kein
32	F	2010		13-1
55	F	2010		kein
31	F	2010		kein
40	M	2010		13-1
46	M	2010	Normalstatio	kein
48	M	2010		13-1
22	M	2010		3-
70	F	2010		9-
21	F	2010	Normalstatio	13-1
66	M	2010		13-1
52	M	2010		kein
73	M	2010		kein
26	M	2010	Normalstatio	13-1
29	M	2010	nach Hau	13-1
27	M	2010	IM	kein
36	M	2010		13-1
29	M	2010		9-
19	M	2010		9-
44	F	2010		kein
79	M	2010	Normalstatio	13-1
32	M	2010		3-
63	M	2010		13-1
31	M	2010	nach Hau	kein
59	M	2010		9-
77	M	2010		3-
43	M	2011		13-1
67	M	2011		3-
64	F	2011		13-1
85	M	2011		3-

Befund_Schädel_Rx
bland

bland
bland
bland
Subduralhämatom
Subduralhämatom
bland
bland
bland
bland
bland
bland
bland
Kombination SDH+EDH
bland
bland
bland
bland
bland
Intrazerebrale Blutung
bland
bland
bland
Kombination SDH+EDH

bland
bland
Fort I und II
bland
blan
blan
blan
blan
blan
blan
Subduralhämato
Schödelbasisfraktu
blan
blan
Subarachnoidalblutun
blan
blan

blan
Epiduralhämatom + Felsenbeinfraktu
Subarachnoidalblutung + multiple Fraktur
blan
Kombination EDH+SA
blan
blan
Fraktur Os nasal
blan
blan
blan
blan
blan
blan

blan
Subarachnoidalblutun
blan

Nasenbeinfrakt
Subarachnoidalblutun

Subarachnoidalblutun
Intrazerebrale Blutu
Subarachnoidalblutung, intrazerebrale Blutung, Felsenbeinfrakt
blan
blan
blan
blan
SAB + EDH + SD
blan

Mandibulafrakt
blan
Subduralhämatom + Fraktu
blan
blan
Subarachnoidalblutun
blan
blan
blan
blan

Subarachnoidalblutun
Subduralhämatom + Frakture
blan

blan
blan
blan
Subduralhämato
blan
blan
blan
Subduralhämato
blan

blan
blan
blan
Subduralhämato
blan
Subduralhämato
Kalottenfraktu
blan
blan

blan
blan
blan
blan
blan
blan
blan
blan
blan
blan
blan

blan
blan
blan
Subduralhämatom + Kontusionsblutun
blan
blan

blan
blan
Blow-Out-Frakt

blan

blan

blan
blan
blan

blan
Epiduralhämatom + Frakture
blan
blan
blan
blan
Kombination SDH+ I
Subarachnoidalblutun
Epiduralhämato
blan
blan
blan
blan
blan
blan
blan
blan
blan
Intrazerebrale Blutu
blan
Kombination SDH+SA
blan
blan
blan
blan
Orbitawandfraktu
SDH + EDH + SAB + Fraktu
blan
blan
SDH + Fraktu
blan
blan
blan
blan

blan
blan
blan
blan
Epiduralhämato

blan
Fraktu
Fraktu
blan
Suturensprengu
blan
blan
blan
blan
Schädelbasisfraktu
blan

blan
blan
blan
blan

blan
Kombination EDH+IC

blan
blan
blan
blan
blan
blan
blan
blan
blan
blan
blan
blan

Subduralhämato
blan
blan
blan
Subduralhämato
blan
blan
blan
blan
Epiduralhämatom + Schädelkalottenfrakt
blan
blan
blan

blan
blan
blan
Nasenbeinfrakt
blan
blan
Subarachnoidalblutun
blan
blan
Gesichtsfraktu
blan
blan
Subarachnoidalblutun
blan
blan
Subduralhämato
blan
blan

Kombination SDH+ I

Gesichtsfrakture
blan
blan
blan
Subarachnoidalblutun
Gesichtsfrakture
blan
blan
blan

blan

Fraktur Occipita

blan
blan
blan
blan
Kombination SDH+SA
blan
blan

blan

blan
blan
blan
blan
Kombination SDH+SA
Subduralhämato
blan
blan
Kombination SDH+SA
Fraktu
blan
blan
blan
blan
blan
extracranielle Blutu

Epiduralhämato
Epiduralhämato
blan
blan
blan
Kombination EDH+SA
Subarachnoidalblutun
Kombination SDH+SA

blan
Epiduralhämato
blan
blan
blan
blan
Epiduralhämato
blan
Kontusionsblutung + Kalottenfraktu
blan
blan
blan
Fraktu
blan
blan
blan
blan
Subduralhämato
blan
blan

blan

blan

blan

blan

blan

blan

Nasenbeinfrakt

blan

Fraktu

blan

Kombination SDH+SAB+ I

blan

blan

blan

Kalottenfraktu

blan

Nasenbeinfrakt

Kombination SDH+EDH + ICB + SA

blan

Kalottenfraktu

blan

Kombination EDH+SA

blan

blan

blan

blan

blan

blan

blan

blan

blan

blan

Kombination SDH+SA

Epiduralhämato

Fraktu

blan

blan

Kombination SAB+ICB + Frak

Fraktu

Kombination SDH+SA

blan

blan

Alveolarkammfraktu

Gesischtsfrakt
blan
Kombination SAB+IC
blan
blan
blan

blan
blan
blan
blan
blan
blan
Kombination SDH+SA
Kombination SDH+SA
blan
blan
blan
Fraktu
Kombination SDH+SA
blan

Fraktu
Intrazerebrale Blutu
blan
blan
blan
Kombination SDH+SA

blan
blan
blan
Epiduralhämato
Unterkieferfrakt
blan
blan
blan
Intrazerebrale Blutu

blan
Mandibularfraktu
blan

Epiduralhämato
blan
blan
multiple Fraktur

Fraktu
blan
Intrazerebrale Blutu
blan
blan

Kombination SDH+SA
blan
blan
Fraktu
blan
Fraktu
blan
Intrazerebrale Blutu
Subarachnoidalblutun
blan
blan
blan

Kombination SDH+SA
blan
Nasenbeinfrakt
blan
Subarachnoidalblutun
Epiduralhämato
blan
Intrazerebrale Blutu
Fraktu
Subarachnoidalblutun
Kombination SDH+SA

Fraktu
blan
blan
Epiduralhämato
Kombination SDH+SA
blan
blan
blan
blan

Fraktu
Kombination SDH+SA
Kontusionsblutun
blan
blan
Kontusionsblutun
blan
blan
blan
blan
Le Fort I und
blan
blan
blan
Intrazerebrale Blutu
blan
Subduralhämato
Kombination SDH+SA
Subduralhämato
Kontusionsblutun
blan
Kombination SDH+SAB+IC
blan
blan
Kombination SDH+SA
blan
blan
blan
Kombination SDH+SA
Kombination SDH+SA
blan
Kombination SDH+ I

blan
Fraktu
blan
blan
blan
blan

Kombination SAB+IC
blan
Fraktu
Epiduralhämato
blan
Sinus maxillaris Frakt

multiple Fraktur

Kombination SAB+IC
Subduralhämato
blan
blan
Kombination SDH+SAB + IC
blan
Subduralhämato
Kombination SDH+ I
Subduralhämato
Kombination SDH+SA
blan
Epiduralhämato
blan
Subduralhämato
blan
blan
blan
blan
blan
blan
blan
blan
blan
blan
blan
blan
blan
blan
blan
Subduralhämato
blan
Subduralhämato
Kombination EDH+SA
blan
blan
blan
blan
blan
Subarachnoidalblutun
blan
blan
blan
blan
Kombination SAB+IC

blan
Kombination SDH+ I
Kombination SDH+ I
blan
Subduralhämato
blan
blan
blan
Fraktu
blan
blan
blan
blan

blan
blan
blan
blan
blan
blan
blan

blan
blan
blan
blan
blan
blan
Intrazerebrale Blutu
blan
blan
blan
blan
blan
blan
Subarachnoidalblutun
blan
Subduralhämato
blan
Kombination SDH+SA
blan
blan
blan
blan
Intrazerebrale Blutu
blan

Epiduralhämato
Kombination SDH+SA
Subarachnoidalblutun
blan
Intrazerebrale Blutu
blan
blan
blan
Kombination SDH+SA
blan
blan
blan
blan
blan
blan
blan
blan
blan
blan
Intrazerebrale Blutu
blan
blan
blan
blan
blan
blan
blan
blan
Subduralhämato
blan

blan
blan
blan
blan
blan
blan
blan
blan
blan
blan
Subduralhämato
blan
blan
blan
blan

blan
blan
blan
blan
blan
blan
blan
blan
blan
blan
Kombination SDH+EDH + IC
blan
blan
blan
blan
blan
Intrazerebrale Blutu
blan
blan
blan
blan
blan
blan
blan
blan
blan
blan
Kombination EDH+IC
Kombination SDH+SA
blan
blan
blan
blan
blan
blan
blan
blan
blan
blan
blan
blan
blan

blan
blan
blan

blan

blan

Kombination SDH+EDH+ICB+SA

blan

blan

Subarachnoidalblutun

blan

Subduralhämato

blan

Kombination EDH+SA

blan

blan

blan

blan

blan

blan

blan

Intrazerebrale Blutu

Epiduralhämato

blan

blan

blan

blan

Intrazerebrale Blutu

blan

blan

blan

blan

blan

blan

Kombination SDH+ED

blan

blan

Subduralhämato

blan

blan

blan

blan

blan

blan

blan

Kombination SDH+SA

Intrazerebrale Blutu

blan

Kombination SDH+SAB+IC

blan

blan

Kombination SDH+ I

blan

Kombination EDH+SA

blan

blan

blan

blan

blan

blan

blan

Kombination SAB+IC

blan

blan

blan

blan

blan

blan

blan

Kombination SDH+SA

Kombination SDH+SA

blan

blan

blan

blan

blan

blan

blan

blan

blan

blan

blan

blan

blan

blan

blan

blan

blan

Kombination EDH+SAB+IC

blan

Subduralhämato

blan
blan
blan
blan
blan
blan
blan
blan
blan
Kombination SDH+SA
Kombination SAB+IC
blan
blan
Kombination SAB+IC
blan
blan
blan
Subarachnoidalblutun
blan
blan
Subduralhämato
blan
blan
blan
Epiduralhämato
blan
blan
blan
blan
Kombination SAB+IC
blan
Intrazerebrale Blutu
blan
Kombination SDH+SAB+IC
blan
blan
Kombination SDH+EDH+SA
blan
Kombination SDH+SAB+IC
blan
blan
blan
Kombination SDH+ I
Kombination SAB+IC
Kombination SDH+SA
blan

Epiduralhämato
blan
blan
blan
Subduralhämato
blan

blan
Intrazerebrale Blutu
blan
blan
blan
Kombination SDH+EDH+SAB+IC
blan

blan
blan
blan
blan
blan
blan
Subarachnoidalblutun
blan
Kombination SDH+ED
Kombination SDH+ED
blan

Subarachnoidalblutun
blan
blan
Kombination SAB+IC
blan
Subduralhämato
blan
blan
Subduralhämato
Kombination SAB+IC
blan
blan
blan
blan
blan
blan
blan
blan
blan

Intrazerebrale Blutu

Kombination EDH+SAB+IC

Intrazerebrale Blutu

blan

blan

blan

blan

blan

Hirnöd

blan

Intrazerebrale Blutu

blan

blan

Subarachnoidalblutun

blan

Subduralhämato

Kombination EDH+IC

Subduralhämato

blan

blan

blan

blan

blan

blan

blan

blan

blan

blan

blan

blan

Kombination SDH+ I

blan

Intrazerebrale Blutu

blan

blan

Subarachnoidalblutun

Kombination SAB+IC

Subarachnoidalblutun

blan

blan

Intrazerebrale Blutu

blan

Subarachnoidalblutun

blan

blan

Kombination EDH+SA

blan
blan
blan
blan
blan

blan
blan

Subarachnoidalblutun
blan
Subarachnoidalblutun
blan
blan
blan
blan
blan
blan
blan
blan
blan
Intrazerebrale Blutu
blan
blan
blan
blan
blan
blan
blan
blan
blan
blan
blan
blan
blan
blan
Intrazerebrale Blutu
Intrazerebrale Blutu
blan
Intrazerebrale Blutu
blan

blan
blan
Kombination SDH+SAB+IC
blan

Kombination SAB+IC
blan
blan
blan
Intrazerebrale Blutu
Subarachnoidalblutun
blan
blan
blan
blan
Subduralhämato
blan
blan
Subduralhämato
blan
blan
blan
blan
Intrazerebrale Blutu
blan
Kombination SDH+EDH+SA
blan
blan
blan
blan
blan
blan
blan
blan
blan
blan
blan
Kombination SDH+ I
blan
blan
blan
blan
blan
blan
Kombination SDH+SA
blan
blan
blan
blan
blan
Kombination SDH+SA

Kombination SDH+ I

blan

blan

Kombination SAB+ICB+ED

blan

Kombination SDH+SA

Epiduralhämato

Kombination SAB+IC

blan

blan

blan

blan

blan

Kombination SAB+IC

Intrazerebrale Blutu

blan

blan

blan

Kombination SDH+ I

blan

Kombination EDH+IC

Subarachnoidalblutun

blan

blan

blan

blan

blan

Kombination SDH+SAB+IC

blan

Kombination SDH+SA

blan

blan

blan

blan

blan

Kombination SAB+IC

blan

blan

blan

blan

blan

blan

blan

blan

Subduralhämato
blan
Subduralhämato
blan
blan
Intrazerebrale Blutu
blan
blan
blan
blan
blan
Subarachnoidalblutun
blan
blan
blan
Intrazerebrale Blutu
Kombination SDH+SAB+IC
blan
blan

blan
Subarachnoidalblutun

blan
blan
blan
Kombination SDH+SAB+IC
blan
blan
blan
Kombination SDH+EDH+SA
Kombination SDH+ED
blan
Kombination SDH+EDH+SA
blan
blan
blan
blan
blan
blan
Intrazerebrale Blutu
blan
Subarachnoidalblutun
blan

Kombination SDH+ I

blan

blan

Epiduralhämato

Intrazerebrale Blutu

blan

Kombination SAB+IC

blan

blan

blan

blan

blan

blan

blan

blan

blan

blan

blan

blan

blan

blan

blan

blan

blan

blan

blan

blan

blan

blan

blan

Subduralhämato

blan

Subduralhämato

Kombination EDH+SA

blan

blan

blan

blan

blan

blan

blan

blan

blan

blan

blan

blan

blan

blan
blan
blan
blan
Subduralhämato
blan
blan
blan
blan
blan
blan
blan
blan
blan
blan
blan
blan
Kombination SDH+SA
Kombination SDH+ED
blan
Kombination SDH+SA
blan
blan
blan
blan
blan
blan
blan
blan
blan
blan
blan
Intrazerebrale Blutu
blan
blan
blan
Epiduralhämato
blan
blan
blan

Kombination SDH+SA
blan
blan
Kombination SAB+IC
blan

blan

Kombination SDH+SA

blan

blan

Subduralhämato

blan

blan

Subduralhämato

blan

Kombination SDH+ I

blan

blan

blan

blan

blan

blan

blan

Kombination SDH+ I

blan

blan

blan

blan

blan

blan

blan

blan

blan

blan

Kombination SDH+SA

blan

Epiduralhämato

blan

blan

blan

blan

Kombination SDH+ED

blan

Intrazerebrale Blutu

blan

blan

blan

Kombination SDH+ED

Kombination SDH+ED

blan

Kombination EDH+IC

blan

Intrazerebrale Blutu

Subarachnoidalblutun

blan

Kombination SDH+ED

Kombination SDH+SA

Kombination SDH+EDH+SA

blan

blan

blan

blan

blan

blan

Kombination SDH+SAB+IC

Subarachnoidalblutun

Intrazerebrale Blutu

blan

blan

blan

blan

blan

blan

blan

blan

blan

blan

blan

blan

blan

blan

blan

blan

blan

blan

blan

blan

blan

blan

Kombination SAB+IC

blan

blan

blan

blan

Intrazerebrale Blutu

blan
blan
blan
blan
blan
blan
Kombination SDH+ I
Kombination SAB+IC
blan
Kombination SDH+SAB+IC
blan
blan
blan
blan
blan
blan
Kombination SDH+SAB+IC
Epiduralhämato
Subduralhämato
blan
blan
Kombination SAB+IC
blan
blan
Subduralhämato
blan
Subarachnoidalblutun
blan
blan
blan
Subarachnoidalblutun
blan
blan
Kombination SAB+IC
blan
blan
blan
Kombination EDH+IC
blan
blan
Kombination SAB+IC
blan
Subduralhämato
blan
Kombination SDH+EDH+SAB+IC
blan

blan

blan

Kombination SDH+SA

blan

blan

blan

blan

blan

Kombination SDH+ I

blan

blan

Kombination SAB+IC

blan

blan

blan

blan

blan

blan

blan

blan

blan

blan

Kombination SDH+ I

blan

blan

Kombination SDH+ I

Epiduralhämato

blan

Kombination SDH+SA

Kombination SDH+SAB+IC

blan

blan

blan

Subduralhämato

Komplikationen_Schaedel	abdominal_injuries	flesh_wound	SHT	AIS_Head
keine	nein	nein	1	1
	nein	ja	2	
keine	nein	ja	1	1
keine	nein	nein	2	
keine	nein	nein	2	
keine	nein	nein	1	1
keine	nein	ja	1	1
keine	nein	nein	2	
keine	nein	ja	1	1
keine	nein	ja	2	
keine	nein	ja	1	1
keine	nein	nein	2	
keine	nein	nein	2	
keine	nein	ja	2	
Mittellinienverlagerung	nein	ja	1	1
keine	nein	ja	1	1
keine	nein	ja	1	1
keine	nein	nein	2	
keine	nein	ja	1	1
keine	nein	nein	2	
keine	nein	ja	1	1
keine	nein	ja	1	1
keine	nein	nein	2	
keine	nein	nein	2	
Mittellinienverlagerung	nein	ja	1	1
			2	
keine	nein	ja	1	1
keine	nein	nein	2	
keine	nein	nein	2	
keine	nein	nein	1	1
keine	nein	ja	1	1
kein	ne		2	
kein	ne		2	
kein	ne	ne	2	
kein	ne	ne	2	
kein	ne		2	
kein	ne	ne	2	
Mittellinienverlagerun	ne		1	1
kein	ne	ne	1	1
kein	ne		2	
kein	ne	ne	1	1
kein	ne	ne	1	1
kein	ne		1	1
kein	ne	ne	2	
	ne	ne	2	

kein	ne	ne	2	
kein	ne	ne	1	1
kein	ne		1	1
kein	ne		2	
kein	ne	ne	1	1
kein	ne	ne	2	
kein	ne		1	1
kein	ne		2	
kein	ne		2	
kein	ne		2	
kein	ne		1	1
kein			1	1
kein	ne		1	1
kein	ne	ne	2	
	ne		1	1
kein	ne		2	
kein	ne	ne	1	1
kein	ne		2	
	ne	ne	2	
kein	ne		2	
kein	ne	ne	1	1
			2	
kein	ne		1	1
kein	ne	ne	1	1
kein	ne	ne	1	1
kein	ne	ne	2	
kein	ne	ne	2	
	ne		2	
kein	ne	ne	1	1
kein	ne		1	1
kein	ne		1	1
	ne	ne	2	
kein	ne	ne	2	
kein	ne		1	1
Mittellinienverlagerun	ne		1	1
kein	ne		1	1
kein	ne		2	
kein	ne		1	1
kein	ne	ne	2	
kein	ne		1	1
kein	ne	ne	2	
kein	ne	ne	2	
	ne	ne	2	
			1	1
kein	ne		1	1
kein	ne		2	

kein	ne		1	1
kein	ne	ne	2	
kein	ne	ne	2	
kein	ne		1	1
kein	ne		1	1
kein	ne	ne	2	
kein	ne		2	
Mittellinienverlagerun	ne		1	1
kein	ne		1	1
			2	
	ne		2	
kein	ne	ne	2	
kein	ne		1	1
kein	ne		2	
Mittellinienverlagerun	ne		1	1
kein	ne	ne	2	
Mittellinienverlagerun	ne	ne	1	1
kein	ne		1	1
kein			2	
kein	ne		1	1
	ne	ne	2	
kein	ne		2	
kein	ne		2	
kein	ne	ne	2	
kein	ne	ne	2	
kein	ne		2	
kein	ne	ne	2	
kein	ne	ne	2	
kein	ne	ne	1	1
kein	ne	ne	1	1
kein	ne		2	
kein	ne	ne	2	
		ne	2	
kein	ne		2	
kein	ne		1	1
kein	ne		1	1
kein	ne		1	1
kein	ne		1	1
kein	ne		2	
	ne	ne	2	
			2	
kein	ne		1	1
kein	ne		1	1
kein	ne	ne	2	
			2	
kein	ne		2	

kein	ne	ne	2	
			1	1
kein	ne		2	
kein	ne	ne	2	
kein	ne	ne	1	1
	ne	ne	2	
			2	
kein			1	1
kein	ne		2	
kein	ne		2	
kein	ne	ne	2	
kein	ne	ne	2	
kein	ne		2	
kein			1	1
kein	ne	ne	1	1
kein	ne		2	
kein	ne		2	
kein	ne	ne	2	
kein	ne	ne	2	
kein	ne	ne	2	
kein	ne		2	
kein	ne	ne	1	1
kein	ne	ne	1	1
kein			2	
kein	ne		1	1
kein	ne		2	
Mittellinienverlagerun	ne	ne	1	1
kein	ne		2	
kein	ne		1	1
kein	ne		1	1
kein	ne	ne	2	
kein	ne		2	
kein	ne		1	1
kein	ne		2	
kein	ne	ne	2	
kein	ne		1	1
kein	ne	ne	2	
kein	ne	ne	2	
kein	ne		2	
kein	ne		2	
	ne		2	
kein	ne		2	
kein			1	1
kein	ne	ne	2	
kein	ne	ne	2	
kein	ne		1	1

kein	ne	ne	2	
kein	ne	ne	1	1
kein			1	1
kein	ne		1	1
kein	ne	ne	1	1
kein	ne	ne	2	
kein	ne		2	
kein			2	
kein	ne		1	1
kein	ne		1	1
kein	ne	ne	2	
	ne	ne	2	
kein	ne		2	
kein			1	1
kein	ne		1	1
kein	ne	ne	2	
	ne		2	
kein	ne	ne	2	
kein	ne		1	1
	ne	ne	2	
kein	ne	ne	2	
kein	ne		2	
kein	ne	ne	2	
	ne		2	
kein	ne		1	1
kein	ne		2	
kein	ne	ne	2	
kein	ne		1	1
kein	ne		2	
kein	ne		1	1
kein	ne		2	
kein	ne		2	
	ne	ne	1	1
kein	ne		1	1
kein	ne	ne	2	
kein	ne		2	
kein	ne		1	1
kein	ne		1	1
kein	ne	ne	2	
kein	ne	ne	2	
kein	ne		1	1
kein	ne		2	
kein	ne		1	1
kein	ne		1	1
kein	ne	ne	2	
kein	ne		2	

kein	ne		2	
kein	ne		1	1
kein	ne		2	
kein	ne		1	1
kein	ne	ne	2	
kein	ne		2	
kein	ne		1	1
kein	ne		2	
kein	ne		1	1
kein	ne		1	1
kein	ne	ne	1	1
kein	ne	ne	2	
kein	ne		1	1
kein	ne	ne	2	
kein	ne	ne	2	
kein	ne		1	1
kein	ne	ne	2	
kein	ne	ne	2	
	ne		2	
Mittellinienverlagerun	ne		1	1
	ne	ne	2	
kein	ne		1	1
kein	ne		1	1
kein	ne		2	
kein	ne	ne	2	
kein	ne		1	1
kein	ne		1	1
kein			2	
kein	ne		2	
kein	ne		1	1
	ne		2	
	ne		1	1
kein	ne	ne	2	
	ne		2	
		ne	2	
kein	ne	ne	2	
	ne		2	
kein	ne		1	1
kein	ne		2	
kein	ne		2	
kein	ne		1	1
kein	ne		1	1
kein	ne		1	1
kein	ne		2	
			2	
kein			2	

kein	ne		1	1
kein			1	1
kein	ne		1	1
kein	ne		1	1
kein	ne		1	1
kein	ne		1	1
kein	ne		2	
kein	ne		1	1
kein	ne		1	1
kein	ne		1	1
kein			1	1
kein	ne	ne	2	
kein			1	1
kein	ne	ne	2	
kein	ne		2	
kein	ne		2	
	ne	ne	2	
kein			1	1
kein	ne		1	1
kein	ne		2	
kein	ne		2	
kein	ne		2	
kein	ne	ne	1	1
kein	ne		1	1
kein			1	1
	ne	ne	2	
kein	ne		1	1
kein	ne	ne	1	1
kein	ne		2	
kein	ne		2	
kein	ne		1	1
kein	ne		2	
kein	ne	ne	1	1
kein	ne	ne	2	
kein	ne	ne	1	1
kein	ne	ne	2	
kein			2	
kein	ne		2	
kein	ne		1	1
kein	ne		1	1
kein			2	
kein			2	
kein	ne		2	
kein	ne		1	1
kein	ne		1	1
kein	ne		2	

kein	ne		2	
kein	ne		2	
kein	ne	ne	2	
kein	ne	ne	2	
kein	ne	ne	2	
kein	ne		2	
kein	ne		2	
kein	ne		2	
kein	ne		1	1
kein			2	
	ne		2	
	ne	ne	2	
Mittellinienverlagerun	ne		1	1
kein	ne	ne	2	
kein		ne	2	
kein	ne		2	
kein	ne		1	1
kein	ne		1	1
kein	ne		1	1
kein	ne		1	1
kein	ne		2	
kein	ne	ne	1	1
kein	ne	ne	2	
kein	ne		1	1
kein	ne		1	1
kein	ne		2	
kein	ne	ne	2	
kein		ne	2	
kein			2	
kein	ne		2	
kein	ne		1	1
kein	ne		2	
kein	ne		2	
kein	ne		2	
Ventrikeleinbruc	ne		1	1
kein	ne	ne	1	1
kein	ne		1	1
kein	ne		1	1
kein	ne		1	1
kein	ne	ne	1	1
kein	ne		1	1
kein	ne	ne	1	1
kein	ne	ne	2	
kein	ne	ne	2	
kein	ne		2	
kein	ne	ne	2	

kein			1	1
kein	ne	ne	2	
	ne		1	1
kein	ne		2	
kein	ne	ne	2	
kein	ne	ne	2	
	ne	ne	2	
	ne		1	1
kein	ne		1	1
kein	ne		1	1
kein	ne	ne	2	
kein	ne		2	
kein	ne	ne	2	
kein	ne		2	
kein	ne	ne	1	1
kein	ne	ne	1	1
kein	ne	ne	2	
kein	ne		2	
kein	ne		1	1
kein	ne		1	1
Mittellinienverlagerung+Ventrikeleinbruc	ne	ne	1	1
kein	ne	ne	2	
	ne	ne	2	
kein	ne		1	1
kein	ne		1	1
kein	ne	ne	2	
kein	ne	ne	2	
kein	ne		1	1
Mittellinienverlagerung+Ventrikeleinbruc	ne		1	1
	ne		2	
	ne		2	
	ne	ne	2	
kein	ne		1	1
kein	ne		2	
kein	ne		2	
kein	ne	ne	2	
kein	ne		2	
kein	ne		2	
kein	ne		2	
kein	ne	ne	2	
kein	ne		1	1
kein	ne	ne	2	
	ne		2	
kein	ne	ne	2	
kein	ne		1	1
kein	ne		1	1

kein	ne		1	1
kein	ne	ne	1	1
kein	ne		2	
kein	ne		1	1
kein	ne		1	1
kein	ne		1	1
kein	ne		1	1
kein	ne		1	1
kein	ne		1	1
kein	ne		2	
			2	
		ne	2	
Mittellinienverlagerun	ne	ne	1	1
kein	ne	ne	2	
kein	ne		2	
kein	ne		1	1
kein	ne		1	1
kein	ne		1	1
kein	ne	ne	2	
kein			1	1
kein			1	1
kein	ne		1	1
kein	ne		2	
kein	ne		1	1
	ne		2	
kein	ne	ne	1	1
kein	ne		1	1
kein	ne		1	1
kein	ne	ne	2	
kein	ne		1	1
kein	ne	ne	1	1
kein	ne		1	1
kein	ne		1	1
kein	ne		1	1
kein	ne	ne	1	1
kein	ne		1	1
	ne	ne	2	
kein	ne		2	
kein	ne		1	1
kein	ne	ne	2	
Mittellinienverlagerun	ne		1	1
kein	ne		1	1
kein	ne		1	1
kein	ne		2	
kein	ne		1	1
kein	ne		2	

kein	ne		1	1
Ventrikeleinbruc	ne		1	1
kein	ne		1	1
kein	ne		1	1
kein			1	1
kein	ne	ne	1	1
kein	ne		1	1
kein	ne	ne	2	
kein	ne		2	
kein	ne	ne	1	1
kein	ne		1	1
kein	ne		2	
kein	ne		2	
kein	ne		2	
Ventrikeleinbruc	ne	ne	1	1
kein	ne		1	1
kein	ne		1	1
Mittellinienverlagerun	ne		1	1
kein	ne	ne	1	1
kein	ne		1	1
kein	ne	ne	2	
kein	ne		1	1
kein	ne		1	1
kein	ne	ne	2	
Ventrikeleinbruc	ne		1	1
kein	ne		1	1
kein	ne		2	
kein	ne		2	
kein	ne		1	1
Mittellinienverlagerung+Ventrikeleinbruc	ne	ne	1	1
kein			1	1
kein	ne	ne	1	1
	ne		2	
kein	ne		2	
kein	ne		2	
kein	ne		1	1
kein	ne	ne	2	
kein	ne	ne	2	
kein	ne	ne	2	
			2	
kein	ne		1	1
kein	ne	ne	2	
kein	ne		1	1
kein	ne		1	1
kein			1	1
kein			1	1

kein	ne		1	1
	ne		2	
kein	ne		1	1
kein	ne		1	1
kein	ne		2	
kein	ne		1	1
kein	ne		2	
kein	ne		2	
kein	ne		1	1
Ventrikeleinbruc		ne	1	1
Mittellinienverlagerun	ne		1	1
kein	ne		1	1
kein	ne	ne	2	
kein	ne	ne	1	1
kein	ne		2	
Ventrikeleinbruc			1	1
kein			2	
kein		ne	1	1
kein	ne	ne	2	
kein	ne	ne	2	
kein		ne	2	
kein	ne	ne	2	
kein	ne		2	
kein	ne		1	1
kein	ne		2	
kein	ne		2	
kein	ne		1	1
kein	ne	ne	2	
kein	ne	ne	2	
kein	ne		2	
kein	ne		1	1
kein	ne		1	1
kein	ne	ne	1	1
Mittellinienverlagerun	ne	ne	2	
kein	ne	ne	1	1
kein	ne	ne	2	
kein	ne		2	
kein	ne	ne	2	
kein	ne		2	
kein	ne	ne	2	
kein	ne		1	1
kein	ne		2	
kein	ne		1	1
kein	ne	ne	2	
kein	ne		2	
kein	ne	ne	1	1

kein	ne		2	
Mittellinienverlagerun	ne	ne	1	1
Ventrikeleinbruc	ne		1	1
kein	ne	ne	2	
kein	ne		1	1
kein	ne		1	1
kein	ne		2	
kein	ne	ne	2	
kein	ne	ne	1	1
kein	ne		2	
kein	ne		2	
kein	ne	ne	2	
kein	ne		1	1
	ne		2	
kein	ne		2	
kein	ne	ne	2	
kein	ne	ne	2	
kein	ne	ne	2	
kein	ne		1	1
kein	ne		2	
kein	ne	ne	2	
	ne	ne	1	1
kein	ne		1	1
kein	ne		1	1
kein	ne		1	1
kein	ne	ne	2	
kein	ne	ne	2	
kein	ne		2	
kein	ne		1	1
kein	ne		2	
kein	ne		1	1
kein	ne	ne	1	1
kein	ne		2	
kein	ne	ne	1	1
kein	ne		1	1
kein	ne		1	1
kein	ne	ne	2	
Mittellinienverlagerun	ne		1	1
kein	ne	ne	2	
kein	ne		1	1
kein		ne	2	
kein	ne		1	1
kein	ne		1	1
kein	ne	ne	2	
kein	ne		1	1
kein	ne		1	1

kein	ne		1	1
kein	ne		1	1
kein	ne		1	1
kein	ne	ne	2	
kein	ne		1	1
kein	ne		2	
kein	ne		1	1
kein	ne		1	1
kein	ne		1	1
kein		ne	2	
kein	ne		2	
kein	ne		2	
kein	ne		1	1
kein	ne		1	1
kein			2	
kein	ne		1	1
kein	ne	ne	2	
kein	ne		2	
kein	ne	ne	2	
kein			1	1
kein	ne		1	1
kein	ne		2	
kein	ne		2	
kein	ne		2	
kein	ne		2	
kein	ne		2	
kein	ne		2	
kein	ne	ne	2	
kein	ne		2	
kein	ne	ne	2	
	ne		1	1
kein	ne		2	
kein	ne	ne	2	
kein	ne		1	1
kein	ne	ne	2	
kein	ne		1	1
kein	ne		1	1
kein		ne	2	
kein	ne		1	1
kein			1	1
kein	ne		2	
kein			1	1
kein	ne		1	1
kein			2	
kein	ne		1	1
kein	ne		2	

kein	ne	ne	1	1
kein	ne		1	1
kein	ne		1	1
kein	ne		1	1
kein	ne		2	
kein	ne	ne	2	
kein	ne	ne	2	
kein	ne	ne	2	
kein	ne	ne	2	
kein	ne		2	
Mittellinienverlagerun	ne	ne	1	1
kein	ne		2	
kein			1	1
kein	ne	ne	1	1
kein	ne		2	
kein	ne	ne	2	
kein			1	1
kein	ne		1	1
kein	ne		2	
kein	ne	ne	2	
kein	ne		2	
kein	ne	ne	2	
kein	ne		2	
kein	ne	ne	2	
kein	ne		2	
kein		ne	2	
kein	ne	ne	2	
kein	ne		1	1
kein	ne		1	1
kein	ne		1	1
kein	ne		2	
kein	ne	ne	2	
kein	ne		1	1
kein	ne		2	
kein	ne	ne	2	
kein	ne		1	1
kein	ne		1	1
kein	ne		2	
kein	ne	ne	2	
kein			1	1
kein			1	1
kein	ne		2	
	ne		2	
kein	ne	ne	2	
kein	ne		2	
kein	ne		1	1

kein	ne		2	
kein	ne		1	1
kein	ne		1	1
kein	ne	ne	2	
kein	ne	ne	1	1
kein	ne		2	
kein	ne		1	1
kein	ne		1	1
kein	ne		1	1
kein	ne		1	1
kein	ne		1	1
kein	ne	ne	2	
kein	ne		1	1
kein	ne		1	1
kein	ne		2	
kein	ne	ne	1	1
kein	ne	ne	2	
Ventrikeleinbruc			1	1
kein	ne		2	
kein	ne		1	1
	ne	ne	2	
kein	ne		2	
kein	ne	ne	1	1
kein	ne	ne	1	1
kein	ne		1	1
kein	ne	ne	2	
	ne		2	
kein	ne		1	1
kein			2	
kein	ne		1	1
kein	ne		2	
kein	ne		2	
Mittellinienverlagerun	ne		1	1
kein	ne	ne	2	
kein	ne		2	
kein	ne		1	1
kein	ne		1	1
kein	ne		1	1
kein	ne		2	
kein	ne		1	1
kein	ne		1	1
kein	ne		2	
kein	ne		2	
kein			1	1
kein			1	1
kein	ne		1	1

kein	ne		1	1
kein	ne		1	1
kein	ne		1	1
Mittellinienverlagerun	ne		1	1
kein	ne	ne	2	
kein	ne		1	1
kein	ne		1	1
kein	ne		2	
kein			1	1
kein	ne		1	1
kein	ne	ne	2	
kein			1	1
kein	ne		2	
kein	ne	ne	1	1
kein	ne	ne	2	
kein	ne	ne	2	
kein	ne		2	
	ne		1	1
kein	ne		2	
kein	ne	ne	2	
kein	ne		2	
kein	ne	ne	2	
	ne		1	1
kein			1	1
kein	ne		1	1
kein	ne		1	1
kein	ne		1	1
kein	ne		2	
kein	ne		2	
kein	ne		2	
kein		ne	2	
kein	ne		1	1
kein	ne	ne	2	
kein	ne		2	
kein	ne		1	1
kein	ne		1	1
kein	ne		2	
kein	ne		2	
kein			2	
	ne	ne	2	
kein	ne	ne	2	
kein	ne		1	1
kein	ne		2	
kein			1	1
kein			2	
kein	ne		1	1

kein	ne		1	1
kein	ne		1	1
kein	ne		2	
kein		ne	2	
kein	ne		1	1
kein	ne	ne	2	
kein	ne	ne	2	
kein			2	
Mittellinienverlagerun			1	1
Ventrikeleinbruc	ne	ne	1	1
kein	ne	ne	1	1
kein	ne		2	
kein	ne		1	1
kein			1	1
kein	ne		2	
kein	ne		1	1
kein	ne		2	
kein	ne	ne	1	1
kein	ne		1	1
kein	ne	ne	2	
kein	ne	ne	1	1
kein	ne		2	
kein	ne		1	1
kein	ne		1	1
kein	ne	ne	1	1
kein	ne		1	1
kein	ne		2	
kein	ne		2	
kein	ne	ne	2	
kein			1	1
kein	ne		2	
kein	ne	ne	1	1
kein	ne	ne	2	
kein	ne		1	1
kein	ne	ne	2	
kein	ne	ne	2	
kein	ne		1	1
kein	ne	ne	2	
kein	ne		1	1
kein	ne		2	
kein			2	
kein			2	
Mittellinienverlagerun	ne	ne	1	1
kein	ne	ne	1	1
kein	ne		1	1
kein	ne		2	

kein	ne		1	1
kein	ne		1	1
kein	ne		2	
kein	ne		2	
kein	ne	ne	2	
kein			1	1
	ne		2	
kein	ne	ne	2	
kein	ne		1	1
kein	ne		2	
kein	ne		1	1
kein	ne		1	1
kein	ne		1	1
kein	ne		1	1
	ne	ne	2	
kein		ne	2	
kein	ne	ne	2	
kein	ne	ne	2	
kein	ne	ne	2	
kein	ne		2	
kein	ne		1	1
kein	ne		1	1
kein	ne	ne	2	
kein	ne		1	1
kein	ne		1	1
kein	ne		2	
			2	
kein			1	1
kein	ne		1	1
kein			1	1
kein	ne	ne	1	1
kein	ne	ne	2	
kein			1	1
kein	ne		1	1
kein	ne		2	
kein	ne		1	1
kein	ne		1	1
kein	ne		1	1
kein	ne		1	1
kein	ne		1	1
kein	ne		1	1
kein	ne		1	1
kein	ne	ne	2	
kein	ne		1	1
kein	ne		2	
kein	ne		2	

kein	ne		1	1
kein	ne		1	1
kein			1	1
kein	ne	ne	2	
Mittellinienverlagerun	ne		1	1
kein	ne		1	1
kein	ne	ne	2	
kein			1	1
kein	ne		2	
kein	ne	ne	2	
kein	ne	ne	1	1
kein	ne		2	
kein	ne		2	
kein	ne		1	1
kein	ne		2	
Mittellinienverlagerun	ne	ne	1	1
kein	ne		1	1
kein	ne		1	1
kein	ne		2	
kein	ne		1	1
kein			1	1
kein		ne	2	
kein	ne		2	
kein			2	
kein	ne		1	1
kein	ne	ne	2	
kein	ne	ne	2	
kein	ne	ne	2	
kein	ne		2	
kein	ne	ne	2	
kein	ne		1	1
kein	ne		2	
kein	ne		1	1
kein	ne		1	1
kein	ne		2	
kein			1	1
kein			1	1
kein	ne		2	
kein	ne		1	1
kein	ne		1	1
kein	ne		1	1
kein	ne		1	1
kein	ne	ne	1	1
kein	ne	ne	2	
kein	ne	ne	2	
kein	ne		1	1

kein	ne	ne	2	
kein	ne		1	1
kein	ne		1	1
kein	ne		1	1
kein	ne	ne	2	
			2	
kein	ne	ne	2	
kein	ne		2	
	ne		2	
kein			1	1
kein	ne		2	
kein	ne		1	1
kein			1	1
kein	ne		2	
kein	ne		1	1
kein	ne	ne	1	1
kein	ne	ne	2	
kein	ne		1	1
kein	ne		2	
kein	ne		1	1
kein	ne		2	
kein	ne	ne	1	1
kein	ne		1	1
kein	ne		2	
kein	ne		2	
kein	ne	ne	2	
kein		ne	2	
kein	ne	ne	2	
kein	ne	ne	2	
kein	ne		2	
kein	ne		2	
kein			2	
kein	ne		1	1
kein	ne		1	1
kein	ne	ne	2	
kein	ne		2	
kein	ne	ne	1	1
kein	ne		1	1
kein	ne		2	
kein	ne		1	1
kein	ne	ne	2	
	ne		2	
kein	ne		2	
kein	ne		1	1
kein			1	1
kein	ne	ne	2	

kein	ne		1	1
kein			1	1
kein	ne		2	
kein	ne	ne	2	
Ventrikeleinbruc	ne	ne	1	1
kein	ne		1	1
kein	ne	ne	2	
kein	ne		1	1
kein	ne		2	
kein	ne		2	
Mittellinienverlagerun	ne	ne	1	1
kein		ne	2	
kein	ne	ne	1	1
kein	ne		1	1
kein	ne	ne	1	1
kein			2	
kein	ne	ne	2	
kein	ne		2	
kein	ne	ne	1	1
kein	ne		1	1
kein	ne	ne	1	1
kein		ne	2	
kein	ne		1	1
kein	ne		1	1
kein	ne		1	1
kein	ne		2	
kein	ne		2	
kein	ne	ne	2	
kein	ne		2	
kein	ne	ne	2	
kein		ne	2	
kein	ne	ne	2	
kein	ne	ne	1	1
kein	ne	ne	2	
kein	ne		1	1
kein	ne		2	
kein	ne		1	1
kein	ne		2	
kein	ne		1	1
kein	ne		1	1
kein	ne	ne	2	
kein	ne		1	1
kein	ne	ne	1	1
kein	ne	ne	1	1
kein	ne		2	
kein	ne		1	1

kein	ne	ne	1	1
kein	ne	ne	2	
kein	ne	ne	1	1
kein	ne	ne	1	1
kein	ne	ne	2	
kein	ne		1	1
Mittellinienverlagerun	ne	ne	1	1
kein	ne	ne	1	1
kein	ne	ne	2	
kein	ne		2	
kein	ne	ne	2	
kein	ne		2	
kein			2	
kein	ne		1	1
Ventrikeleinbruc	ne	ne	1	1
kein	ne	ne	2	
kein		ne	2	
	ne	ne	2	
kein	ne	ne	2	
kein	ne	ne	1	1
kein	ne	ne	2	
kein			1	1
Mittellinienverlagerun	ne	ne	1	1
kein	ne		2	
kein	ne	ne	2	
kein	ne		1	1
kein	ne		1	1
kein	ne		2	
kein	ne	ne	1	1
kein			2	
Ventrikeleinbruc	ne		1	1
kein	ne		1	1
	ne		2	
kein	ne	ne	1	1
kein	ne		2	
kein	ne		2	
kein	ne	ne	1	1
kein	ne	ne	1	1
kein	ne		1	1
kein	ne		1	1
kein	ne		1	1
kein	ne	ne	2	
kein	ne		1	1
kein	ne		1	1
kein	ne	ne	2	
kein	ne		2	

kein	ne		1	1
kein	ne	ne	2	
kein	ne		1	1
kein	ne		1	1
		ne	1	1
kein	ne	ne	1	1
kein	ne		1	1
kein	ne	ne	2	
kein	ne		1	1
kein	ne		1	1
kein	ne		1	1
kein	ne	ne	1	1
kein	ne		2	
kein			2	
kein	ne	ne	1	1
kein	ne		1	1
kein	ne	ne	1	1
kein	ne	ne	2	
kein	ne		2	
	ne	ne	2	
	ne		2	
kein	ne		1	1
kein			2	
	ne	ne	2	
	ne		1	1
kein	ne	ne	2	
kein	ne		2	
kein	ne	ne	2	
Mittellinienverlagerun	ne		1	1
kein	ne	ne	2	
kein	ne		2	
kein	ne		1	1
Mittellinienverlagerun	ne		1	1
kein	ne	ne	1	1
kein	ne	ne	2	
kein	ne		1	1
kein	ne		1	1
kein		ne	2	
kein	ne	ne	1	1
kein	ne		1	1
kein	ne	ne	2	
kein	ne	ne	2	
kein	ne	ne	1	1
kein	ne		1	1
kein			1	1
kein	ne	ne	2	

kein	ne		1	1
kein	ne	ne	2	
kein	ne	ne	2	
Mittellinienverlagerung+Ventrikeleinbruc		ne	1	1
kein	ne		1	1
kein	ne		1	1
kein	ne		1	1
kein			2	
kein	ne	ne	2	
kein	ne	ne	2	
kein	ne		2	
kein	ne	ne	1	1
kein	ne	ne	2	
kein	ne	ne	2	
kein	ne		2	
kein	ne		1	1
kein			2	
kein	ne	ne	2	
kein	ne	ne	2	
kein	ne	ne	2	
kein	ne		1	1
kein	ne		1	1
kein	ne	ne	2	
kein	ne	ne	2	
kein	ne		2	
kein	ne		2	
kein			1	1
kein	ne	ne	2	
kein	ne	ne	2	
Mittellinienverlagerun	ne	ne	2	
kein	ne		1	1
Mittellinienverlagerun	ne		1	1
kein	ne		1	1
kein		ne	2	
kein			1	1
kein	ne		2	
kein	ne		1	1
kein	ne		1	1
kein	ne		2	
kein	ne		2	
kein	ne		2	
kein	ne	ne	2	
kein	ne		1	1
kein	ne		2	
kein	ne		1	1
kein	ne		2	

kein	ne		1	1
kein	ne	ne	1	1
kein	ne		1	1
kein		ne	1	1
Mittellinienverlagerun	ne	ne	1	1
kein	ne		1	1
kein	ne		1	1
kein	ne		1	1
kein	ne		1	1
kein	ne		2	
kein	ne		1	1
kein	ne	ne	2	
kein	ne		2	
kein			1	1
kein	ne		2	
kein	ne	ne	2	
kein	ne	ne	2	
kein	ne		1	1
kein	ne		1	1
kein	ne		2	
kein	ne		1	1
kein	ne	ne	2	
kein	ne		2	
kein	ne	ne	2	
kein	ne		1	1
kein	ne	ne	2	
kein	ne		2	
kein	ne		2	
kein	ne	ne	2	
kein		ne	2	
kein			1	1
kein	ne		2	
kein	ne		1	1
kein	ne	ne	2	
kein	ne		2	
kein	ne		1	1
kein	ne		1	1
kein	ne	ne	2	
kein	ne		2	
kein	ne		1	1
	ne		2	
kein	ne		1	1
kein	ne		1	1
kein	ne	ne	2	
kein	ne		1	1
kein	ne	ne	2	

kein	ne		1	1
kein	ne		1	1
kein	ne	ne	2	
kein	ne		2	
kein	ne		1	1
	ne	ne	2	
kein	ne		1	1
kein	ne	ne	2	
Mittellinienverlagerun	ne		1	1
kein	ne	ne	2	
Mittellinienverlagerun	ne	ne	1	1
kein	ne		1	1
kein	ne		1	1
kein	ne		2	
kein	ne		2	
kein	ne		2	
kein			2	
kein	ne		1	1
kein	ne		1	1
kein	ne	ne	2	
kein	ne	ne	2	
kein			2	
kein	ne	ne	2	
kein	ne		1	1
kein	ne	ne	2	
kein	ne		2	
kein	ne	ne	2	
kein	ne	ne	2	
kein	ne		1	1
Mittellinienverlagerun	ne		1	1
kein	ne		1	1
kein	ne	ne	1	1
kein	ne		2	
kein	ne	ne	2	
kein			2	
kein	ne	ne	2	
kein	ne		1	1
kein	ne	ne	2	
kein	ne		1	1
kein	ne		1	1
kein	ne		2	
kein	ne	ne	1	1
kein	ne		1	1
Mittellinienverlagerun			1	1
kein	ne		2	
kein	ne		2	

kein	ne	ne	2	
kein	ne		1	1
kein			1	1
kein	ne		1	1
kein	ne		1	1
Mittellinienverlagerun	ne		1	1
Mittellinienverlagerung+Ventrikeleinbruc	ne		1	1
kein	ne		1	1
kein	ne		1	1
kein	ne	ne	2	
			2	
kein			2	
kein	ne	ne	2	
kein	ne	ne	2	
kein	ne		1	1
kein	ne		1	1
kein	ne	ne	1	1
kein	ne		1	1
kein	ne		2	
kein	ne	ne	2	
kein	ne		2	
kein	ne		1	1
kein	ne		1	1
kein	ne	ne	2	
	ne	ne	2	
kein	ne		2	
kein	ne		2	
kein			2	
kein	ne		1	1
kein			2	
kein	ne		1	1
kein	ne	ne	2	
kein	ne	ne	2	
kein	ne		2	
kein	ne		1	1
kein	ne	ne	1	1
kein	ne		1	1
kein	ne	ne	2	
kein	ne		2	
kein	ne		1	1
Ventrikeleinbruc	ne		1	1
kein	ne		2	
kein	ne		1	1
kein	ne		1	1
kein	ne		2	
kein	ne		1	1

kein	ne		2	
kein	ne		2	
	ne		2	
kein	ne		1	1
kein			2	
kein	ne		2	
kein	ne		1	1
kein	ne	ne	1	1
kein	ne		1	1
Mittellinienverlagerun	ne	ne	1	1
kein	ne	ne	2	
kein	ne		2	
kein	ne		2	
kein			2	
kein	ne		1	1
kein	ne		1	1
kein	ne		1	1
Mittellinienverlagerun	ne		1	1
Mittellinienverlagerun	ne		1	1
kein	ne		2	
kein			1	1
kein	ne		1	1
kein	ne		1	1
kein	ne	ne	2	
kein	ne		1	1
kein			1	1
kein	ne		1	1
kein	ne	ne	2	
kein	ne	ne	2	
kein	ne		2	
kein	ne		1	1
kein	ne		2	
kein	ne	ne	2	
kein	ne		1	1
kein	ne		1	1
kein	ne	ne	2	
kein	ne		2	
kein	ne	ne	1	1
kein	ne		2	
kein			2	
	ne		1	1
kein	ne		1	1
kein	ne		2	
kein			2	
kein	ne		1	1
kein			2	

kein	ne		2	
kein	ne	ne	2	
kein	ne	ne	1	1
kein	ne		2	
kein	ne	ne	2	
kein	ne		1	1
kein	ne	ne	2	
kein	ne	ne	2	
Mittellinienverlagerun	ne		1	1
kein	ne		2	
kein	ne		2	
kein	ne		1	1
kein	ne		2	
kein	ne		1	1
kein			1	1
kein	ne	ne	2	
kein		ne	2	
kein	ne	ne	1	1
kein			1	1
kein	ne	ne	2	
kein	ne		1	1
kein	ne	ne	2	
Mittellinienverlagerun	ne		1	1
kein	ne		2	
kein	ne		1	1
kein		ne	1	1
kein	ne		1	1
kein	ne		1	1
kein	ne		1	1
Mittellinienverlagerun	ne		1	1
kein			1	1
kein	ne		1	1
kein	ne		1	1
Mittellinienverlagerun	ne		1	1

AIS_Score_Head	AIS_Face	AIS_Score_Face	AIS_Spine	AIS_Score_Spine	AIS_Thorax	AIS_Score_Thorax
1			1	2		
3	1	1				
			1	2		
1	1	1				
3	1	1			1	2
1	1	2				
					1	1
1	1	1				
			1	2		
					1	1
4						
1	1	2				
1	1	1				
	1	1				
3	1	2				
	1	2				
5						
3	1	2				
			1	1		
4						
1					1	3
3	1	2				
3						
	1	3			1	3
	1	2				
			1	2		
4	1	2				
3						
	1	1				
1			1	2		
2						
1	1	1				

3						
3	1	1				
	1	1				
2						
	1	1				
1			1	1		
	1	1				
	1	1			1	1
	1	2				
1	1	1				
1			1	3	1	3
1	1	2				
	1	2				
3	1	1				
	1	1				
2			1	3		
	1	1				
			1	3		
	1	2				
2						
3						
3	1	2				
3			1	2	1	3
			1	1		
					1	1
3						
3					1	3
1						
	1	2				
3					1	1
3	1	1			1	1
2	1	2				
	1	1			1	3
3	1	1				
3					1	1
			1	1		
					1	1
3			1	3	1	3
3					1	5

3	1	1				
					1	2
3	1	1				
1						
			1	1		
	1	1				
5						
1						
					1	1
					1	2
1						
	1	1				
5						
5						
4						
			1	3		
1			1	5		
			1	3		
	1	1				
			1	1		
	1	1				
					1	3
					1	3
1			1	1		
1			1	1		
			1	1		
					1	3
2						
2			1	2	1	2
3					1	3
1	1	2				
			1	3		
					1	1
1						
1	1	2				
	1	2				
	1	1				

2	1	3				
	1	1				
3						
					1	1
	1	1	1	3		
4	1	2	1	2	1	2
					1	1
			1	5		
					1	1
2					1	2
2						
	1	1				
	1	1				
			1	3		
			1	3		
					1	5
2			1	2		
1			1	3	1	3
	1	1			1	2
4			1	2	1	4
	1	1				
4					1	2
3	1	1				
2						
			1	2	1	3
	1	2	1	1		
3	1	2				
					1	1
3	1	1				
			1	3		
			1	2		
					1	2
					1	1
2	1	1				
			1	3		
			1	3	1	2
3						

1	1	2				
3	1	2			1	3
1						
4						
	1	1	1	2	1	3
					1	1
2			1	2	1	1
2	1	1				
			1	1		
			1	1		
1						
1						
	1	1				
			1	2	1	3
2			1	2		
			1	1		
			1	1		
					1	1
					1	1
3	1	1				
1	1	1			1	1
					1	1
1					1	1
					1	2
			1	2	1	3
2	1	2				
3						
	1	1			1	1
1			1	2	1	2
3						
			1	1	1	1
			1	2		
1	1	2				
					1	1
2	1	2				
1	1	2				
			1	2	1	2
			1	2		

					1	2
2	1	1			1	2
1	1	1				
			1	2		
			1	1		
2			1	1		
	1	2	1	2		
1						
3	1	2				
3					1	2
	1	1				
3	1	2			1	1
					1	3
					1	3
3					1	2
			1	1	1	1
			1	4		
4			1	2	1	3
			1	3		
3	1	2	1	2		
5						
3					1	2
3	1	2	1	1	1	3
					1	2
3						
2					1	3
			1	5		
					1	1
			1	2		
					1	1
2			1	2	1	3
	1	2	1	5		
			1	2		
1	1	1	1	2	1	1
3						
3	1	1				
					1	4
	1	1	1	2	1	3

2					1	3
1					1	5
1					1	1
1						
3	1	1				
5						
			1	2		
1						
3	1	2				
3			1	2		
1					1	1
1			1	2	1	5
			1	2		
			1	2		
			1	1	1	3
					1	1
4	1	2			1	2
2			1	2	1	2
			1	1		
					1	1
4						
3	1	1				
4			1	1	1	4
			1	2		
1			1	2		
5						
			1	1		
2			1	2		
			1	2	1	3
3						
					1	3
2						
			1	1		
					1	3
			1	1		
3	1	2			1	1
2	1	1	1	2	1	3
					1	5
			1	2	1	3
					1	2
3	1	1			1	1
1						

			1	2	1	1
			1	3		
					1	3
	1	1				
	1	1				
	1	1			1	2
2			1	5	1	3
4	1	1			1	4
					1	2
2						
1	1	1	1	5	1	3
3	1	1				
3	1	1				
	1	1				
3	1	1			1	3
					1	2
3	1	1	1	1		
3					1	3
			1	2		
			1	2	1	3
	1	1	1	2	1	5
1	1	1				
	1	1			1	1
			1	2		
			1	2		
3			1	1	1	3
3			1	3		
3	1	1				
2						
3	1	1				
5			1	2	1	4
3	1	1	1	1	1	1
3						
			1	5	1	1
			1	1		
	1	1				
	1	1				

3	1	3	1	5	1	3
			1	2		
2						
			1	1		
1						
1	1	1	1	1		
1			1	1		
			1	1		
					1	2
			1	1		
4						
3						
			1	1		
			1	1	1	2
1	1	1				
2	1	2				
5						
					1	3
3	1	2				
4						
			1	1		
3						
5						
					1	1
					1	3
2						
			1	2	1	2
	1	1				
	1	1				
	1	1				
			1	2		
3	1	2	1	3		
			1	1		
			1	1		
3	1	1				
3						

2	1	2				
1			1	3	1	4
			1	1	1	1
1	1	2			1	5
1						
3	1	2				
2	1	1				
2						
3	1	1				
					1	2
4					1	2
1	1	2	1	2		
1						
3						
4	1	1	1	2	1	3
2					1	1
1						
					1	1
1						
					1	2
3						
5	1	1				
3	1	1	1	3		
			1	1		
3	1	3				
3					1	3
1						
2	1	1	1	1	1	3
3	1	1				
2					1	3
3						
	1	1				
	1	1				
2						
			1	1		
5			1	2	1	3
3						
1						
	1	1				
1	1	1	1	2	1	1
			1	1	1	1

1	1	1	1	1		
4	1	3	1	2	1	2
3	1	2				
1			1	3		
1	1	1			1	3
3						
3			1	2		
			1	1	1	1
	1	1				
2			1	2		
1	1	2				
			1	5		
	1	2				
3					1	3
3			1	3		
5						
5						
3	1	1				
3						
					1	1
4						
1						
4						
3						
	1	1	1	1		
			1	1		
3						
5	1	1				
2	1	1				
3					1	3
	1	2				
3	1	1			1	3
			1	1		
			1	2		
4	1	3				
2	1	1			1	4
4						
1	1	1				
2	1	1			1	2

3	1	2				
2	1	1				
4			1	1		
	1	1			1	2
1						
	1	1				
3			1	2		
3			1	5	1	5
5			1	2		
3						
3	1	3			1	2
			1	1		
4						
					1	3
1					1	4
					1	1
			1	4		
3			1	2	1	3
			1	1	1	1
1			1	2	1	1
			1	1		
	1	1				
1						
3	1	3				
2						
3						
	1	1				
					1	2
2						
	1	1				
1	1	2			1	1
			1	2	1	2
					1	1
3	1	2				

	1	1				
5	1	1	1	1	1	3
5						
4						
1	1	1	1	1		
					1	1
1	1	2				
			1	1		
	1	2				
					1	3
1	1	1				
			1	1		
			1	3	1	3
1	1	1	1	2		
					1	1
2			1	1		
1						
1	1	2			1	5
2	1	2				
	1	1				
2			1	2	1	3
	1	1				
1	1	1				
3						
	1	2				
2	1	2	1	2	1	3
1	1	2			1	2
2	1	1	1	2		
			1	3		
2	1	1	1	2		
4			1	1	1	2
					1	3
2					1	1
1					1	2
			1	2	1	2
3	1	2				
3	1	1				

5			1	3	1	2
3	1	2				
2	1	1	1	2	1	4
			1	4		
3	1	2			1	1
					1	3
2	1	1				
2					1	2
3	1	2				
					1	2
			1	2	1	3
3	1	2				
1						
3	1	2				
	1	1				
			1	2		
2			1	2	1	5
3	1	2				
					1	1
			1	2	1	3
			1	3	1	2
	1	1				
	1	1	1	1		
	1	1				
					1	3
					1	1
1					1	1
	1	2				
			1	1		
1					1	2
			1	2	1	3
2	1	2				
3	1	2				
2	1	1				
1			1	2	1	2
			1	3	1	3
4	1	2	1	3	1	3
2	1	2				
					1	1
3	1	1			1	1

2	1	1				
1			1	3	1	4
1	1	1				
3	1	2				
	1	2			1	1
			1	1		
			1	1		
					1	1
			1	2		
4						
	1	1				
4	1	2			1	1
3			1	2		
			1	2	1	2
			1	2		
4	1	2	1	2	1	3
1	1	1				
	1	1			1	3
			1	4		
					1	3
			1	1		
					1	1
			1	2		
			1	4		
4			1	2	1	3
3	1	2	1	1		
1						
					1	3
			1	5		
3						
					1	3
			1	2		
3	1	1	1	2	1	3
2	1	2			1	4
					1	5
			1	2		
3	1	1				
1			1	3	1	3
			1	1		
			1	2	1	3
1					1	1

	1	2				
4						
3	1	2				
			1	2		
2			1	5	1	2
3	1	2				
3			1	3		
3	1	2				
2						
1	1	1				
			1	3		
3						
1						
3			1	2		
			1	1		
2					1	4
	1	2	1	4	1	2
1	1	2				
			1	1		
					1	5
4			1	1		
4			1	3		
3			1	3		
			1	31	1	1
					1	3
4	1	2				
					1	3
1						
	1	2				
4	1	2	1	2	1	3
			1	3		
2	1	3				
4	1	2	1	1		
2	1	1			1	1
3	1	1				
5	1	2			1	3
3						
2					1	3
5						

3	1	2			1	3
1						
3						
4						
					1	1
3						
2	1	1				
	1	3			1	3
2			1	4	1	3
2	1	1	1	2	1	2
5	1	3	1	2	1	3
	1	1				
2	1	2				
			1	2		
			1	2		
			1	1	1	3
3	1	2				
	1	2	1	1		
					1	2
	1	2				
2	1	1				
4	1	2				
5	1	1				
1	1	2			1	1
3					1	3
	1	1				
			1	2	1	5
2	1	1				
			1	2	1	3
2	1	1			1	3
2						
	1	1				
					1	2
			1	2	1	3
			1	1	1	1
					1	1
4						
4	1	2			1	1
			1	2	1	3
3	1	3	1	4	1	3

2	1	1	1	2		
1	1	2				
	1	2				
			1	2	1	3
3						
			1	3		
			1	2		
			1	3	1	1
4					1	1
5						
4						
2					1	1
3					1	3
					1	1
1					1	3
	1	2				
3	1	2				
4	1	1	1	3		
					1	2
1	1	2				
	1	2	1	1		
1						
3			1	2		
4						
2	1	1			1	2
					1	3
			1	2		
			1	2		
1	1	2			1	3
2						
			1	4	1	3
2					1	1
					1	3
			1	2		
3			1	2		
			1	1		
4						
			1	1	1	4
					1	2
3						
2	1	2				
3						
	1	1			1	3

3	1	2	1	2		
1	1	2			1	2
					1	2
	1	1				
					1	1
5	1	2				
			1	2	1	3
			1	5	1	3
2						
	1	1				
2						
3						
5						
1			1	2		
					1	2
					1	3
					1	3
2	1	2				
4			1	2	1	3
4			1	2		
4	1	1			1	3
			1	1		
5	1	2			1	5
2	1	2	1	2	1	5
2						
2					1	4
			1	4		
5			1	3	1	3
3	1	2	1	2	1	1
			1	3		
5			1	2	1	3
4	1	2	1	2		
2	1	3				
1	1	1	1	2	1	3
1	1	1				
3						
3					1	2
1						
			1	2	1	1
					1	3

2	1	2				
2						
2			1	2	1	3
			1	2		
2	1	1				
2	1	1			1	5
2			1	5	1	1
					1	4
			1	1		
2						
	1	3				
1					1	3
			1	3	1	2
2			1	5		
3	1	1				
2			1	2	1	3
					1	3
1						
2	1	3	1	2		
					1	3
	1	1				
3					1	3
			1	2	1	2
					1	3
					1	3
					1	2
			1	1		
2	1	3			1	2
	1	1			1	3
3						
1	1	2				
2			1	2	1	4
2					1	3
	1	2	1	2	1	3
1	1	1			1	1
1	1	2				
2			1	2	1	2
1	1	1				
1	1	2			1	3
					1	1
3						

			1	1		
2	1	1	1	3	1	2
2			1	2	1	3
1	1	2				
					1	4
					1	3
					1	3
			1	1		
3			1	2	1	3
2						
2			1	5		
	1	1	1	2		
1						
1			1	1		
			1	1		
1					1	1
3						
			1	2	1	2
3			1	3		
2						
			1	2	1	3
					1	4
			1	2	1	5
	1	2	1	5		
	1	1	1	2	1	4
1	1	1	1	2		
1			1	2	1	2
			1	2	1	2
2	1	2	1	2	1	3
3	1	3			1	1
	1	1				
2	1	1	1	3		
6	1	1			1	3
4						
			1	2	1	3

2	1	2				
3			1	2	1	3
			1	3	1	3
2						
3					1	1
			1	1		
1					1	3
					1	3
1						
			1	2		
1	1	2			1	3
1						
1			1	1		
					1	3
	1	1				
2						
2	1	2			1	3
3			1	4		
					1	3
1						
2	1	2	1	3		
1			1	2	1	1
					1	5
			1	2		
					1	3
2					1	2
2			1	2	1	2
	1	1				
3	1	1				
					1	3
1			1	2		
3					1	3
			1	5	1	3
5						
3	1	1				
4	1	2			1	2
4						

4			1	2		
			1	2		
1						
5	1	2				
					1	1
5	1	1				
1						
1						
	1	1			1	3
			1	2		
	1	2			1	3
1			1	2	1	3
1					1	4
					1	2
4						
			1	1		
3	1	1			1	3
1						
					1	2
			1	1		
1	1	1				
1			1	2	1	3
5						
	1	1			1	2
5						
1	1	1				
3			1	1		
			1	2	1	3
3						
3						
1	1	1				
3	1	3				
3	1	2				
			1	2		
1						
2						
					1	1

4	1	2				
2	1	4			1	2
2	1	1			1	1
4			1	2	1	3
2	1	2				
1	1	2				
			1	1		
2	1	1				
1	1	2				
1	1	2	1	2	1	3
1						
					1	4
1	1	2				
2	1	1				
3						
3	1	2				
					1	3
					1	1
1						
			1	1		
					1	1
4	1	2			1	3
			1	2		
1					1	1
3					1	2
4						
					1	3
2	1	1				
2	1	1				
					1	3
1	1	1				
2	1	2				
			1	3	1	3
			1	2		
2						
1						
1						
			1	1		

4	1	2			1	2
4						
4	1	1				
1						
2						
	1	1			1	3
			1	1		
					1	3
2			1	3		
	1	2				
	1	1				
1	1	1				
					1	2
			1	1	1	1
			1	1		
5	1	2			1	2
2	1	1	1	4		
			1	4		
			1	1	1	1
3	1	2			1	3
					1	1
1						
1						
2						
					1	3
1	1	2			1	2
					1	1
3					1	2
3	1	1	1	1		
			1	1		
1						
1					1	2

1	1	1	1	5		
1	1	1				
4						
1					1	3
1						
2					1	1
5	1	2	1	2		
1	1	1				
1			1	2		
			1	2	1	3
5	1	1			1	2
			1	1		
					1	1
2	1	1			1	2
			1	1	1	1
			1	1		
2					1	3
3	1	1				
			1	2	1	2
3						
			1	2	1	2
	1	1			1	1
1	1	2				
					1	4
			1	2		
			1	2	1	3
1			1	2	1	1
3			1	2	1	4
1	1	1				
3	1	2	1	1	1	2
					1	3
1					1	1
3						
1			1	2		
			1	1		
2						
			1	2	1	1

	1	1	1	2		
4	1	2				
			1	1	1	1
			1	1	1	2
5	1	2			1	2
					1	1
1						
			1	2		
3			1	2	1	2
			1	1		
1						
2					1	1
3	1	1				
			1	2	1	3
	1	1				
			1	1		
	1	1			1	2
1	1	1			1	1
5	1	2	1	2		
					1	2
	1	2	1	2		
	1	1	1	2	1	3
			1	2	1	2
2	1	2				
					1	3

2	1	1				
3	1	2				
1						
2	1	2				
			1	1	1	1
			1	3		
					1	5
					1	3
3	1	3				
1	1	2			1	1
3						
	1	2			1	2
2	1	1	1	1		
4						
3	1	4	1	2	1	2
	1	1	1	2	1	3

					1	3
3						
2					1	3
3	1	2				
2						
3	1	1				
3						
3	1	1			1	2
4	1	1	1	1		
			1	5	1	3
			1	2		
4						
3			1	1	1	3
3	1	2			1	1
2					1	1
					1	3
1	1	1				
2			1	3		
			1	2	1	1
			1	2	1	3
			1	1	1	1
					1	3
1	1	1				
					1	1
1	1	2	1	3		
1						
1	1	1				
3	1	2				
			1	4	1	2
2			1	2	1	2
5					1	3
					1	3
2	1	1	1	2		
1			1	2	1	3
			1	1		
1	1	1	1	3	1	2

	1	2				
			1	1		
	1	1				
1	1	2				
					1	1
5					1	3
1						
1	1	2				
5			1	2	1	1
			1	2		
			1	2	1	3
					1	2
1					1	3
1						
4	1	1				
1						
4						
					1	1
1			1	2	1	5
2	1	2				
1	1	1				
			1	2		
2						
1					1	1
3	1	1				
					1	3
			1	1		
1	1	3				
			1	2		
			1	2		
2	1	1				
2			1	2		
			1	1		
2			1	2		
			1	2	1	1
			1	2	1	1
2			1	2		
1			1	1	1	3
	1	1				
			1	1		
3	1	1	1	2		
					1	3

	1	1	1	2	1	1
			1	3	1	1
5						
			1	2	1	3
					1	1
4	1	1			1	2
					1	1
			1	2		
2						
			1	2		
	1	1			1	1
2						
			1	2	1	1
2	1	1			1	1
1	1	2				
			1	2		
1			1	2	1	4
1						
			1	2	1	2
1						
3	1	2				
2						
2	1	2	1	4		
4	1	3				
1						
3						
5	1	4				
3			1	4		
2						
2			1	2	1	3
1						

AIS_Abdomen	AIS_Score_Abdomen	AIS_Upper_Extremity	AIS_Score_Upper_Extremity	AIS_Lower_Extermity
		1	1	
		1	1	
				1
		1	1	1
				1
1	2	1	1	1
		1	1	
		1	2	1
		1	1	
		1	1	
		1	2	
		1	2	
				1
				1

		1	1	
		1		
1	1			
				1
1	5	1	2	1
		1	1	1
1	2			
		1	2	1
		1	2	
		1	1	1
				1
		1	1	1
				1
		1	2	
		1	1	
1	1			
				1
		1	2	1
		1	1	1

		1	1	
				1
		1	1	
				1
		1	2	
		1	1	
		1	1	
				1
		1	1	
1	3	1	2	1
				1
				1
1	1	1	1	
		1	2	1
		1	1	1
		1	1	
1	3	1	2	1
				1
		1	1	
		1	1	1
		1	1	
				1
				1

				1
		1	1	1
		1	1	
				1
1	5	1	2	1
				1
				1
1	2	1	2	1
		1	1	1
		1	2	1
		1	2	
1	2	1	2	1
				1
		1	1	
				1
				1
				1
		1	1	1
				1
		1	1	1
		1	2	
		1	2	
		1	1	1
				1
		1	1	
1	1			
		1	2	1
		1	1	1

				1
1	2	1	2	1
		1	2	1
1	1			
1	4			1
		1	1	1
		1	1	1
		1	2	1
1	1			
		1	1	1
				1
				1
1	1	1	1	1
				1
		1	3	1
		1	2	1
				1
		1	1	
				1
				1
		1	1	
				1
		1	2	1
		1	1	1
				1
				1
		1	1	
		1	2	1
		1	1	
				1
		1	2	1

				1
				1
		1	1	1
		1	1	
		1	2	
				1
		1	1	
		1	1	1
				1
		1	2	
		1	2	
		1	2	1
		1	2	
		1	1	1
1	1			
		1	3	1
		1	1	1
				1
				1
1	2	1	1	1
				1
		1	1	1
		1	2	1
		1	2	
		1	2	1
1	5			
		1	1	
		1	2	1
		1	1	
1	1	1	1	
		1	1	1
		1	1	1
		1	1	
1	2			

		1	2	1
1	3			1
1	1			
		1	2	1
		1	1	1
1	2			
1	3	1	1	
		1	2	
1	4			1
		1	1	1
1	2	1	2	1
				1
		1	2	1
				1
		1	1	
1	5			1
		1	2	
		1	2	
		1	2	
		1	2	
				1
1	2	1	2	
				1
		1	2	1
		1	1	1
1	2	1	3	1
1	2			
		1	1	1
1	1	1	1	1
				1

				1
		1	2	1
		1	1	
1	2			1
				1
				1
		1	2	1
				1
				1
		1	2	1
		1	2	
		1	2	
		1	1	
		1	2	
		1	2	
		1	2	
1	2	1	2	
1	2	1	2	
		1	13	1
				1
		1	2	
		1	1	1
				1
		1	1	
		1	1	
		1	1	
				1
				1
		1	1	
				1

1	2	1	2	
		1	1	
				1
				1
				1
				1
		1	1	
1	1			1
		1	1	1
				1
		1	1	
		1	1	1
		1	2	1
		1	2	
		1	1	
				1
				1
		1	1	
		1	1	
		1	2	
				1
1	1			
		1	1	
		1	1	1
				1
				1
		1	1	

		1	2	1
				1
		1	2	1
				1
				1
		1	2	
		1	2	1
		1	2	
1	3	1	1	
1	3			
		1	2	
				1
		1	1	1
		1	2	
		1	2	1
		1	1	1
1	3			
1	1			1
		1	1	1
1	3			1
		1	1	1
		1	2	
		1	2	
		1	2	1
		1	2	1
				1
		1	1	
		1	2	1

		1	1	
		1	2	1
		1	2	1
				1
1	2			
				1
		1	2	1
				1
		1	3	
		1	2	
				1
		1	1	
				1
1	2			1
				1
				1
				1
		1	1	
				1
1	2	1	1	
1	3	1	2	

		1	1	
				1
		1	1	1
				1
		1	2	
1	3	1	2	1
				1
		1	1	
		1	1	
1	2			
1	5			
1	1			
1	1			
				1
		1	1	1
1	1	1	2	
				1
				1
		1	1	1
				1
				1
		1	2	
				1
		1	1	1
				1
1	1			1
1	1			

		1	1	
		1	2	
		1	1	
		1	1	1
		1	1	
				1
				1
		1	1	1
				1
				1
		1	2	
		1	1	
		1	2	1
		1	2	
		1	1	1
		1	1	
1	2	1	2	
		1	1	
				1
		1	1	1
1	2			
		1	1	
				1
		1	2	1

		1	1	
		1	2	
		1	3	1
		1	1	
		1	1	
				1
		1	2	1
		1	1	1
1	2			
		1	1	
		1	3	1
1	2	1	3	1
		1	1	
				1
1	3			1
		1	2	
		1	1	1
		1	1	1
		1	1	1
		1	1	1
1	1			
		1	1	
1	2			1
		1	1	1
1	2	1	1	1
				1
1	4	1	2	1
				1
1	2			1
		1	2	

		1	1	
		1	2	1
		1	1	
				1
		1	1	1
		1	3	1
		1	1	
		1	3	1
		1	1	
1	1	1	3	
				1
		1	2	
1	5			
		1	1	
		1	1	1
		1	2	1
				1
				1
				1
				1
1	3			
		1	1	
		1	1	1
		1	1	1
				1
				1
		1	2	1
		1	2	
		1	1	1
1	4			1
1	3			
		1	1	1
		1	1	1
		1	1	1

				1
		1	2	1
		1	1	1
		1	2	1
		1	3	
		1	1	1
		1	2	1
		1	1	1
1	3	1	2	1
		1	1	
		1	1	
1	2	1	3	1
1	2	1	1	
		1	1	1
		1	2	
1	4	1	2	1
		1	1	
		1	1	1
		1	2	1
		1	1	
				1
		1	1	
		1	2	1
		1	1	1
				1
				1
				1
				1
		1	2	1
1	2	1	2	1
1	4	1	1	1
		1	2	

		1	1	
		1	1	1
				1
		1	2	
				1
1	2			
		1	2	1
				1
1	2	1	2	
1	1	1	1	1
1	3			1
		1	1	1
		1	2	1
		1	1	
		1	2	
				1
1	2			
		1	1	
		1	1	
				1
		1	1	1
		1	4	
1	2			
				1
				1
		1	1	
		1	1	
		1	1	1
		1	1	1
1	4	1	2	
		1	2	
				1
		1	1	1
1	2			
1	2	1	2	
				1

				1
		1	1	1
1	3			1
		1	2	
1	2			
1	2			
		1	1	1
1	2	1	1	1
		1	2	1
		1	1	
		1	2	1
1	4			
		1	1	1
		1	1	
		1	1	1
				1
				1
				1
				1
		1	2	1
		1	2	1
				1
		1	1	1
				1
				1
		1	2	
1	2			1
1	2	1	2	1
1	2	1	1	

		1		1
		1	1	
		1	1	1
1	1			
1	1			
1	2			
		1	2	1
		1	1	1
				1
1	1	1	2	1
		1	1	1
		1	1	
		1	1	1
				1
1	5	1	2	
				1
		1	2	
		1	3	1
		1	2	
		1	2	1
		1	2	1
		1	2	
1	4	1	2	1
		1	2	
1	3			1
1	4	1	2	1
1	3	1	2	1
1	2			
				1
		1	1	
		1	1	
1	1	1	1	1
				1
				1
1	1			
		1	1	

		1	2	
1	3	1	2	
				1
				1
		1	2	
		1	1	1
1	2	1	1	1
		1	1	1
		1	1	
				1
		1	2	1
		1	1	1
1	1	1	1	
1	1			
1	2	1	2	
1	2			
		1	1	
1	4			1
1	1			
		1	2	
				1
		1	2	1
		1	2	
		1	2	
				1
		1	1	1
1	2	1	2	1
1	4			1
		1	2	
				1
		1	2	1
				1
		1	2	
		1	1	
		1	2	

		1	3	1
1	2			1
		1	2	
1	3	1	1	
				1
		1	2	1
1	1	1	2	1
		1	1	
1	2			
				1
		1	1	
				1
		1	2	1
		1	2	
		1	2	
		1	1	
				1
				1
		1	3	
1	4			
		1	2	1
		1	2	1
1	2			1
		1	1	1
		1	1	1
		1	2	
				1
		1	1	
				1
		1	2	
		1	2	
		1	1	1
1	2			1
		1	2	

		1	1	
1	3	1	2	1
				1
		1	2	1
		1	1	
1	2			
		1	2	
		1	1	
1	1	1	2	1
		1	2	1
1	2	1	2	1
				1
		1	2	
1	1			
		1	1	1
		1	2	1
		1	1	
1	4			
				1
		1	2	
1	1			1
				1
		1	1	1
		1	2	
				1
				1
		1	2	
				1
				1
		1	1	1

		1	1	
				1
		1	2	
		1	2	
		1	2	
1	2			1
		1	2	1
1	3			1
				1
		1	2	1
		1	1	
1	2			
		1	1	1
				1
		1	2	
		1	1	
1	2	1	1	1
		1	1	
		1	1	
		1	1	1
		1	2	
		1	1	1
		1	1	1
				1
1	1			
		1	1	1

				1
				1
		1	2	
				1
1	2			1
				1
				1
		1	2	1
1	4			1
		1	2	
		1	1	
1	2			1
				1
1	1			1
1	1	1	1	1
				1
1	2	1	2	1
		1	1	
		1	2	
		1	2	1
1	4			1

				1
1	2			
		1	2	
		1	1	
1	2			
				1
				1
		1	1	1
		1	2	
		1	1	1
1	3	1	1	1
				1
		1	1	
				1
				1
				1
		1	1	
				1
1	1	1	2	
1	2			
		1	1	1
		1	3	1
		1	1	1
				1
				1
		1	1	
				1
				1
				1
				1

		1	2	
				1
		1	1	1
		1	3	1
				1
		1	2	
		1	2	
		1	1	1
1	2	1	1	1
		1	1	
				1
		1	2	
		1	2	
				1
		1	1	
		1	2	1
		1	3	1
				1
1	3	1	2	1
1	1	1	2	1
		1	2	
		1	2	
				1
		1	1	1
				1
		1	2	
		1	2	1
		1	1	1
				1
1	1	1	1	1
		1	3	

				1
		1	1	1
				1
		1	2	1
		1	2	1
				1
				1
		1	2	
		1	1	1
		1	1	
1	2	1	3	1
				1
1	4	1	2	
				1
		1	2	1
				1
		1	2	
		1	1	
				1
		1	1	1
1	4	1	2	
				1
		1	1	1
		1	1	
1	2	1		1
				1
1	1			

		1	2	
1	1	1	2	1
1	2			
				1
		1	2	
		1	1	
1	2			1
		1	2	
		1	2	
				1
				1
		1	1	1
				1
				1
1	1			
1	1			
		1	2	1
		1	2	
		1	1	1
1	2	1	2	1
				1
		1	2	
				1
		1	1	1
1	1			1
		1	1	
				1
		1	2	
		1	2	
		1	2	
		1	1	1
		1	2	1
		1	2	1

		1	1	1
		1	1	1
		1	2	
				1
		1	1	1
		1	2	1
		1	1	1
		1	2	1
				1
				1
1	1	1	1	1
		1	2	1
		1	2	1
		1	1	
				1
		1	1	1
1	4	1		1
		1	1	1
				1
1	3	1	1	
				1
		1	1	
		1	2	1
		1	1	1
		1	3	1
		1	1	
		1	3	1
		1	1	1
1	4	1	1	1
				1
		1	2	1
1	1	1	1	1
				1
1	4			1

1	1	1	1	1
		1	2	1
1	1			
				1
				1
		1	2	
		1	2	1
		1	1	1
1	1			
		1	2	1
1	2			1
1	1			
		1	1	
		1	2	1
		1	2	1
		1	2	
		1	2	1
1	4	1	1	
		1	2	
1	3			1

AIS_Score_Lower_Extremity	AIS_External	AIS_Score_External	ISS_Score	Anzahl_Diagnosen
				5
				3
				4
				1
2				2
				7
				4
				1
				4
1				5
1				6
3				5
				3
	1	1		2
				3
2				4
				6
				2
				5
				2
				2
				4
				1
				3
				3
				6
				2
				1
				4
				2
				3
				1
				1
				1
				1
				1
				10
				2
1	1	1		4
				7
				2
				2
				1
3				1

			2
			3
			13
			2
			3
			2
			4
	1	1	5
1			4
	1	1	7
			5
3			13
			4
			1
			3
			2
2			6
	1	1	3
			2
	1	1	4
			2
			3
			5
			9
			2
			1
1			6
			2
			8
1			4
1			1
			1
			4
1			10
2			7
			7
			7
			2
			4
			1
			2
			2
3			8
3			13
1	1	1	5

	4
3	2
	3
1	8
	2
	2
	3
	5
	2
	2
	3
	1
	2
	4
3	5
	2
	2
	4
2	13
	2
	2
	2
1	3
	1
2	4
	4
2	6
	2
1	7
	4
	1
	1
3	9
2	4
	2
	7
	6
	4
1	5
	2
1	3
1	7
	2
	2

1			2
3			14
			2
			1
			5
			1
3			7
4			15
1			4
3			5
			1
			2
			1
1			12
			2
1			5
			2
			3
			1
			4
1			3
			7
			10
3			8
2			10
			6
			6
2			5
			3
3	1	2	3
2			5
2			8
1			12
1			4
			2
			5
			1
			2
			3
3			5
2			3
			4
			4
			3
2			10
1			7

3	2
	4
3	13
	2
1	6
	1
	10
1	4
1	14
1	7
	2
	1
1	3
	3
	2
	2
1	4
2	9
1	7
	1
	2
1	5
	2
2	6
	3
1	3
1	3
2	5
	3
	4
1	6
4	8
	5
3	4
2	5
1	13
1	5
	2
2	6
	6
3	3
	3
	6
2	5
2	7

3	5
1	5
1	6
	4
	2
2	5
	6
1	14
	2
	7
	3
1	3
	7
	3
2	3
	7
1	6
	3
	2
	9
	1
	13
2	7
1	3
2	3
	6
1	13
1	5
1	2
1	5
3	2
	4
	1
1	4
	1
	4
	3
1	10
	4
	4
3	10
	6
1	6
	4
	12

3	13
3	17
	4
	2
	6
	5
	4
1	4
1	9
	6
	7
	1
1	7
	2
2	4
	6
	1
1	8
2	7
3	7
1	2
	7
	6
	8
3	21
	1
	3
	5
	5
	5
	5
	6
	2
	4
	4
1	3
	7
1	4
3	12
2	9
3	8
	5
1	5
1	10
	2
3	3

1			4
1			4
			2
			1
			1
3			6
			2
3			6
4			11
2	1	1	6
1			1
2			2
2			20
			4
			2
			1
			3
			12
			7
			16
			2
			9
			2
			13
			4
			3
			1
			9
			6
1			4
1			4
			6
2			4
1			3
			20
			4
			8
			2
1			4
2			12
			9
			6
			3
			1
3			4
			2

			11
			2
			5
			3
1			2
1			2
1			1
3			4
			4
			4
			1
			4
1			6
3			2
			4
			4
			2
1			5
			8
			5
			4
			3
3			2
1			8
			7
			2
			2
			2
			4
5			1
1			4
			4
			3
			4
1			3
			1
			5
			3
1			4
3			4
	1	1	11
			2
2			3
			3
			5
			2

			6
2			20
1			4
3			12
1			3
1			7
			5
			4
1			7
			2
			3
			3
			6
1			3
1			5
			9
1			3
1			7
			1
			8
1			5
			2
1			4
			2
3			8
			8
2			3
			6
			1
			6
			8
			2
			11
			8
			4
			5
1			5
			5
1			5
			3
2			11
			7
			4
			4
			5
3	1	1	7

			6
2			13
2			12
3			8
			7
			3
			3
1			4
1			7
	1	1	4
			6
			3
1			4
			2
			2
			4
			5
			6
			5
			6
			2
1			7
			2
			8
			2
1			4
			2
			9
			5
3			3
			5
1			2
4	1	1	3
			4
1			5
			2
			3
	1	1	2
			13
			2
3			9
			4
			6
			6

			15
			1
1			5
	1	1	9
1			7
2			4
			1
			5
			5
2			13
2			9
			7
			2
			9
			4
			5
			2
			7
			1
			2
			2
4			2
2			2
	1	2	1
2			5
			6
			1
2			2
1			5
			2
			8
5			2
			1
			4
3			2
			5
2			1
1			5
2			3
			2
			2
1			7
			4
			3
			6

			3
			17
			3
			1
			2
			4
			2
			1
			3
			2
			1
			3
1			6
			1
2			3
			2
2			2
			2
1			7
1			4
			1
			2
1			3
			3
			2
			2
4			5
			10
1			6
			3
			3
			6
			10
	1	2	8
			6
			1
			4
4			1
1			11
			4
			3
			3
1			5
3			8
			6

	9
	7
3	9
	1
	10
	3
1	4
1	10
1	11
	4
	8
3	3
	5
	2
3	5
	5
3	1
	2
	1
3	11
	6
	3
	4
	10
	2
1	5
1	4
	5
1	5
3	2
	3
	2
	2
	5
	6
	3
	7
4	5
1	7
1	8
2	9
3	16
2	5
1	4
	9
	3

			3
1			11
			4
1			8
1			6
3			5
			2
			2
			1
3			8
	1	2	7
			3
			7
1			4
			3
			2
			11
			6
2			6
			1
3			8
3			1
3			1
1			3
1			3
			3
			1
			7
1			11
			2
1			4
			3
1			4
3			3
1			8
			10
			10
2			7
			1
4			6
			6
3			3
1			3
			2
			6
1			6

1			3
			4
			9
			2
			6
3			10
			3
			4
			4
1			6
3			9
			2
			3
1			4
2			3
			2
1			4
3	1	3	17
			11
			5
			4
4			9
			2
			2
			5
			4
1			6
			5
3			9
			4
1			5
2			6
			12
1			2
			2
1			12
1			8
1			4
1			3
1			6
			8
3			2
1			3
1			9
1			9
			6

	10
1	6
3	4
	6
	2
1	6
	3
	4
	9
1	7
2	2
	12
2	8
	6
	1
	2
2	5
1	5
1	3
	7
	4
	2
2	4
	11
	10
	8
	7
3	4
1	3
	3
	6
	3
2	16
3	1
	3
	3
2	6
2	3
	10
	2
	3
2	3
1	3
	12
	17
2	13

2	8
1	9
	2
3	18
	4
	2
	2
	6
	7
	5
	4
1	4
3	7
2	11
	3
3	8
	3
	4
	12
1	4
	3
	6
1	4
1	7
	7
1	3
1	7
2	3
	3
2	15
3	6
	3
4	6
2	8
3	3
	2
	8
	2
1	7
	5
3	13
3	12
	5
	9
	4
	8

1			22
			9
1			4
			3
			3
			8
			4
2			4
1	1	3	8
1	1	2	3
3			6
1			4
			10
1			8
1			3
			4
3			2
			1
			2
3			6
			4
			10
1			5
			10
1			13
			4
3			14
	1	2	19
2			5
			7
			1
4			20
3	1	1	11
			7
1			9
			9
			4
			10
			5
			2
1			9
4			2
2			4
			5
			7

	4
	7
	13
2	3
1	4
	8
1	4
1	13
1	5
	2
	5
3	1
	1
2	9
	8
	4
1	15
	9
	4
	2
	13
	3
	5
1	4
	7
	8
	3
5	6
2	6
	2
	10
	5
	4
1	9
3	2
1	8
1	7
	8
	3
3	8
1	12
1	4
	6
	3
	2
	5

			2
2			12
2			11
			5
			4
			4
3			1
3			7
	1	1	3
1			11
			2
			5
			6
2			5
			3
1			3
			2
			2
3			3
			3
			10
			5
1			4
3			9
			1
			2
			3
3			2
			3
			7
2			5
3			13
1			7
			6
			6
1			4
			15
1			12
			3
			4
1			2
			1
			2
1			6
2			8
			7

			6
3			12
			8
1			3
			5
			6
			3
			4
2			10
			2
			2
			2
			4
			2
			4
2			8
			2
3			3
			5
			6
			6
1			6
1			3
			9
	1	1	5
			4
2			3
			3
1			5
			2
			1
2			2
			5
2			5
3			5
1			5
			4
2			3
2			3
			10
			7
1			3
1			4
			9
2			4
			4

			6
			2
1			2
			11
			1
			6
			2
			2
			1
			4
			4
			5
1			5
	1	1	12
3			11
			1
2			3
2			1
			1
1			7
			2
			8
			2
1			6
1			3
			3
			11
	1	1	3
			3
3			7
			5
			6
			1
			2
1			3
			13
			2
			4
1			4
			9
1			8
1			4
			2
			2
			2
1			4

1			8
1			1
			15
1			6
3			11
			5
			7
1			2
			4
1			9
			9
1			5
			1
1			6
			2
			4
			5
			1
			1
	1	5	1
	1	5	2
			3
3			5
1			2
1			3
			2
1			5
			1
			8
			1
1			2
			2
			6
			4
			2
			4
			2
2			8
			4
			4
			10
			1
			2
2			9
3			7
			2

			10
	1	3	1
2	1	5	2
			4
			6
			3
			6
			5
1			1
			2
2			6
2			6
			2
			3
1			6
	1	1	4
1			5
3			2
			3
1			3
1			13
			5
			4
			3
1			2
			3
			10
	1	3	2
			1
			1
			2
			2
3			5
			5
			7
2			4
3			8
1			7
1			4
2			3
			3
5	1	2	2
1			4
3			3
			3
3			2

			7
			3
2			3
1			6
			2
2	1	3	7
2			16
			6
			2
			7
			6
			2
1			7
1			10
			5
1			2
			1
			9
			9
			17
			4
			3
1			6
			1
			4
3			9
3			3
3			3
			1
3			14
1			9
			2
			11
3			1
1			5
3			7
			12
3			5
	1	1	4
1			3
2	1	1	13
1			10
			2
			7
			2

1			6
2			12
1			3
1			7
3			7
			1
			2
			2
			11
2			2
			2
1			7
			8
2			12
			2
			3
3			7
			8
			10
2			3
			2
	1	1	29
			4
			10
			1
3			3
3			3
2			3
			6
			11
1			3
			4
1			6
			2
			8
			2
			7
3			2
1			9
			4
			4
			5
			3
3			13
3			2
			9

			3
1			7
			8
			3
2			8
			8
			8
			7
			9
			2
3			3
			1
			1
			6
			11
			8
1			3
1			3
			3
1			3
1			6
1			4
			4
			2
1			8
			5
1			4
			3
2			7
	1	1	6
3			1
			1
1			2
1			4
3			4
			4
4			4
			4
			11
			8
			8
1			6
3			7
3			4
			7

			1
1			4
1			4
			6
3	1	1	6
1	1	1	4
1			8
			2
1	1	1	7
			9
2			6
2			2
2			8
1			8
1			6
2			5
			9
1			3
			3
1			8
3			13
3			15
			4
			2
1			4
			5
			6
			3
3			2
			3
1			8
2			4
3			7
			5
			6
			2
3			4
			7
4	1	1	9
2			9
3			10
			5
2			6
1			6
1			11
2			6

1			14
			4
			7
2			8
			2
3			4
3			4
			1
			4
1			5
2			9
			5
			7
			4
3			7
			1
3			3
			6
			3
			4
3	1	1	4
			1
			7
3			4
			3
			7
			5
1			6
			9
			6
			7
			3
3	1	4	12
			2

ISS_ScoreISS_Score	Polytrauma	VerstorbenaufNF	VerstorbenimVerlauf	IBE	Verlauf_IBE
5	2	2	2	2	1
2	2	2	2	2	1
11	2	2	2	2	1
1	2	2	2	2	1
8	2	2	2	2	1
17	1	2	2	2	1
14	2	2	2	1	1
1	2	2	2	2	1
6	2	2	2	2	1
3	2	2	2	2	1
3	2	2	2	2	1
13	2	2	2	2	1
3	2	2	2	2	1
2	2	2	2	2	1
16	1	2	2	1	1
9	2	2	2	2	1
6	2	2	2	1	1
2	2	2	2	2	1
13	2	2	2	2	1
5	2	2	2	2	1
5	2	2	2	2	1
18	1	2	2	2	1
1	2	2	2	2	1
2	2	2	2	2	1
25	1	2	1	2	1
	2	2	2	2	1
11	2	2	2	1	2
5	2	2	2	2	1
1	2	2	2	2	1
13	2	2	2	2	1
9	2	2	2	2	1
18	1	2	2	2	1
4	2	2	2	2	1
1	2	2	2	2	1
4	2	2	2	2	1
1	2	2	2	2	1
1	2	2	2	2	1
24	1	2	2	1	1
9	2	2	2	2	1
3	2	2	2	2	1
5	2	2	2	2	1
4	2	2	2	2	1
2	2	2	2	2	1
25	1	2	2	1	1
9	2	2	2	2	1

2	2 2	2	2 1
9	2 2	2	1 1
14	2 2	2	1 1
2	2 2	2	2 1
29	1 2	2	2 1
2	2 2	2	2 1
2	2 2	2	2 1
10	2 2	2	1 1
3	2 2	2	2 1
14	2 2	2	2 1
2	2 2	2	2 1
43	1 2	2	1 1
4	2 2	2	2 1
4	2 2	2	2 1
10	2 2	2	2 1
2	2 2	2	2 1
12	2 2	2	1 1
2	2 2	2	2 1
13	2 2	2	2 1
9	2 2	2	1 1
4	2 2	2	2 1
	2 2	2	2 1
10	2 2	2	2 1
13	2 2	2	2 1
22	1 2	1	2 1
2	2 2	2	2 1
1	2 2	2	2 1
6	2 2	2	2 1
1	2 2	2	2 1
22	1 2	2	1 1
2	2 2	2	2 1
1	2 2	2	2 1
4	2 2	2	2 1
11	2 2	2	2 1
11	2 2	2	2 1
13	2 2	2	2 1
11	2 2	2	2 1
14	2 2	2	2 1
2	2 2	2	2 1
3	2 2	2	2 1
1	2 2	2	2 1
1	2 2	2	2 1
2	2 2	2	2 1
22	1 2	2	2 1
43	1 2	1	1 1
11	2 2	2	2 1

10	2 2	2	2 1
10	2 2	2	2 1
6	2 2	2	2 1
14	2 2	2	1 1
2	2 2	2	2 1
1	2 2	2	2 1
6	2 2	2	2 1
26	1 2	1	2 1
2	2 2	2	2 1
2	2 2	2	2 1
6	2 2	2	2 1
1	2 2	2	2 1
2	2 2	2	1 1
3	2 2	2	2 1
35	1 2	2	1 1
2	2 2	2	2 1
25	1 2	1	1 1
17	1 2	2	2 1
22	1 2	2	1 1
26	1 2	2	1 1
9	2 2	2	2 1
2	2 2	2	2 1
3	2 2	2	2 1
1	2 2	2	2 1
4	2 2	2	2 1
2	2 2	2	2 1
14	2 2	2	2 1
9	2 2	2	2 1
3	2 2	2	2 1
3	2 2	2	2 1
4	2 2	2	2 1
1	2 2	2	2 1
27	1 2	2	2 1
6	2 2	2	2 1
2	2 2	2	2 1
12	2 2	2	2 1
19	1 2	2	1 1
6	2 2	2	2 1
10	2 2	2	2 1
2	2 2	2	2 1
	2 2	2	2 1
3	2 2	2	2 1
6	2 2	2	2 1
5	2 2	2	2 1
	2 2	2	2 1
2	2 2	1	1 1

2	2 2	2	2 1
27	1 2	2	1 1
2	2 2	2	2 1
1	2 2	2	2 1
10	2 2	2	2 1
1	2 2	2	2 1
23	1 2	2	1 1
57	1 2	2	1 1
2	2 2	2	2 1
2	2 2	2	2 1
25	1 2	2	1 1
2	2 2	2	2 1
25	1 2	1	1 1
12	2 2	2	1 1
25	1 2	2	1 1
3	2 2	2	2 1
2	2 2	2	2 1
9	2 2	2	1 1
9	2 2	2	2 1
26	1 2	2	1 1
5	2 2	2	2 1
12	2 2	2	2 1
13	2 2	2	2 1
17	1 2	2	1 1
36	1 2	2	1 1
3	2 2	2	2 1
20	1 2	2	1 1
6	2 2	2	2 1
10	2 2	2	1 1
13	2 2	2	1 1
17	1 2	2	2 1
9	2 2	2	2 1
14	2 2	2	1 1
2	2 2	2	2 1
2	2 2	2	2 1
14	2 2	2	2 1
1	2 2	2	2 1
13	2 2	2	2 1
5	2 2	2	2 1
14	2 2	2	2 1
5	2 2	2	2 1
3	2 2	2	2 1
6	2 2	2	2 1
13	2 2	2	2 1
17	1 2	2	2 1
17	1 2	2	2 1

9	2 2	2	2 1
5	2 2	2	2 1
27	1 2	2	1 1
2	2 2	2	2 1
13	2 2	2	2 1
1	2 2	2	2 1
14	2 2	2	2 1
18	1 2	2	1 1
12	2 2	2	2 1
11	2 2	2	2 1
1	2 2	2	2 1
1	2 2	2	2 1
6	2 2	2	2 1
3	2 2	2	2 1
2	2 2	2	2 1
2	2 2	2	2 1
2	2 2	2	2 1
17	1 2	2	1 1
8	2 2	2	1 1
1	2 2	2	2 1
1	2 2	2	2 1
3	2 2	2	2 1
2	2 2	2	2 1
8	2 2	2	2 1
5	2 2	2	2 1
10	2 2	2	2 1
4	2 2	2	2 1
6	2 2	2	2 1
3	2 2	2	2 1
3	2 2	2	2 1
6	2 2	2	1 1
29	1 2	2	1 1
11	2 2	2	2 1
	2 2	2	2 1
11	2 2	2	2 1
5	2 2	2	2 1
11	2 2	2	2 1
11	2 2	2	2 1
4	2 2	2	2 1
12	2 2	2	2 1
6	2 2	2	2 1
10	2 2	2	2 1
8	2 2	2	2 1
6	2 2	2	2 1
12	2 2	2	2 1
12	2 2	2	2 1

14	2	2	2	2	1
12	2	2	2	1	1
2	2	2	2	2	1
3	2	2	2	2	1
5	2	2	2	2	1
9	2	2	2	2	1
6	2	2	2	2	1
9	2	2	2	2	1
2	2	2	2	1	1
17	1	2	2	2	1
6	2	2	2	2	1
3	2	2	2	2	1
17	1	2	2	2	1
13	2	2	2	2	1
13	2	2	2	2	1
17	1	2	1	1	1
3	2	2	2	2	1
16	2	2	2	2	1
2	2	2	2	2	1
29	1	2	1	1	1
9	2	2	2	2	1
12	2	2	2	1	1
14	2	2	2	2	1
1	2	2	2	2	1
4	2	2	2	2	1
14	2	2	2	1	1
22	1	2	2	1	1
9	2	2	1	2	1
2	2	2	2	2	1
3	2	2	2	2	1
9	2	2	2	2	1
17	1	2	2	1	1
25	1	2	2	2	1
6	2	2	2	2	1
25	1	2	2	2	1
9	2	2	2	2	1
3	2	2	2	2	1
17	1	2	2	2	1
29	1	2	2	2	1
6	2	2	2	2	1
14	2	2	2	2	1
9	2	2	1	1	1
3	2	2	2	2	1
18	1	2	2	1	1
	2	2	2	2	1
17	1	2	2	1	1

22	1 2	2	1 1
43	1 2	1	1 1
2	2 2	2	2 1
2	2 2	2	2 1
10	2 2	2	1 1
26	1 2	2	1 1
12	2 2	2	2 1
6	2 2	2	2 1
17	1 2	2	1 1
17	1 2	2	2 1
11	2 2	2	2 1
4	2 2	2	2 1
42	1 2	2	1 1
8	2 2	2	2 1
9	2 2	2	2 1
14	2 2	2	1 1
1	2 2	2	2 1
24	1 2	2	1 1
17	1 2	2	1 1
10	2 2	2	2 1
2	2 2	2	2 1
1	2 2	2	2 1
16	1 2	2	1 1
11	2 2	2	2 1
57	1 2	2	1 1
4	2 2	2	2 1
5	2 2	2	2 1
9	2 2	2	1 1
6	2 2	2	1 1
5	2 2	2	2 1
10	2 2	2	1 1
17	1 2	2	2 1
9	2 2	2	1 1
14	2 2	2	2 1
13	2 2	2	1 1
2	2 2	2	2 1
17	1 2	2	1 1
3	2 2	2	2 1
22	1 2	2	1 1
17	1 2	2	1 1
38	1 2	2	1 1
17	1 2	2	1 1
6	2 2	2	2 1
11	2 2	2	2 1
2	2 2	2	2 1
9	2 2	2	2 1

9	2 2	2	1 1
6	2 2	2	2 1
10	2 2	2	2 1
4	2 2	2	1 1
9	2 2	2	2 1
14	2 2	2	2 1
2	2 2	2	2 1
14	2 2	2	1 1
45	1 2	2	1 1
8	2 2	2	2 1
1	2 2	2	2 1
4	2 2	2	2 1
36	1 2	2	1 1
8	2 2	2	2 1
5	2 2	2	2 1
4	2 2	2	2 1
8	2 2	2	1 1
35	1 2	2	2 1
11	2 2	2	2 1
17	1 2	2	1 1
2	2 2	2	2 1
22	1 2	2	1 1
5	2 2	2	2 1
17	1 2	2	1 1
17	1 2	2	2 1
5	2 2	2	2 1
4	2 2	2	2 1
17	1 2	2	2 1
33	1 2	2	1 1
2	2 2	2	2 1
3	2 2	2	2 1
11	2 2	2	2 1
9	2 2	2	2 1
5	2 2	2	2 1
22	1 2	2	1 1
10	2 2	2	1 1
11	2 2	2	2 1
2	2 2	2	2 1
3	2 2	2	2 1
45	1 2	1	1 1
14	2 2	2	2 1
11	2 2	2	1 1
29	1 2	2	1 1
1	2 2	2	2 1
11	2 2	2	2 1
1	2 2	2	2 1

43	1 2	2	1 1
13	2 2	2	2 1
5	2 2	2	1 1
3	2 2	2	2 1
2	2 2	2	2 1
2	2 2	2	2 1
1	2 2	2	2 1
11	2 2	2	1 1
3	2 2	2	2 1
3	2 2	2	2 1
1	2 2	2	2 1
6	2 2	2	2 1
11	2 2	2	2 1
10	2 2	2	2 1
16	1 2	2	1 1
16	1 2	2	1 1
1	2 2	2	2 1
6	2 2	2	2 1
3	2 2	2	2 1
6	2 2	2	2 1
25	1 2	1	2 1
10	2 2	2	2 1
9	2 2	2	2 1
17	1 2	2	1 1
14	2 2	2	1 1
2	2 2	2	2 1
1	2 2	2	2 1
2	2 2	2	2 1
26	1 2	1	1 1
25	1 2	2	2 1
3	2 2	2	2 1
11	2 2	2	2 1
3	2 2	2	2 1
9	2 2	2	2 1
3	2 2	2	2 1
9	2 2	2	1 1
11	2 2	2	2 1
3	2 2	2	2 1
3	2 2	2	2 1
13	2 2	2	1 1
14	2 2	2	2 1
1	2 2	2	2 1
8	2 2	2	2 1
2	2 2	2	2 1
11	2 2	2	2 1
2	2 2	2	2 1

9	2 2	2	2 1
24	1 2	2	1 1
3	2 2	2	2 1
38	1 2	2	1 1
3	2 2	2	2 1
14	2 2	2	2 1
5	2 2	2	2 1
14	2 2	2	2 1
6	2 2	2	2 1
5	2 2	2	2 1
11	2 2	2	2 1
13	2 2	2	2 1
24	1 2	2	1 1
2	2 2	2	2 1
2	2 2	2	2 1
12	2 2	2	2 1
6	2 2	2	2 1
10	2 2	2	2 1
1	2 2	2	2 1
22	1 2	2	2 1
9	2 2	2	1 1
2	2 2	2	2 1
2	2 2	2	2 1
2	2 2	2	2 1
22	1 2	2	2 1
13	2 2	2	1 1
5	2 2	2	2 1
11	2 2	2	2 1
1	2 2	2	2 1
18	1 2	2	1 1
22	1 2	2	2 1
2	2 2	2	2 1
17	1 2	2	1 1
11	2 2	2	2 1
13	2 2	2	2 1
10	2 2	2	1 1
6	2 2	2	2 1
6	2 2	2	2 1
9	2 2	2	2 1
2	2 2	2	2 1
38	1 2	1	1 1
10	2 2	2	1 1
3	2 2	2	2 1
2	2 2	2	2 1
5	2 2	2	2 1
11	2 2	2	2 1

6	2 2	2	2 1
29	1 2	1	1 1
17	1 2	2	1 1
14	2 2	2	2 1
14	2 2	2	1 1
9	2 2	2	1 1
13	2 2	2	2 1
3	2 2	2	2 1
6	2 2	2	2 1
5	2 2	2	1 1
6	2 2	2	2 1
29	1 2	2	2 1
6	2 2	2	2 1
9	2 2	2	2 1
18	1 2	2	1 1
10	2 2	2	2 1
13	2 2	2	1 1
25	1 2	1	1 1
10	2 2	2	2 1
10	2 2	1	2 1
5	2 2	2	2 1
18	1 2	2	2 1
2	2 2	2	2 1
	2 2	2	1 1
17	1 2	2	1 1
9	2 2	2	1 1
3	2 2	2	2 1
2	2 2	2	2 1
10	2 2	2	1 1
26	1 2	1	1 1
14	2 2	2	1 1
34	1 2	2	1 1
2	2 2	2	2 1
17	1 2	2	2 1
14	2 2	2	2 1
19	1 2	2	2 1
9	2 2	2	2 1
5	2 2	2	1 1
2	2 2	2	2 1
	2 1	1	1 1
19	1 2	2	2 1
2	2 2	2	2 1
34	1 2	2	2 1
17	1 2	2	2 1
6	2 2	2	2 1
22	1 2	2	1 1

14	2 2	2	2 1
1	2 2	2	2 1
11	2 2	2	1 1
17	1 2	2	1 1
6	2 2	2	2 1
6	2 2	2	2 1
75	1 2	1	1 1
6	2 2	2	2 1
14	2 2	2	1 1
43	1 2	1	1 1
33	1 2	1	1 1
10	2 2	2	1 1
2	2 2	2	2 1
22	1 2	2	1 1
3	2 2	2	2 1
20	1 2	1	1 1
34	1 2	2	1 1
18	1 2	2	2 1
4	2 2	2	1 1
2	2 2	2	2 1
2	2 2	2	1 1
20	1 2	2	2 1
5	2 2	2	2 1
	2 2	2	2 1
4	2 2	2	1 1
9	2 2	2	1 1
9	2 2	2	2 1
1	2 2	2	2 1
5	2 2	2	2 1
3	2 2	2	2 1
2	2 2	2	1 1
14	2 2	2	1 1
26	1 2	2	2 1
25	1 2	2	1 1
9	2 2	2	1 1
10	2 2	2	2 1
6	2 2	2	2 1
4	2 2	2	2 1
9	2 2	2	2 1
5	2 2	2	2 1
4	2 2	2	2 1
2	2 2	2	2 1
6	2 2	2	2 1
9	2 2	2	2 1
3	2 2	2	1 1
13	2 2	2	1 1

2	2 2	2	2 1
29	1 2	2	1 1
26	1 2	1	1 1
1	2 2	2	2 1
10	2 2	2	2 1
3	2 2	2	2 1
2	2 2	2	2 1
4	2 2	2	2 1
4	2 2	2	1 1
10	2 2	2	2 1
4	2 2	2	1 1
9	2 2	2	2 1
2	2 2	2	2 1
1	2 2	2	2 1
5	2 2	2	2 1
2	2 2	2	2 1
4	2 2	2	2 1
34	1 2	2	2 1
6	2 2	2	2 1
3	2 2	2	1 1
1	2 2	2	2 1
1	2 2	2	2 1
3	2 2	2	2 1
	2 2	2	1 1
5	2 2	2	2 1
5	2 2	2	2 1
2	2 2	2	2 1
17	1 2	2	2 1
17	1 2	2	1 1
3	2 2	2	2 1
10	2 2	2	2 1
10	2 2	2	2 1
6	2 2	2	2 1
22	1 2	2	2 1
12	2 2	2	1 1
8	2 2	2	1 1
9	2 2	2	2 1
30	1 2	2	1 1
16	2 2	2	1 1
24	1 2	2	1 1
13	2 2	2	2 1
5	2 2	2	2 1
5	2 2	2	2 1
6	2 2	2	2 1
22	1 2	2	1 1
10	2 2	2	2 1

33	1 2	2	1 1
17	1 2	2	1 1
29	1 2	1	2 1
16	2 2	2	1 1
14	2 2	2	2 1
10	2 2	2	2 1
3	2 2	2	2 1
9	2 2	2	2 1
33	1 2	2	2 1
9	2 2	2	1 1
11	2 2	2	2 1
10	2 2	2	2 1
17	1 2	2	2 1
2	2 2	2	2 1
13	2 2	2	2 1
14	2 2	2	1 1
9	2 2	2	2 1
2	2 2	2	2 1
4	2 2	2	2 1
43	1 2	2	1 1
14	2 2	2	2 1
6	2 2	2	2 1
14	2 2	2	2 1
22	1 2	2	1 1
2	2 2	2	2 1
2	2 2	2	2 1
2	2 2	2	2 1
10	2 2	2	1 1
3	2 2	2	2 1
9	2 2	2	2 1
1	2 2	2	2 1
5	2 2	2	2 1
17	1 2	2	2 1
6	2 2	2	2 1
13	2 2	2	1 1
6	2 2	2	2 1
14	2 2	2	2 1
21	1 2	2	1 1
3	2 2	2	2 1
12	2 2	2	2 1
22	1 2	2	1 1
41	1 2	2	1 1
8	2 2	2	1 1
3	2 2	2	2 1
11	2 2	2	1 1
5	2 2	2	2 1

3	2 2	2	2 1
21	1 2	2	1 1
2	2 2	2	2 1
14	2 2	2	1 1
5	2 2	2	2 1
10	2 2	2	2 1
2	2 2	2	2 1
5	2 2	2	2 1
1	2 2	2	2 1
13	2 2	2	1 1
29	1 2	2	1 1
3	2 2	2	2 1
22	1 2	2	1 1
6	2 2	2	2 1
12	2 2	2	2 1
9	2 2	2	2 1
50	1 1	2	2 1
3	2 2	2	2 1
14	2 2	2	1 1
16	2 2	2	2 1
19	1 2	2	2 1
9	2 2	2	2 1
9	2 2	2	2 1
2	2 2	2	2 1
3	2 2	2	2 1
25	1 2	2	1 1
16	2 2	2	2 1
26	1 2	2	1 1
14	2 2	2	1 1
2	2 2	2	2 1
10	2 2	2	2 1
25	1 2	2	1 1
3	2 2	2	2 1
18	1 2	2	1 1
8	2 2	2	2 1
22	1 2	2	1 1
29	1 2	2	1 1
30	1 2	1	2 1
4	2 2	2	2 1
33	1 2	2	1 1
19	1 2	2	2 1
10	2 2	2	1 1
2	2 2	2	2 1
16	2 2	2	2 1
14	2 2	2	2 1
3	2 2	2	2 1

5	2 2	2	2 1
2	2 2	2	2 1
13	2 2	2	1 1
4	2 2	2	2 1
29	1 2	2	1 1
14	2 2	2	2 1
5	2 2	2	2 1
10	2 2	2	2 1
14	2 2	2	2 1
14	2 2	2	1 1
14	2 2	2	1 1
16	2 2	2	2 1
14	2 2	2	2 1
5	2 2	2	1 1
4	2 2	2	2 1
4	2 2	2	2 1
3	2 2	2	2 1
34	1 2	2	1 1
24	1 2	2	1 1
6	2 2	2	1 1
3	2 2	2	2 1
45	1 2	1	2 1
1	2 2	2	2 1
9	2 2	2	2 1
17	1 2	2	1 1
6	2 2	2	2 1
10	2 2	2	1 1
17	1 2	2	2 1
34	1 2	2	1 1
3	2 2	2	2 1
2	2 2	2	2 1
9	2 2	2	2 1
29	1 2	2	1 1
2	2 2	2	2 1
10	2 2	2	2 1
29	1 2	2	2 1
14	2 2	2	2 1
3	2 2	2	2 1
3	2 2	2	2 1
3	2 2	2	2 1
14	2 2	2	1 1
9	2 2	2	2 1
5	2 2	2	2 1
24	1 2	2	1 1
34	1 2	2	1 1
6	2 2	2	1 1

22	1 2	2	1 1
3	2 2	2	2 1
13	2 2	2	2 1
10	2 2	1	1 1
5	2 2	2	2 1
18	1 2	2	1 1
2	2 2	2	2 1
22	1 2	2	1 1
21	1 2	2	1 1
12	2 2	2	2 1
5	2 2	2	2 1
27	1 2	2	1 1
5	2 2	2	2 1
13	2 2	2	1 1
4	2 2	2	2 1
8	2 2	2	2 1
14	2 2	1	1 1
14	2 2	2	1 1
5	2 2	2	2 1
14	2 2	2	2 1
9	2 2	2	2 1
4	2 2	2	1 1
5	2 2	2	2 1
17	1 2	2	1 1
11	2 2	2	2 1
6	2 2	2	2 1
11	2 2	2	1 1
10	2 2	2	1 1
2	2 2	2	2 1
18	1 2	2	2 1
29	1 2	2	1 1
6	2 2	2	2 1
17	1 2	2	1 1
9	2 2	2	2 1
10	2 2	2	2 1
2	2 2	2	2 1
5	2 2	2	2 1
9	2 2	2	2 1
29	1 2	2	2 1
2	2 2	2	2 1
5	2 2	2	2 1
5	2 2	2	2 1
2	2 2	2	2 1
24	1 2	2	2 1
17	1 2	2	2 1
34	1 2	1	1 1

9	2 2	2	1 1
5	2 2	2	2 1
13	2 2	2	2 1
22	1 2	2	1 1
6	2 2	2	2 1
4	2 2	2	2 1
8	2 2	2	2 1
14	2 2	1	1 1
30	1 2	1	2 1
25	1 2	1	2 1
16	1 2	2	1 1
3	2 2	2	2 1
14	2 2	2	1 1
22	1 2	2	1 1
3	2 2	2	2 1
34	1 2	2	2 1
21	1 2	2	2 1
13	2 2	2	1 1
19	1 2	2	1 1
6	2 2	2	2 1
20	1 2	2	1 1
6	2 2	2	2 1
2	2 2	2	2 1
14	2 2	2	1 1
9	2 2	2	1 1
5	2 2	2	2 1
11	2 2	2	2 1
9	2 2	2	2 1
9	2 2	2	2 1
17	1 2	1	1 1
10	2 2	2	2 1
9	2 2	2	1 1
29	1 2	2	1 1
21	1 2	2	1 1
18	1 2	2	2 1
9	2 2	1	1 1
21	1 2	2	1 1
1	2 2	2	2 1
18	1 2	2	1 1
5	2 2	2	2 1
34	1 2	1	1 1
17	1 2	2	1 1
25	1 2	1	2 1
8	2 2	2	2 1
26	1 2	1	1 1
9	2 2	2	1 1

24	1 2	2	1 1
9	2 2	2	1 1
6	2 2	2	2 1
1	2 2	2	2 1
27	1 2	2	1 1
17	1 2	2	2 1
13	2 2	2	1 1
29	1 2	2	2 1
14	2 2	2	1 1
5	2 2	2	1 1
11	2 2	2	1 1
2	2 2	2	2 1
26	1 2	1	2 1
6	2 2	2	2 1
1	2 2	2	2 1
33	1 2	1	1 1
10	2 2	2	2 1
1	2 2	2	2 1
13	2 2	2	2 1
19	1 2	2	2 1
6	2 2	2	2 1
17	1 2	2	2 1
6	2 2	2	2 1
21	1 2	2	1 1
29	1 2	2	1 1
9	2 2	2	2 1
	2 2	2	1 1
66	1 2	1	2 1
33	1 2	2	1 1
22	1 2	2	1 1
20	1 2	2	1 1
16	2 2	2	2 1
57	1 2	1	2 1
22	1 2	2	1 1
11	2 2	2	2 1
35	1 2	1	1 1
24	1 2	2	1 1
11	2 2	2	2 1
14	2 2	2	1 1
14	2 2	2	2 1
2	2 2	2	2 1
14	2 2	2	2 1
17	1 2	2	2 1
9	2 2	2	2 1
9	2 2	2	2 1
14	2 2	2	2 1

12	2 2	2	1 1
25	1 2	2	1 1
22	1 2	2	1 1
8	2 2	2	2 1
3	2 2	2	2 1
30	1 2	2	1 1
2	2 2	2	2 1
33	1 2	2	2 1
42	1 2	1	1 1
9	2 2	2	2 1
8	2 2	2	1 1
9	2 2	2	1 1
9	2 1	2	2 1
17	1 2	2	1 1
14	2 2	2	2 1
29	1 2	2	2 1
33	1 2	2	1 1
22	1 2	2	1 1
10	2 2	2	2 1
2	2 2	2	2 1
17	1 2	2	1 1
13	2 2	2	1 1
3	2 2	2	2 1
18	1 2	2	2 1
19	1 2	2	1 1
8	2 2	2	2 1
13	2 2	2	1 1
35	1 2	2	1 1
9	2 2	2	2 1
2	2 2	2	2 1
29	1 2	2	1 1
14	2 2	2	2 1
5	2 2	2	2 1
5	2 2	2	1 1
10	2 2	2	2 1
24	1 2	2	1 1
29	1 2	1	2 1
17	1 2	2	1 1
3	2 2	2	2 1
22	1 2	2	2 1
12	2 2	2	1 1
2	2 2	2	2 1
17	1 2	2	2 1
3	2 2	2	2 1
4	2 2	2	2 1
17	1 2	2	1 1

9	2 2	2	2 1
22	1 2	2	1 1
17	1 2	2	1 1
6	2 2	2	2 1
24	1 2	2	2 1
19	1 2	2	2 1
9	2 2	2	2 1
19	1 2	2	2 1
2	2 2	2	2 1
38	1 2	1	2 1
1	2 2	2	2 1
5	2 2	2	1 1
35	1 2	2	2 1
9	2 2	2	2 1
3	2 2	2	2 1
11	2 2	2	2 1
10	2 2	2	2 1
2	2 2	2	2 1
9	2 2	2	2 1
6	2 2	2	2 1
12	2 2	2	2 1
10	2 2	2	2 1
3	2 2	2	2 1
10	2 2	2	2 1
9	2 2	2	2 1
9	2 2	2	2 1
32	2 2	2	1 1
9	2 2	2	2 1
29	1 2	2	1 1
30	1 2	2	2 1
5	2 2	2	2 1
29	1 2	2	1 1
6	2 2	2	2 1
9	2 2	2	2 1
10	2 2	2	2 1
2	2 2	2	2 1
17	1 2	2	1 1
19	1 2	2	1 1
2	2 2	2	2 1
10	2 2	2	1 1
2	2 2	2	2 1
4	2 2	2	2 1
5	2 2	2	2 1
11	2 2	2	2 1
24	1 2	2	1 1
17	1 2	2	2 1

9	2 2	2	2 1
27	1 2	2	1 1
9	2 2	2	1 1
2	2 2	2	2 1
4	2 2	2	1 1
11	2 2	2	1 1
2	2 2	2	2 1
11	2 2	2	2 1
14	2 2	2	2 1
2	2 2	2	2 1
25	1 2	2	1 1
5	2 2	2	2 1
17	1 2	2	1 1
26	1 2	2	1 1
5	2 2	2	2 1
19	1 2	2	1 1
2	2 2	2	2 1
9	2 2	2	2 1
25	1 2	2	1 1
14	2 2	2	1 1
32	1 2	2	1 1
17	1 2	2	1 1
3	2 2	2	2 1
22	1 2	2	2 1
6	2 2	2	2 1
26	1 2	2	1 1
6	2 2	2	2 1
4	2 2	2	2 1
14	2 2	2	2 1
2	2 2	2	2 1
16	2 2	2	2 1
4	2 2	2	2 1
12	2 2	2	1 1
6	2 2	2	2 1
17	1 2	2	1 1
3	2 2	2	2 1
17	1 2	2	2 1
14	2 2	2	2 1
9	2 2	2	2 1
22	1 2	2	1 1
34	1 2	2	1 1
3	2 2	2	2 1
11	2 2	2	2 1
24	1 2	1	1 1
5	2 2	2	2 1
16	1 2	2	2 1

20	1 2	2	1 1
5	2 2	2	2 1
2	2 2	2	2 1
20	1 2	2	2 1
1	2 2	2	2 1
27	1 2	1	2 1
25	1 2	2	1 1
4	2 2	2	1 1
1	2 2	2	2 1
14	2 2	2	2 1
9	2 2	2	2 1
17	1 2	2	2 1
14	2 2	2	2 1
17	1 2	2	1 1
41	1 2	2	1 1
1	2 2	2	2 1
17	1 2	2	2 1
4	2 2	2	2 1
4	2 2	2	2 1
20	1 2	2	2 1
2	2 2	2	2 1
29	1 2	2	1 1
9	2 2	2	2 1
9	2 2	2	2 1
3	2 2	2	2 1
2	2 2	2	2 1
22	1 2	2	2 1
3	2 2	2	2 1
25	1 2	1	2 1
17	1 2	2	2 1
26	1 2	1	1 1
5	2 2	2	1 1
1	2 2	2	2 1
2	2 2	2	2 1
2	2 2	2	2 1
17	1 2	2	2 1
4	2 2	2	2 1
9	2 2	2	1 1
2	2 2	2	2 1
19	1 2	2	2 1
14	2 2	2	2 1
5	2 2	2	2 1
9	2 2	2	2 1
2	2 2	2	2 1
2	2 2	2	2 1
3	2 2	2	2 1

14	2 2	2	1 1
1	2 2	2	2 1
24	1 2	2	1 1
6	2 2	2	2 1
22	1 2	2	1 1
8	2 2	2	2 1
14	2 2	2	2 1
2	2 2	2	2 1
3	2 2	2	1 1
17	1 2	2	1 1
17	1 2	2	2 1
8	2 2	2	2 1
9	2 2	1	2 1
33	1 2	2	1 1
5	2 2	2	2 1
5	2 2	2	1 1
9	2 2	2	1 1
4	2 2	2	2 1
1	2 2	2	1 1
25	1 2	1	2 1
26	1 2	2	1 1
6	2 2	2	2 1
22	1 2	2	1 1
2	2 2	2	2 1
2	2 2	2	2 1
2	2 2	2	2 1
1	2 2	2	2 1
1	2 2	2	2 1
29	1 2	1	1 1
4	2 2	2	2 1
2	2 2	2	1 1
2	2 2	2	2 1
21	1 2	2	1 1
16	1 2	2	1 1
10	2 2	2	2 1
17	1 2	2	2 1
10	2 2	2	2 1
17	1 2	2	1 1
11	2 2	2	2 1
6	2 2	2	2 1
22	1 2	2	2 1
4	2 2	2	2 1
9	2 2	2	1 1
10	2 2	2	1 1
29	1 2	2	1 1
2	2 2	2	2 1

17	1 2	2	1 1
9	2 2	1	1 1
29	1 2	1	2 1
20	1 2	1	1 1
9	2 2	2	1 1
2	2 2	2	2 1
8	2 2	2	2 1
17	1 2	2	2 1
1	2 2	2	2 1
5	2 2	2	1 1
14	2 2	2	2 1
17	1 2	2	2 1
5	2 2	2	2 1
5	2 2	1	1 1
3	2 2	2	2 1
3	2 2	2	1 1
14	2 2	2	1 1
10	2 2	2	2 1
3	2 2	2	2 1
2	2 2	2	2 1
17	1 2	2	1 1
21	1 2	2	2 1
24	1 2	2	2 1
5	2 2	2	2 1
2	2 2	2	2 1
3	2 2	2	2 1
22	1 2	2	1 1
10	2 2	1	1 1
1	2 2	2	2 1
25	1 2	2	1 1
2	2 2	2	2 1
26	1 2	2	1 1
25	1 2	1	1 1
14	2 2	2	2 1
12	2 2	2	1 1
5	2 2	2	2 1
14	2 2	2	1 1
3	2 2	2	2 1
2	2 2	2	2 1
5	2 2	2	2 1
3	2 2	2	2 1
29	1 2	1	2 1
2	2 2	2	2 1
10	2 2	2	2 1
5	2 2	2	2 1
10	2 2	2	2 1

33	1 2	2	2 1
2	2 2	2	2 1
6	2 2	2	2 1
11	2 2	2	2 1
25	1 2	2	1 1
19	1 2	2	1 1
17	1 2	2	1 1
6	2 2	2	2 1
5	2 2	2	2 1
13	2 2	2	2 1
9	2 2	2	2 1
9	2 2	2	2 1
3	2 2	2	2 1
17	1 2	2	2 1
3	2 2	2	2 1
10	2 2	2	2 1
1	2 2	2	2 1
22	1 2	2	2 1
11	2 2	2	1 1
24	1 2	2	2 1
10	2 2	2	1 1
8	2 2	2	2 1
3	2 2	2	2 1
1	2 2	2	2 1
6	2 2	2	2 1
25	1 2	2	1 1
13	2 2	2	1 1
10	2 2	2	2 1
16	2 2	2	1 1
22	1 2	2	1 1
22	1 2	2	1 1
5	2 2	2	1 1
29	1 2	2	1 1
9	2 2	2	2 1
3	2 2	2	2 1
19	1 2	2	1 1
17	1 2	2	1 1
	2 2	2	2 1
19	1 2	2	1 1
6	2 2	2	2 1
2	2 2	2	2 1
14	2 2	1	1 1
6	2 2	2	2 1
1	2 2	2	2 1
13	2 2	2	1 1
5	2 2	2	2 1

9	2	2	2	2	1
24	1	2	2	1	1
3	2	2	2	2	1
9	2	2	2	2	1
22	1	2	2	1	1
1	2	2	2	2	1
2	2	2	2	1	1
9	2	2	2	1	1
38	1	2	1	1	1
5	2	2	2	2	1
25	1	2	2	1	1
11	2	2	2	2	1
17	1	2	2	2	1
17	1	2	2	2	1
2	2	2	2	2	1
2	2	2	2	2	1
17	1	2	1	2	1
3	2	2	2	2	1
14	2	2	2	1	1
8	2	2	2	2	1
8	2	2	2	2	1
29	1	2	2	1	1
9	2	2	2	2	1
14	2	2	2	1	1
9	2	2	2	2	1
9	2	2	2	1	1
9	2	2	2	2	1
4	2	2	2	2	1
14	2	2	2	2	1
14	2	2	2	1	1
2	2	2	2	2	1
13	2	2	2	1	1
3	2	2	2	2	1
9	2	2	2	2	1
50	1	2	2	1	1
10	2	2	2	2	1
18	1	2	2	1	1
9	2	2	2	2	1
9	2	2	2	2	1
4	2	2	2	2	1
9	2	2	2	1	1
6	2	2	2	2	1
16	1	2	1	1	1
50	1	2	2	1	1
9	2	2	2	2	1
29	1	2	2	1	1

13	2 2	2	2 1
9	2 2	2	1 1
17	1 2	2	1 1
20	1 2	2	1 1
21	1 2	2	1 1
14	2 2	1	1 1
26	1 2	2	1 1
9	2 2	2	2 1
6	2 2	2	2 1
25	1 2	2	2 1
	2 2	2	2 1
13	2 2	2	1 1
4	2 2	2	2 1
4	2 2	2	2 1
17	1 2	2	2 1
22	1 2	2	1 1
17	1 2	2	1 1
3	2 2	2	2 1
3	2 2	2	2 1
10	2 2	2	2 1
3	2 2	2	2 1
3	2 2	2	2 1
11	2 2	2	2 1
6	2 2	2	2 1
2	2 2	2	2 1
17	1 2	2	2 1
6	2 2	2	2 1
11	2 2	2	2 1
3	2 2	2	2 1
12	2 2	2	2 1
14	2 2	2	2 1
9	2 2	2	2 1
4	2 2	2	2 1
2	2 2	2	2 1
11	2 2	2	2 1
11	2 2	2	2 1
9	2 2	2	2 1
24	1 2	2	2 1
6	2 2	2	2 1
12	2 2	2	2 1
25	1 2	1	2 1
14	2 2	2	1 1
5	2 2	2	2 1
22	1 2	2	1 1
14	2 2	2	2 1
17	1 2	2	1 1

4	2 2	2	2 1
2	2 2	2	2 1
2	2 2	2	2 1
9	2 2	2	2 1
10	2 2	2	2 1
3	2 2	2	2 1
38	1 2	2	1 1
9	2 2	2	1 1
9	2 2	2	2 1
33	1 2	1	1 1
8	2 2	2	2 1
5	2 2	2	2 1
22	1 2	2	2 1
6	2 2	2	2 1
17	1 2	2	1 1
6	2 2	2	1 1
18	1 2	1	1 1
26	1 2	1	1 1
17	1 2	2	1 1
3	2 2	2	2 1
50	1 2	2	1 1
22	1 2	2	1 1
11	2 2	2	2 1
25	1 2	2	2 1
17	1 2	2	1 1
11	2 2	2	2 1
10	2 2	2	2 1
9	2 2	2	1 1
10	2 2	2	2 1
3	2 2	2	2 1
17	1 2	2	1 1
9	2 2	2	2 1
13	2 2	2	1 1
6	2 2	2	1 1
9	2 2	2	2 1
9	2 2	2	2 1
11	2 2	2	2 1
17	1 2	2	1 1
21	1 2	2	2 1
24	1 2	2	2 1
22	1 2	1	1 1
11	2 2	2	2 1
14	2 2	2	1 1
1	2 2	2	2 1
11	2 2	2	1 1
29	1 2	2	1 1

9	2 2	2	2 1
13	2 2	2	2 1
25	1 2	1	1 1
14	2 2	2	1 1
2	2 2	2	2 1
14	2 2	2	2 1
10	2 2	2	1 1
4	2 2	2	2 1
29	1 2	2	1 1
6	2 2	2	2 1
6	2 2	2	2 1
25	1 2	1	1 1
12	2 2	2	1 1
3	2 2	2	2 1
14	2 2	2	2 1
4	2 2	2	2 1
14	2 2	2	1 1
17	1 2	2	2 1
3	2 2	2	2 1
6	2 2	2	1 1
14	2 2	2	2 1
4	2 2	2	1 1
29	1 2	2	2 1
10	2 2	2	2 1
10	2 2	2	1 1
29	1 2	2	1 1
18	1 2	2	1 1
6	2 2	2	2 1
10	2 2	1	1 1
41	1 2	2	2 1
35	1 2	2	2 1
6	2 2	2	2 1
34	1 2	2	2 1
26	1 2	2	2 1

Zeit_bis_Tod	Austrittsort_Umcodiert	Unfallort_umcodiert	S_100
		5	1.6800
		5	.1440
		10	.2690
		7	.0670
		10	9.4100
		7	.3560
	3	5	.5220
		7	.1270
	8	5	.0730
		14	.0490
		14	.3520
	8	10	.4890
		10	.1260
		10	.1340
	8	14	1.5200
	7	5	.3560
		7	.2040
	8	14	.0960
	7	5	.3610
	5	5	.0640
	8	7	.0560
	7	10	.1130
		7	.2530
		5	.1000
1		7	3.1100
			.5690
		10	.3760
		10	.1040
		5	.0720
	7	7	.2140
		5	.1010
	7	7	.3090
		10	.1190
		5	.1020
	7	10	.1080
		5	.0900
		10	.1050
		10	2.6500
		5	.2380
		5	.1520
		5	.1150
		10	.0650
	5	10	.2120
		7	.4830
		5	.1710

		14	.6680
		7	.2090
		5	.7160
		10	.1470
		10	.9420
		10	.0900
		10	.2200
		10	.6750
		5	.3980
		5	.4970
		5	.1100
		10	16.4900
		10	.2060
		10	.3880
		7	.0760
		10	.1190
		7	.3050
		5	.4530
		10	.2260
		14	1.6600
		10	.3100
			5.3000
	7	7	.1760
		14	2.7700
2	7	7	1.2300
		7	.1020
		10	.2130
		5	1.1700
		14	.0680
		7	.6730
		7	.1240
		10	.0730
		7	1.0700
		5	.2300
		10	6.7800
		5	2.2000
		14	1.4500
		7	.1500
		10	.4750
		5	.1120
		5	.1240
		5	.0900
		7	.4850
		5	2.5900
0		14	8.3700
		5	.0930

		7	.1090
		7	.4720
		7	.1120
		14	.2620
		5	.1900
		14	.0880
864	7	5	.1970
7		7	.3050
		14	.0480
		14	.1570
		5	.2290
		10	.0720
		7	.0690
		7	.2750
		7	.6050
		14	.1490
5		7	.4910
		10	1.3400
		14	3.3300
	7	10	.7150
		10	.2380
		5	.0840
		14	.1120
		5	.0360
		10	.5630
		5	.4000
		14	.2040
		14	1.4100
		7	.0790
		5	.2280
		7	.3390
		10	.0960
		5	.1730
		14	.3970
		14	.8100
		7	1.1400
		5	3.1100
		5	.1780
		7	.2010
		5	.0680
			1.6400
		14	.5540
		7	.2350
		10	.1380
			.3080
1		5	1.8200

		7	.1500
		14	1.1300
		14	.1680
		5	.0630
		7	.0930
237		7	.3080
		5	12.1000
		14	17.0800
		10	.1120
		14	.2460
		7	1.2800
		14	.5940
0		5	15.2300
		14	5.6400
		5	.3070
		14	.3150
		5	.3010
		7	.4800
		7	.2210
		5	1.2500
		7	.1110
		14	3.6200
		10	.5020
		14	3.0600
		14	1.5000
		14	1.0100
		5	5.7000
		14	.9030
		7	.4200
		10	.4260
		7	1.5000
		14	1.7300
		7	.9470
		14	.8200
		14	.1810
		10	.0720
		14	.0800
27		5	.9210
		14	.2660
		14	1.9800
		14	.7380
		14	.5450
	3	5	.4410
		10	.6580
	3	14	.7390
	3	10	.6330

	14	1.5700
	5	.0780
	14	2.1700
	5	.1490
	14	.3070
	5	.8990
	14	3.5200
	5	.9530
	14	5.4000
	5	.6540
	14	.1290
	14	.0580
	5	.4520
	14	.1590
	5	.2010
	7	.0490
	14	.6510
	7	1.9300
3	7	.2090
	10	.3120
	14	.4670
	5	.6410
	10	.0990
	10	.0800
	14	.4490
	5	.3350
	5	.2880
	5	.1220
	14	.4200
	7	.8180
	14	.8490
3	10	2.5000
	5	.7720
	7	.1730
	7	1.1800
7	14	.9770
	14	2.8300
	14	.1260
	10	.9000
	10	2.2600
	7	.3980
	14	1.0400
	5	.1880
	5	.7590
	5	.3010
	5	2.8400

47		5	3.0100
		14	.5210
		14	.1770
		5	.3820
		5	.6150
		14	.3100
		5	.3880
		14	.6360
		14	.5620
		14	.5740
	5	5	.2320
		10	.4320
		14	2.5300
		14	.0820
		14	.8990
1		14	1.1200
	5	14	.0350
		10	.5410
		14	.2190
2		10	8.3800
		10	1.2800
		10	.7390
		14	.6910
	5	5	.9300
		14	2.2500
		14	5.1100
		14	.9550
0		14	3.7300
		14	.1270
		14	.4180
		7	.4660
		14	1.3000
		10	.1020
		10	.4280
		14	6.7800
		14	.5940
		7	.1580
		14	.2000
		14	1.5400
		14	6.4900
		14	4.0100
0		5	3.1400
		14	.2220
		14	.5300
			.3820
		14	1.3000

		14	3.4000
1		14	2.4500
		5	.1740
		7	.7730
		5	1.7600
		14	1.2900
		5	.1670
		14	.9260
		14	.2850
		14	.1160
		7	.6330
		14	.4460
		5	5.9400
		7	3.0600
		7	.3560
		14	.5250
	5	5	.0980
		7	.4650
		10	.7140
		14	1.0300
		14	.0550
		5	.6600
		7	5.9000
		5	2.5600
		7	1.4000
		10	.9030
	7	10	.4090
		14	.7230
		14	.5100
		14	1.4000
		10	.7530
	7	5	3.7600
		10	.1280
		10	.4100
		10	1.4500
		14	.0780
		14	.3610
		5	.0870
		14	.8910
		10	.5910
		14	4.8500
	7	14	3.6200
		5	1.2600
		14	.2830
		5	.4770
		10	.5230

		14	1.6500
		14	.2850
		10	.8190
		5	.0670
	7	14	1.6000
		5	.7930
	7	7	32.5100
		14	3.9000
		10	.2200
		5	.2160
	5	5	.2550
		14	.0560
		7	2.0600
		5	.5160
		14	1.3800
		5	.2260
		10	.1260
		5	3.1800
	7	14	.2320
		5	1.3600
	7	14	.3890
	7	5	.3750
		10	.3740
		14	.9270
		14	.5180
		14	.2820
	7	10	2.7100
	3	14	3.5400
		5	1.6200
	5	14	.2260
		5	.2000
		5	1.2100
		5	2.2700
	7	7	1.1600
		14	8.2800
	3	14	.1240
		14	1.5900
		14	.0520
		14	.4020
0		5	13.2900
		10	.9220
		5	.7020
		14	.6120
		14	.0440
		10	.6480
		14	.1770

		14	1.8700
		5	8.3100
	3	7	.7190
		14	.0580
		5	.8100
		14	.0660
		5	.1700
		7	.3570
		14	.4050
		14	2.7100
		14	.0820
	5	14	.8170
		14	.1100
		14	2.2000
		7	1.0900
		5	.2180
		14	.0450
		10	.6490
		14	.3250
		5	.1510
0		7	8.2300
		7	.1840
		5	.1790
		14	.7870
		14	1.2600
	5	7	.1570
		5	.6890
	5	10	.1170
1		14	7.8400
		5	4.2200
		14	1.2900
		5	.8500
		10	.2560
		14	2.1900
		14	.2500
	3	10	.1260
		5	.5250
		5	.0920
		7	.0740
		7	1.0600
		7	.4300
		14	.0830
		5	.4410
	7	14	.0680
		10	.2680
	5	14	.2210

	3	5	.6000
		14	3.6000
		10	.3940
		14	.8160
	5	5	.1170
		7	.1990
		14	.2120
		5	.2820
		14	5.2900
		14	.1010
		14	.0780
	3	10	.3600
		5	1.8100
		10	.2610
		14	.4140
		14	.3660
		5	.4400
		7	.1000
	5	10	.1300
	3	14	1.0100
		5	.7530
		14	.0860
		10	.4360
		5	.7170
		14	.1060
		10	.3030
		14	3.9000
		14	.2160
	5	7	.0710
		7	.4070
		5	.5010
		7	.3500
		14	2.3600
		5	.1630
		14	.5650
		7	1.5600
		14	.2430
		5	.7640
		14	.1700
	5	14	.0890
2		10	.7620
		5	2.6400
		7	.2560
		7	.0980
		5	.1870
		14	.9690

		5	.3940
12		14	2.1300
		14	.7330
		14	7.7600
		14	6.7600
		10	.4420
		5	.1560
		5	1.8900
		14	.3300
		10	.3140
		14	.0810
		5	1.7400
		7	1.3600
		5	.5970
		14	.2210
		7	.2600
		10	3.2900
1		7	2.5000
		5	.0810
1		7	16.1000
		5	.1220
		7	1.1000
		14	.1030
		7	.2590
		5	1.5600
		5	.1880
		14	.1430
		7	.1420
		10	1.1700
1		7	.8800
		10	.0910
		10	2.6300
	5	5	.5140
		5	6.7700
		10	.1750
		7	.2770
		10	.1500
		14	2.3000
		7	1.8800
0			5.0200
		5	1.0700
		10	.6120
		10	2.2300
		5	.1690
		10	.5490
		10	2.5800

		5	.2310
		5	.2870
		7	.1800
	3	5	1.7500
	5	14	.3650
	5	14	.6960
1		5	11.7000
		5	.0790
		7	.6850
5		10	5.6000
2			2.2500
		7	1.9500
	5	14	.2290
		5	1.1400
		7	.8480
2		10	2.2900
		7	1.0500
		10	.8860
		10	.1250
	5	5	.0820
		14	.0950
		10	.2670
		10	.8490
		7	.6520
		5	.1240
		14	1.1300
		5	2.3900
		14	.1360
		14	1.2100
	7	14	1.3400
		7	.7500
		7	.2910
	7	5	.0660
		7	.1590
		10	.2590
	7	14	1.1700
	7	5	1.3100
		10	.1540
	7	7	1.3000
	7	14	3.0900
	7	7	.1730
	7	14	.0990
	7	5	.5750
	7	14	.5740
		7	.2530
	7	5	.1810

		5	.1650
		10	.4860
3		7	.4670
	7	10	.1540
	7	7	.2630
	7	7	.2030
	7	5	.1130
	5	10	.0590
		14	.1160
	7	7	.3680
	3	7	.1580
	3	14	.4030
		14	.0970
212	5	5	2.3800
		5	1.0600
	5	5	.1790
	3	10	.3020
		14	.4320
		5	.1910
	7	14	.8570
	5	10	.0570
	7	14	.0680
	5	7	.4590
		10	2.6000
	7	10	.1070
		10	.1020
	5	10	.4020
		10	.1790
		5	.3880
	3	14	.1710
	7	14	.4270
	7	14	.5350
	7	5	.5050
	7	5	7.1700
		10	.9840
		5	.7400
	7	14	.1540
3		7	.4890
		5	2.9400
		5	6.4200
	7	10	1.6900
	7	5	.1240
	5	14	.2610
	7	10	.3010
		10	1.2800
		10	.5410

		14	6.4300
		14	.6260
0		5	11.3300
		10	.9080
		5	.8910
	3	14	.5050
	7	5	.3250
	7	5	.8750
		14	1.3600
		14	3.3400
		5	1.0000
		14	.9400
		7	.1450
	7	10	.7850
		14	2.5100
		10	.1480
		10	.5260
	7	10	.1520
		10	.1100
		14	7.1500
		14	.6710
	7	14	.2930
	3	5	1.7600
		14	4.7500
	7	14	.1020
	3	10	5.6200
	7	14	.2790
	3	14	.9350
	7	5	.2610
		5	.2900
	7	5	.1200
		10	.4560
	7	5	.0630
	3	7	1.0100
		14	.5970
		7	.1080
	7	5	.1600
		10	1.7800
		14	.3010
		14	4.1900
		10	3.4300
		14	.9060
		14	1.8400
	7	5	.0450
	7	14	.3720
		14	.1000

	7	14	.1500
		7	1.2100
	5	14	.4550
	3	14	.3590
	3	10	.1570
		14	.7210
	3	7	.2070
	7	7	1.4100
	7	7	.1140
		14	1.7200
		7	2.9700
	7	14	.1630
		7	2.8100
	7	7	3.1500
		5	1.3000
		7	.2600
0		14	10.7200
		14	.0730
	7	14	1.1500
		10	.2010
		14	.9930
		14	.0860
		14	.2070
	7	10	.4870
	5	7	.2620
		7	.9500
	5	5	.1200
		14	.1140
	3	14	.3370
	3	14	.0840
	7	14	.6100
		10	1.4000
	5	14	.1640
	7	14	3.7700
		10	3.2100
		5	2.0100
		5	5.3200
0		14	2.8800
		10	1.3100
		14	5.1900
	7	14	1.0600
		14	.3860
	7	14	.1190
	7	7	.0950
	3	14	.3740
	7	14	.5650

	3	14	.2040
	7	5	.3340
		14	1.1000
	7	7	.3410
		14	.2800
		14	1.7800
	5	7	1.2700
	3	10	.5310
		7	.1410
	3	7	.4900
		14	2.4800
		14	1.0100
		14	.1450
		14	1.2500
		14	.2970
		10	.0690
	7	5	.0620
		14	5.4600
		7	.5870
		7	.2510
	3	10	.1420
0		14	4.1200
	7	14	.0640
	7	14	.0570
		7	1.5300
	3	14	.0280
		7	.4230
		5	.0780
		14	1.3800
	3	14	.3970
	7	14	1.7700
		5	1.1400
		10	1.0500
	7	5	.7740
	5	7	.0850
		5	3.2800
		14	.6450
	7	14	.8790
	7	14	.0790
	7	14	.2010
		14	1.5000
		5	.1760
	7	14	.5590
		14	4.4000
		14	5.7400
	3	7	1.5400

		5	.8020
	7	14	1.0000
		14	1.5000
5		7	.6590
		5	.1450
	7	5	1.0300
	7	14	.1910
		5	.6640
		14	2.4700
	7	14	2.9800
	7	10	.9420
		14	2.0900
	5	14	.4670
		14	.7090
	7	10	.4460
	5	7	2.3600
1		14	9.5600
		14	.2390
		14	.0590
	3	10	.0730
		10	.4480
		14	.1480
		14	.0690
		14	.4500
	3	7	1.0500
	3	14	.6400
		5	1.6800
		5	.4790
	7	14	.3250
		14	.4730
	3	14	1.2100
		14	.2240
		14	.1790
		14	2.3800
	3	14	.6340
	7	14	.7390
		14	2.8100
	5	5	1.5200
	7	5	1.0500
	7	14	.2960
	7	14	.5620
	7	14	.8100
		5	.1450
		14	3.4500
		14	1.6700
1		14	18.3300

		10	1.9900
		10	.6940
		7	.0790
		7	7.7800
	5	7	1.1900
		10	.3750
	7	10	1.9800
2		14	5.2400
0		14	39.0000
0		7	39.0000
	3	7	.2480
	7	14	.1120
		14	1.0100
		14	6.2700
	7	14	1.2100
		14	.5380
		14	.2110
	3	5	.1300
		10	.3840
	7	10	.3920
	7	7	.2710
		14	.2100
	5	14	.2750
		5	.1890
		7	1.6300
	7	14	.2560
		14	.4160
	7	5	4.1200
	7	5	.0500
8		14	7.4300
		7	.5750
		5	.1840
		7	2.6700
	3	7	.4090
	7	7	1.8200
4		14	1.3700
		7	1.6900
	7	5	.0550
	3	14	2.3600
		14	.6800
3		5	15.0300
		14	3.8400
3		7	.2600
		7	.5060
3		7	1.9000
		14	1.9900

		14	2.1700
		14	.1980
	7	14	.9350
		10	.4630
	3	7	.1190
	3	7	1.0300
		10	1.7200
		14	.6640
		10	1.0800
	3	10	1.0900
		7	.6830
	7	10	.1650
0		7	7.9900
	7	5	2.2200
	5	14	.1210
10		5	2.6200
		7	.2450
	7	5	.5090
		14	.0640
		14	2.5400
		14	.2840
	3	14	3.6700
	7	14	.2460
	7	5	1.1500
		10	2.6700
	7	7	.1750
			.1720
0		14	16.2100
		10	2.7600
		14	3.3100
		10	1.2100
	7	14	.1300
0		10	21.7100
		14	2.1700
		10	2.9100
1		10	2.3100
		14	.3560
		10	.1730
		14	2.2400
		5	.1060
	7	7	.2860
	7	14	.7590
	5	5	1.1800
		14	1.0200
	7	5	1.6600
		14	.7300

		14	.7080
		10	1.4600
	3	14	.8800
	7	10	.9990
	5	5	.2890
		10	2.7000
	7	14	.0840
		10	2.2700
1		5	1.4200
	7	10	.0620
		14	1.5000
		5	.2020
0		7	.1210
	3	14	5.3300
		10	.5180
		14	.1020
		14	.3800
		14	3.1800
	7	5	1.5500
	3	5	.2310
		14	.2370
	7	10	1.1900
	7	14	.4760
		14	.3210
		14	1.6400
	7	10	2.0200
		14	1.4500
		5	4.7900
		14	2.7500
	7	7	.0650
		14	1.4600
		5	1.3000
		5	.6880
		14	.3350
		14	3.8900
		10	2.3900
0		14	1.3600
	3	14	2.6800
	7	14	.0890
		10	.2300
		10	1.9900
	7	14	.6430
		10	2.2600
	7	14	.2350
	5	7	.1760
	3	7	.5180

		5	.1440
		10	1.3500
		10	1.4700
		5	.6300
	3	5	.9500
		10	.1350
		10	3.8100
	3	14	2.3300
	7	14	.1550
0		10	39.0000
	7	14	.2420
		7	.8030
		14	2.1500
	7	14	.8550
	5	14	.3440
	7	5	.2480
	5	7	.4360
	5	14	.5710
		14	.6780
	7	5	.2910
		5	5.1100
		10	1.5500
	3	14	.7320
		14	.9300
		7	.2710
	7	10	.5020
		14	4.2900
		5	.0800
		14	5.4000
		14	.5660
		14	3.0200
		10	9.5900
	7	14	1.0500
		10	.4090
		14	3.7700
	5	5	.0630
		5	2.1200
	3	7	.3530
	7	14	.1500
	3	7	1.2900
	5	10	.2760
		5	.0570
		14	.2220
	7	14	1.4700
	3	5	1.5000
		5	1.9000

		5	1.2600
		5	3.0200
		7	3.9400
	5	14	.0900
		5	1.0200
	3	5	.3640
	7	14	.1010
	3	10	3.2100
		14	.6660
	7	14	.2010
		10	.7150
	7	14	.0790
		14	1.0200
		14	4.8100
	7	5	.2610
		14	1.6000
	5	5	.5530
		7	.4650
		10	.1290
		14	.2120
		10	.3530
		5	.9280
	7	14	.8410
		14	.2850
	7	5	.0610
		5	1.1500
	7	14	1.1000
		5	1.5100
	7	10	1.2400
	7	10	.3450
		14	.2730
	7	14	.4540
		14	.4440
	7	5	.0480
		7	4.0800
	7	14	.0920
	3	7	.7560
	3	14	1.1900
		14	1.5000
		5	1.5400
		5	2.7100
	7	14	.5970
	7	7	.5610
1		14	3.6100
		7	1.5300
	7	7	1.5300

		14	1.7400
	5	10	.8890
	7	7	.2970
	7	10	.3170
	7	14	.2430
0		14	24.6500
		7	.4610
	7	7	.2790
	5	10	.0990
	7	5	1.9100
	7	5	.8900
		14	5.0500
		5	.5450
	3	7	3.5000
		10	8.5200
	7	14	.0320
	7	7	.1800
		7	.5400
	7	7	.1770
		14	.2620
	7	14	.0580
		14	2.3100
	3	10	.1660
		5	6.1900
	7	10	.7480
	7	14	.0840
	7	14	6.8700
	7	14	.2060
1		7	1.6100
		14	5.1300
1		14	9.0000
		14	1.5100
	7	5	.1600
	5	14	.0340
	5	14	.7860
		5	3.7600
	7	5	.5380
		14	.4850
	5	5	.1320
	7	7	.6520
		5	.5810
	7	10	3.8100
	3	7	.2570
	7	14	.1910
	5	14	.0560
	5	14	.2380

	3	14	.9420
	7	5	3.2800
		14	1.4300
	7	14	.6000
		14	5.5800
	7	5	.1700
		7	.1880
	7	5	.0710
		7	.4250
		14	5.8500
		10	.3170
	7	14	.3250
0		5	39.0000
		10	.7170
	7	10	.5140
		10	.5120
		7	.8850
		10	.7870
	7	5	.1810
0	3	10	6.1200
		10	.7810
	3	10	.7790
		14	1.5100
	7	14	.0560
	7	5	.1450
	7	14	.0860
	7	14	.7100
	5	10	.0840
2		7	2.1200
	7	14	.9430
	7	14	3.0400
	7	14	.2360
		5	2.0400
		5	1.2400
	5	14	.6950
		10	.1870
	5	7	.1590
		10	4.0400
		10	.3570
	7	14	1.0900
		10	.3600
	7	14	2.2900
		10	.1220
		5	5.5000
		5	8.4400
	7	10	.1990

		14	.7790
1		5	3.8700
0		10	12.1800
1		14	1.3200
10		7	.9160
	5	5	.7320
	7	14	2.3600
	3	10	.6200
	5	5	.0850
		14	3.3300
		14	1.0500
	3	5	2.5100
	7	5	.2410
2	7	7	6.7900
	7	14	.2420
		5	.2770
		14	3.0100
	7	7	.0500
	3	14	.8670
	7	10	.0730
		5	1.6500
		7	5.3200
		10	5.0900
	7	5	.2940
	5	14	.0510
	7	14	.1490
	3	5	2.3400
5		5	3.3300
	7	10	.9120
		7	.1340
	7	7	.0600
		5	3.3800
1		14	13.0300
		10	1.8400
	3	10	.9060
		7	.8440
		5	7.2800
	7	7	.6380
55	7	14	15.0100
		14	.8700
	3	14	.1250
0		5	1.6200
	7	7	.2840
		14	6.1900
	7	14	.3680
		5	.2980

		10	1.9000
	7	10	.2020
		5	.9120
	7	5	.6930
		10	.1150
		10	1.0800
		5	.8060
	7	14	.1820
	5	14	1.1200
	3	14	5.5100
	3	14	.3030
	5	7	.1130
	7	14	1.2900
		14	1.6900
	5	14	.3140
	5	14	.0640
	5	10	.1150
		14	5.6700
	3	5	.0460
	7	5	1.7500
		14	.6730
		10	.2390
	7	14	.1750
	5	10	.1230
	3	7	.4910
		5	1.7200
		14	.2640
		5	.1870
		7	.4240
		5	3.9400
		14	4.0700
	7	10	.1230
		10	1.3100
		10	2.9300
	7	14	.2990
		14	1.4400
		14	5.3700
		14	.2850
		14	1.5200
		14	.4770
	5	14	.5490
3		14	9.0300
	7	10	1.6300
	5	7	.0380
		14	.7140
		10	.2030

	7	14	.6530
		14	1.2000
	5	7	2.2000
		14	.4300
		7	1.1800
	7	7	.0850
	5	7	.4000
		10	1.4400
3		7	1.0400
	5	14	.1070
		7	.2520
	7	14	.7530
		14	.9960
		5	5.1400
	7	5	.0680
	7	14	.1930
0		7	27.8400
	7	7	.6120
		7	.6430
		14	.8500
		5	.2590
		5	7.3200
	7	14	1.9300
		10	.6930
	7	5	.3660
		14	.6720
		10	.9650
		5	.5000
	7	14	.3420
	3	14	1.5100
	7	7	.0790
		14	1.3900
	5	14	.1890
		10	.6900
		14	6.6700
	7	14	.9460
		5	2.8300
		5	2.7200
	7	5	1.3900
	7	5	.0970
		14	.3460
	7	10	1.8100
5		14	2.6700
		5	4.2300
		14	.2160
	3	5	.1960

	7	10	.4280
	7	14	.4370
		14	.8090
		5	.7980
		5	.7480
1	7	5	12.2200
		7	4.7500
		14	3.7500
	7	14	.4840
		14	2.0100
			1.8900
		10	.4280
	7	10	1.2700
	5	10	.1800
	3	14	3.1700
		14	1.9700
		14	1.4200
	7	14	.1740
	3	14	.1420
	7	10	.9550
	7	14	.2410
		14	.2400
		5	4.9900
	7	5	.2440
	7	14	.0380
		14	5.6700
	5	14	3.1200
	3	10	.2970
	7	14	.3330
		10	1.6700
		14	.3760
		14	.1710
		14	.1300
	5	5	.7070
	7	14	.4940
		5	.8700
		5	2.7100
		10	3.5200
		14	.4710
	3	10	1.9000
0		5	19.4300
		7	1.8200
	7	10	1.5300
	3	10	3.6700
		14	1.5600
		5	1.7000

		10	.7380
	5	14	.0550
	5	14	.5450
		14	.7830
		5	.8860
	5	5	.2470
		14	9.3600
		5	1.3300
	3	14	.2140
1		7	3.2900
	7	14	3.2100
		14	.1950
		14	.6460
	7	14	.9530
		7	2.1300
	7	10	.6590
0		7	8.3700
2		7	.6480
		5	.6060
	7	5	1.1400
		14	9.5100
		7	1.0400
	7	10	.0730
		10	.6380
	3	5	5.7200
	7	7	.1530
	7	7	.2390
	7	5	1.2100
		10	3.4400
	7	14	.2970
		14	.6150
		7	4.9900
		14	.7180
		14	.2540
	3	14	.2420
	5	14	.0620
		14	.6600
		5	1.2700
		10	1.6000
	7	5	3.7200
1		10	8.8800
	7	5	.3630
		14	2.2700
	7	7	.0660
		5	5.1800
		7	5.7400

		10	.3440
		10	3.1100
1		7	2.2000
		14	4.5100
	5	10	1.3900
		10	2.8700
		10	2.3000
		10	1.3300
		14	.6040
	7	14	.1570
		5	1.8300
1		5	2.4800
		7	3.1200
	7	14	.0640
		7	.3710
		10	.3440
		7	1.2800
	7	10	5.5300
	5	14	.3450
	3	14	1.4000
		10	1.8200
		10	.2530
		7	.3510
		14	1.3800
	7	5	5.4100
		14	2.6000
		5	.7510
	5	5	.4570
1		5	15.0900
		7	2.8200
		5	1.1900
		7	.1950
		10	1.4700
		7	.2850
